# Genes and pathways correlated with heat stress responses and heat tolerance in maize kernels

**DOI:** 10.3389/fpls.2023.1228213

**Published:** 2023-08-17

**Authors:** Yan Chen, Tingting Du, Jie Zhang, Shoukun Chen, Junjie Fu, Huihui Li, Qin Yang

**Affiliations:** ^1^ State Key Laboratory of Crop Gene Resources and Breeding, Institute of Crop Sciences, Chinese Academy of Agricultural Sciences, Beijing, China; ^2^ Hainan Yazhou Bay Seed Laboratory, Sanya, China; ^3^ Nanfan Research Institute, Chinese Academy of Agricultural Sciences, Sanya, China; ^4^ Crop Research Institute, Sichuan Academy of Agricultural Sciences, Chengdu, China

**Keywords:** maize kernel, heat response, heat tolerance, genes, pathways

## Abstract

Global warming leads to frequent extreme weather, especially the extreme heat events, which threating the safety of maize production. Here we selected a pair of maize inbred lines, PF5411-1 and LH150, with significant differences in heat tolerance at kernel development stage. The two maize inbred lines were treated with heat stress at kernel development stage. Compared with the control groups, transcriptomic analysis identified 770 common up- and down-regulated genes between PF5411-1 and LH150 under heat stress conditions, and 41 putative TFs were predicted. Based on the interaction term of the two-factorial design, we also identified 6,744 differentially regulated genes between LH150 and PF5411-1, 111 common up-regulated and 141 common down-regulated genes were overlapped with the differentially regulated genes, respectively. Combined with proteins and metabolites data, several key pathways including seven differentially regulated genes were highly correlated with the heat tolerance of maize kernels. The first is the Kyoto Encyclopedia of Genes and Genomes (KEGG) pathway ko04141: protein processing in endoplasmic reticulum, four small heat shock protein (sHSP) genes were enriched in this pathway, participating with the process of ER-associated degradation (ERAD). The second one is the myricetin biosynthesis pathway, a differentially regulated protein, flavonoid 3’,5’-hydroxylase [EC:1.14.14.81], catalyzed the synthesis of myricetin. The third one is the raffinose metabolic pathway, one differentially regulated gene encoded the raffinose synthase controlled the synthesis of raffinose, high level of raffinose enhances the heat tolerance of maize kernels. And the last one is the ethylene signaling pathway. Taken together, our work identifies many genes responded to heat stress in maize kernels, and finds out seven genes and four pathways highly correlated with heat tolerance of maize kernels.

## 1 Introduction

Maize (*Zea mays* L.) is one of the important cereal crops, its production plays an important role in world food security. Climate change is becoming more and more remarkable which has an obvious impact on crop yields all over the world, threatening food security. Climate variation accounts for roughly a third of the observed yield variability (Ray et al., 2015), the global maize yield declines with a warming climate, particularly with extreme heat events. The fifth assessment reports of IPCC pointed out that global warming is very clear. The climate change is particularly significant since 1950. From 1880 to 2012, the global temperature had increased by 0.85°C. Global warming will lead to frequent extreme weather, especially the extreme heat events. To withstand the challenges, we need climate resilience, coping with the impacts of climate change and preventing those impacts from growing worse. The only way to achieve climate resilience is cutting the heat-trapping emissions that drive climate change while adapting to the changes that are unavoidable, which means that we need mitigation and adaptation, and researchers suggested that we should pay enough attention to the risk of maize yield and take actions of mitigation and adaptation to climate change (Li et al., 2022a).

Maize originated in the seasonally dry tropical forest (Ranere et al., 2009). However, most of the maize cultivars are very sensitive to high temperature, especially at the pre-flowering, flowering, and kernel development stages. Different maize germplasms showed different ability of heat stress tolerance, previous studies showed that only a few maize germplasms have better heat resistance, such as the heat-stress tolerant line DTPYC9F119 (Singh et al., 2020), the maize cultivar DKC7221 (Doğru, 2021), and the maize heat tolerant hybrid ZD309 (Liu et al., 2022). The effects of high temperature on maize yield are different in different growth stages. High temperature will cause a marked decrease in the growth parameters at maize seedling stage (Karim et al., 2000). However, heat stress at seedling stage does not affect the yield significantly, heat stress at maize pre-flowering and flowering stage were very lethal, it will affect the plant growth, the pollen viability, and seed setting rate (BarnabÁS et al., 2008; Hedhly et al., 2009; Storme and D, 2014; Lizaso et al., 2018; Wang et al., 2020b; Yousaf et al., 2022). Heat stress at maize kernel development stage will reduce the maize yield significantly, mainly affecting grain filling and cell division. Some research showed that high night temperature dose not reduced the kernel weight (Wang et al., 2020b). Still in most cases, high temperature will reduce the kernel weight significantly. High temperature accelerates the rate of grain filling, while it was associated with shorter duration of the grain filling period, which will reduce the accumulation of dry matter of kernels significantly (Badu-Apraku et al., 1983; Lu et al., 2013; Boehlein et al., 2019). High temperature during endosperm cell division reduces kernel sink potential and subsequently mature kernel mass, mainly disrupting cell division and amyloplast biogenesis in the peripheral and central endosperm (Commuri and Jones, 1999). Although previous researchers have conducted some research on the heat tolerance of maize kernels, there is still insufficient research on the mining of heat tolerance genes and the molecular mechanisms of heat tolerance in maize kernels.

In order to cope with the effects on maize brought by high temperature and extreme high temperature weather, we should screen heat resistance maize materials, find out heat resistance related genes, and analyze the metabolic pathways correlated with heat tolerance. In recent years, although the analytical methods of bioinformatics are widely used in the study of maize under heat stress, most of them were focused on the seedling stage of maize (Hou et al., 2019; Qian et al., 2019a; Qian et al., 2019b; Zhang et al., 2019; Wang et al., 2020a; Liu et al., 2022), studies on maize kernels are rarely reported. A model-data integration researches showed that warming-induced decline in maize yield is mainly driven by direct heat stress imposed on reproductive processes, further adaptation strategies should be targeted at the heat stress during grain formation (Zhu et al., 2019). In this study, a pair of maize inbred lines, PF5411-1 and LH150, with significant differences in heat tolerance at kernel development stage were treated with heat stress. The key genes and pathways highly correlated with heat tolerance of maize kernels were identified by transcriptomic data, proteins or metabolites analysis data. Our data and analysis results can provide important information and reference for experts to conduct research on maize heat tolerance.

## 2 Materials and methods

### 2.1 Plant materials, heat treatment, and sampling

A total of 494 maize inbred lines were planted in field with normal (sowing in spring) and high temperature treatments (sowing in summer). Ear weight, kernel weight per ear, and kernel number per ear were measured, the high temperature tolerance index (HTTI) of each inbred line was calculated, HTTI is the trait value at high temperature divided by the trait value at normal temperature. The integrated HTTI value of each inbred line was calculated by principal component analysis (PCA), and the integrated HTTI value of the 494 maize inbred lines were ranked. Five high temperature tolerant lines (including PF5411-1) and five high temperature sensitive lines (including LH150) were selected, incomplete NCII experiments were carried out in Jianyang City, Sichuan province, and the general combining ability (GCA) was calculated under normal condition and heat stress condition.

The heat tolerant line PF5411-1 and heat sensitive line LH150 were further treated with heat stress during the kernel development stage. PF5411-1 and LH150 were planted in a sunlight greenhouse, and the control groups were planted beside the sunlight greenhouse in the field. The maize plants of heat stress treatment group were watered at night to eliminate drought stress. The temperature of control groups and heat treatment groups were recorded every hour by the temperature recorders (RC-4), the daily average temperature, maximum temperature, and minimum temperature of control groups and heat treatment groups during the kernel development stage were shown in **Supplementary Figure 1**. All the materials were self-pollinated on July 23, 2020 under the normal condition, heat stress was started on July 29, 2020. After 25 days treatment, the fresh ears were harvested on August 23, 2020 at noon. The temperature at the time of sampling was approximately 40°C. Three different ears from each line and each treatment were harvested, and they were frozen in liquid nitrogen immediately. All the samples were stored in -80°C refrigerator, and prepared for the extraction of RNAs, proteins, and metabolites.

### 2.2 Isolation of total RNA and quantitative real-time PCR

The samples planted in control groups were named as PF5411-1(CK) and LH150(CK), while the samples planted in heat stress treatment groups were named as PF5411-1(HT) and LH150(HT). Total RNA of maize kernels was extracted using the RNA extraction kit (Tiangen, DP432), and a total of 12 RNA samples were extracted from three biological repetitions of PF5411-1(CK), LH150(CK), PF5411-1(HT), and LH150(HT), respectively. Total RNA of the samples was reversed transcribed using the FastKing gDNA Dispelling RT SuperMix (Tiangen, KR118). The qRT-PCR was performed by using the ABI QuantStudio 3 and ChamQ Universal SYBR qPCR Master Mix (Vazyme, Q711-02). The reaction procedure is 30 s at 95°C for pre denaturation, followed by 40 cycles of amplification at 95°C for 10 s, then 60°C for 30 s, at last followed by 1 cycles of melt curve drawing at 95°C for 15 s, then 60°C for 60 s, and 95°C for 15 s. Fluorescence signal is collected in the last step of amplification and melt curve drawing. The relative expression level of the selected key genes were calculated by the 2^-ΔΔCt^ methods. The *GAPDH* gene (*Zm00001d049641*) was used as the internal control. The primers are listed in **Supplementary Table 5**.

### 2.3 Analysis of transcriptome up-regulated, down-regulated, and differentially regulated genes

The cDNA preparation was performed by the Novogene Corporation (Beijing, China). In raw sequencing data, adapter sequences and low-quality reads were removed using FASTP 0.20.1 (http://opengene.org/fastp/fastp). Clean reads were aligned to the B73 maize reference genome (B73 RefGen_V4 [released 2016], available online: https://download.maizegdb.org/Zm-B73-REFERENCE-GRAMENE-4.0/) using the HISAT2 V2.2.1. The mapped reads for each sample were assembled using StringTie (https://ccb.jhu.edu/software/stringtie/). Reads were then counted using featureCounts V2.0.1 (http://bioinf.wehi.edu.au/featureCounts/). Gene expression levels were reflected using the fragments per kilobases of transcript per million base pair sequences (FPKM) (Trapnell et al., 2010). When the whole transcriptome was done, DESeq2 (https://bioconductor.org/packages/release/bioc/html/DESeq2.html) was used to estimate the expression levels of all transcripts. The up- and down-regulated genes were filtered with |log2 (fold change)| > 1 and with statistical significance (padjBH < 0.01). Based on the interaction term of the two-factorial design, with the threshold of |log2(FoldChange)| > 1 and the adjusted P-value padjBH < 0.01. GO and KEGG enrichment were analyzed by an online tool (https://www.omicshare.com/tools/Home/Soft/getsoft). The common up- and down-regulated genes identified from the heat sensitive and resistant lines were used to predict transcription factors in the PlantTFDB data base (http://planttfdb.gao-lab.org/prediction.php). TFs were predicted by an online tool from the Plant Transcription Factor Database (http://planttfdb.gao-lab.org/prediction.php).

### 2.4 Protein identification and quantification

Kernel samples with three biological repetitions of PF5411-1(CK), LH150(CK), PF5411-1(HT), and LH150(HT) were ground into fine powder in liquid nitrogen, and the fine powder was used to extract total protein. Samples were lysed with SDT buffer (4% sodium dodecyl sulfate, 100 mM dithiothreitol, 10 mM triethylammonium formate buffer), followed by 5 minutes of ultrasonication on ice. After reacting at 95°C for 8 min, the lysate was centrifuged at 12000 g for 15 min at 4°C. And the supernatant was reduced with 10 mM dithiothreitol for 1 h at 56°C, and subsequently alkylated with sufficient iodoacetamide for 1 h at room temperature in the dark. Then samples were completely mixed with 4 times volume of precooled acetone by vortexing and incubated at -20°C for at least 2 h. Samples were then centrifuged at 12000 g for 15 min at 4°C and the precipitation was collected. After washing with 1mL cold acetone, the pellet was dissolved by dissolution buffer (8 M Urea, 100 mM triethylammonium formate buffer, pH 8.5) (Wiśniewski et al., 2009; Wu et al., 2014; Kachuk et al., 2015; Marx et al., 2016; Niu et al., 2019). The extracted proteins were used to do the Tandem Mass Tags (TMT) analysis (Ross et al., 2004). The identification and quantification of proteins was performed by the Novogene Corporation (Beijing, China). The raw files were searched in Proteome Discoverer (http://www.thermoscientific.com/en/product/proteome-discoverer-software.html) against the B73 reference genome (169673 sequences) downloaded from Uniprot (https://www.uniprot.org/). Peptide Spectrum Matches (PSM) with credibility above 99% were kept as trusted peptides or proteins, peptides or proteins with FDR greater than 1% were removed. The proteins were quantified using the total mass spectrum peak area.

### 2.5 Metabolites identification and quantification

Kernel samples with six repetitions of PF5411-1(CK), LH150(CK), PF5411-1(HT), and LH150(HT) were ground into fine powder in liquid nitrogen, and the fine powder was used to extract total metabolites. The tissues (100 mg) were resuspended with prechilled 80% methanol and 0.1% formic acid by well vortex. The samples were incubated on ice for 5 min and then were centrifuged at 15,000 g, 4°C for 20 min. Some of supernatant was diluted to final concentration containing 53% methanol by LC-MS grade water. The samples were subsequently transferred to a fresh Eppendorf tube and then were centrifuged at 15000 g, 4°C for 20 min. Finally, the supernatant was injected into the LC-MS/MS system analysis (Dunn et al., 2011; Want et al., 2013). The raw data files generated by UHPLC-MS/MS were processed using the Compound Discoverer 3.1 (CD3.1, ThermoFisher) to perform peak alignment, peak picking, and quantitation for each metabolite.

### 2.6 Determination of H_2_O_2_ content

The seedling of PF5411-1 and LH15 were treated at 42°C and 25°C for 8 hours, samples were named as PF5411-1(CK), LH150(CK), PF5411-1(HT), and LH150(HT). Samples were sent to Suzhou Keming Biotechnology Co., Ltd, and the H_2_O_2_ was tested by the company, using the ultraviolet spectrophotometry methods.

## 3 Results

### 3.1 Heat stress reduce the yield of kernel significantly

**Figure 1:**
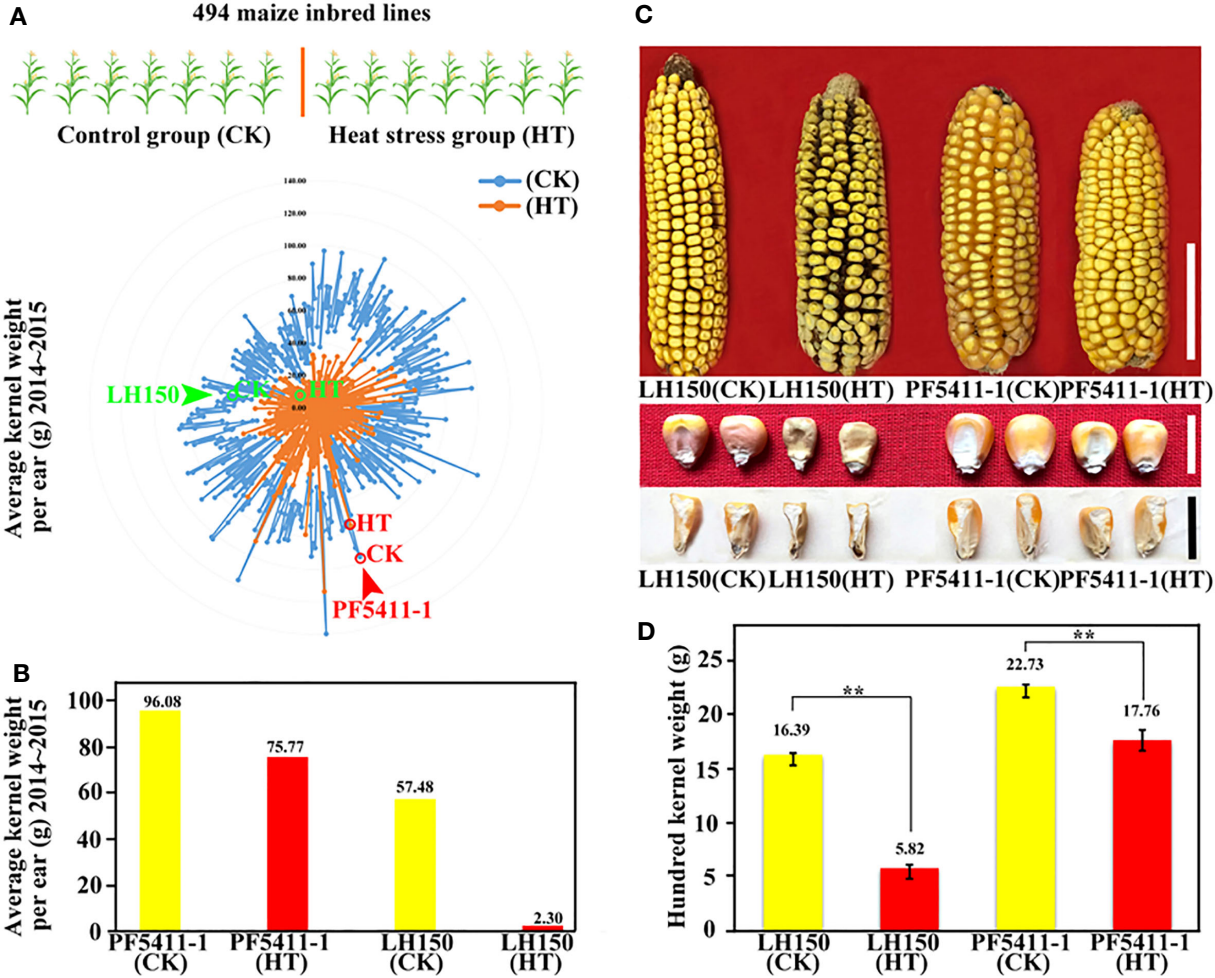
The effects of heat stress on maize kernel weight. **(A)** Radar chart of the average kernel weight of the 494 maize inbred lines. **(B)** Histogram of average kernel weight of PF5411-1 and LH150. **(C)** Ear and kernel phenotype of PF5411-1 and LH150 under normal and heat stress conditions. **(D)** Histogram of hundred kernel weight of PF5411-1 and LH150 under normal and heat stress conditions. “CK” represents control group, “HT” represents heat stress group, the same below. Scale bar for ears in C is 5 cm, and scale bar for kernels in C is 1 cm. ** indicate a significant difference between the two sets (p < 0.01), ANOVA was used to analyze significance.

LH150 and PF5411-1, selected from a 494-inbred line maize population, are two maize inbred lines with different tolerance to heat stress at kernel development stage. Two years of field experiments showed that PF5411-1 performs well under heat stress (**Figures 1A, B**). Incomplete NCII experiments showed that PF5411-1 also has a better general combining ability (GCA) than that of LH150. The GCA of PF5411-1 and LH150 at normal condition are 16.38 and 2.65, respectively. The GCA of PF5411-1 and LH150 under heat stress condition are 2.12 and -6.32, respectively (**Supplementary Table 1**). Sunlight greenhouse treatment experiment confirmed that the heat tolerance of PF5411-1 is better than that of LH150. The results showed that heat stress reduced the hundred kernel weight significantly, the hundred kernel weight of LH150 decreased by 64% after heat stress, while the hundred kernel weight of PF5411-1 only decreased by 21% (**Figures 1C, D**). The difference of heat tolerance ability between the two inbred lines is useful for further research, we can discover some genes and pathways correlated with heat tolerance of maize kernels through these two lines with significant differences.

### 3.2 Genes and transcription factors in response to heat stress

Twelve cDNA libraries of maize kernels were constructed from the total RNAs, isolated from PF5411-1(CK), LH150(CK), PF5411-1(HT), and LH150(HT), respectively. The cDNA libraries were sequenced using the Illumina HiSeq™ 2500 platform. A total of 527,622,010 raw reads were obtained, with an average GC content of 56.09%. The raw data were up loaded to the NCBI Sequence Read Archive (Accession number PRJNA957564). A total of 456,106,305 (86.45%) reads were mapped to the maize reference genome (Zm-B73-REFERENCE-NAM-4.0) (**Table 1**).

**Table 1:** Summary of reads mapping to the reference genome.

Sample and treatment	Total reads	Total map	Unique map	Multi map
LH150(CK)-1	44230292	37431801(84.63%)	35543453(80.36%)	1888348(4.27%)
LH150(CK)-2	42407962	36614404(86.34%)	34748480(81.94%)	1865924(4.4%)
LH150(CK)-3	40768114	35194653(86.33%)	33302999(81.69%)	1891654(4.64%)
PF5411-1(CK)-1	44059446	37340397(84.75%)	35676350(80.97%)	1664047(3.78%)
PF5411-1(CK)-2	46628416	39712891(85.17%)	37495705(80.41%)	2217186(4.76%)
PF5411-1(CK)-3	42121646	34772188(82.55%)	33002639(78.35%)	1769549(4.2%)
LH150(HT)-1	43667756	37950334(86.91%)	36822213(84.32%)	1128121(2.58%)
LH150(HT)-2	44739364	38822098(86.77%)	37650649(84.16%)	1171449(2.62%)
LH150(HT)-3	42417760	36809871(86.78%)	35650439(84.05%)	1159432(2.73%)
PF5411-1(HT)-1	44977948	39892443(88.69%)	38195506(84.92%)	1696937(3.77%)
PF5411-1(HT)-2	46678496	41591068(89.1%)	39543332(84.71%)	2047736(4.39%)
PF5411-1(HT)-3	44924810	39974157(88.98%)	37632998(83.77%)	2341159(5.21%)
Means	43968500	38008859(86.45%)	36272064(82.50%)	1736795(3.95%)
Sum	527622010	456106305	435264763	20841542

**Figure 2:**
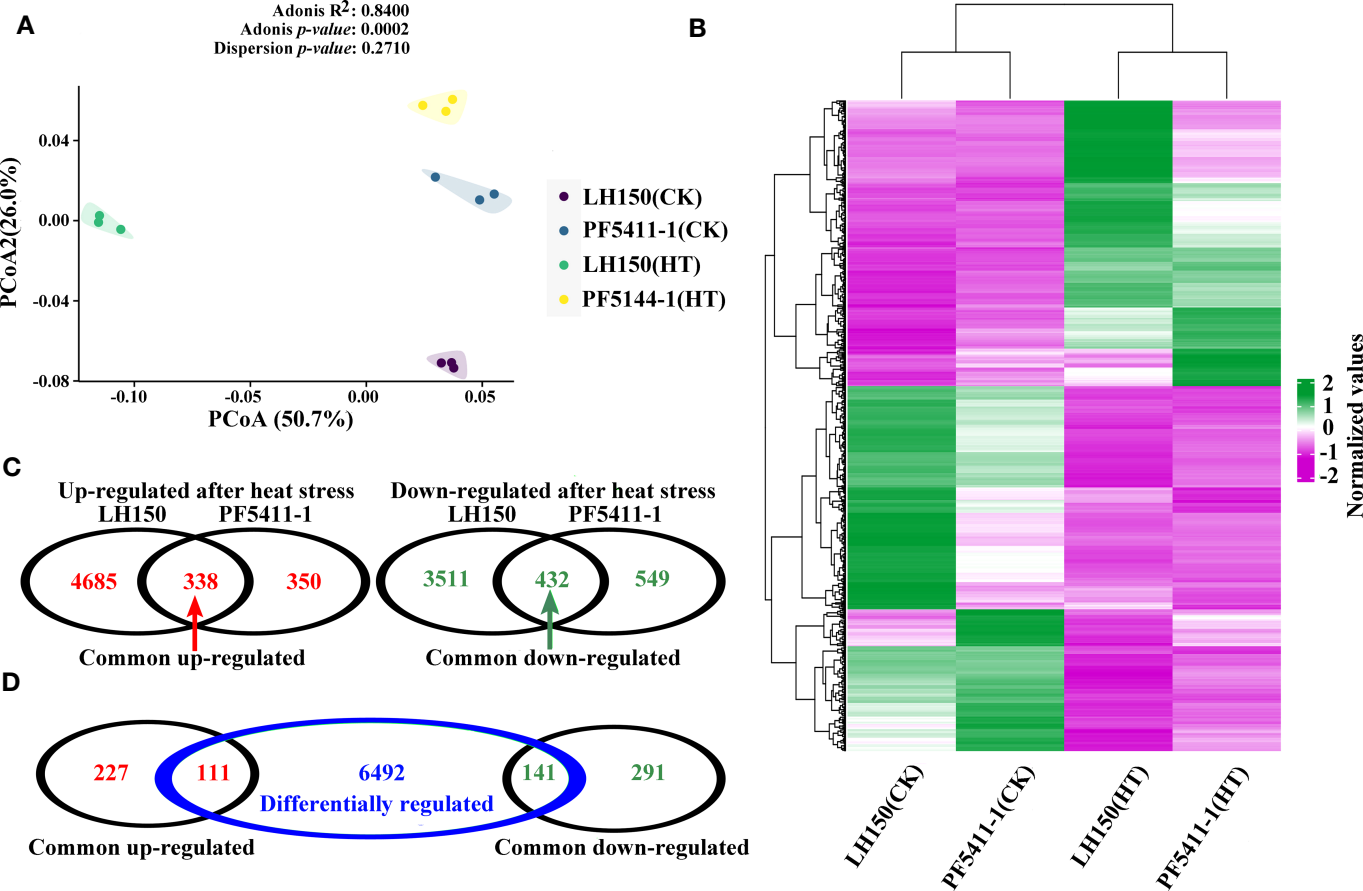
Analysis of the heat stress regulated genes in maize kernel. **(A)** Principal component analysis of the calculated FPKM value among the 12 samples. **(B)** Hierarchical clustering of the common up- and down-regulated genes. Purple and green lines in the heatmap represent the up- and down-regulated genes, respectively. **(C)** Venn diagrams illustrate the number of up-regulated and down-regulated gene sets under heat stress in LH150 and PF5411-1 or in both genotypes. **(D)** Relationship between the number of common regulated gene sets and differentially regulated gene set.

The FPKM value of 12 samples were used to do principal component analysis, and the results showed that the samples between the four groups were dispersed, while the samples within the group were gathered together (**Figure 2A**). Which indicated that the FPKM data of 12 samples were perfect for subsequent gene differential expression analysis. After heat stress, 8,966 and 1,669 up- or down-regulated genes were identified in the kernels of maize inbred line LH150 and PF5411-1, respectively (**Figure 2C**), and 6,744 genes were identified as differentially regulated genes between LH150 and PF5411-1 (**Figure 2D**). Among the heat stress regulated genes, 338 common up-regulated and 432 common down-regulated genes were identified between LH150 and PF5411-1, the details were provided in **Supplementary Table 2**. The hierarchical clustering method was employed to overview the expression pattern of each group, using the average FPKM value of the common up- and down-regulated genes (**Figure 2B**), the expression pattern of these genes showed that some differentially regulated genes were existed in the common regulated genes, these genes may play an important role in heat responses and heat tolerance. Gene comparison analysis showed that 111 common up-regulated genes were overlapped with the differentially regulated genes, and 141 common down-regulated genes were overlapped with the differentially regulated genes (**Figure 2D**). The 252 overlapped differentially regulated genes between LH150 and PF5411-1 can be used to do further analysis, in which we may discover some key genes and pathways correlated with the heat tolerance of maize kernels.

**Figure 3:**
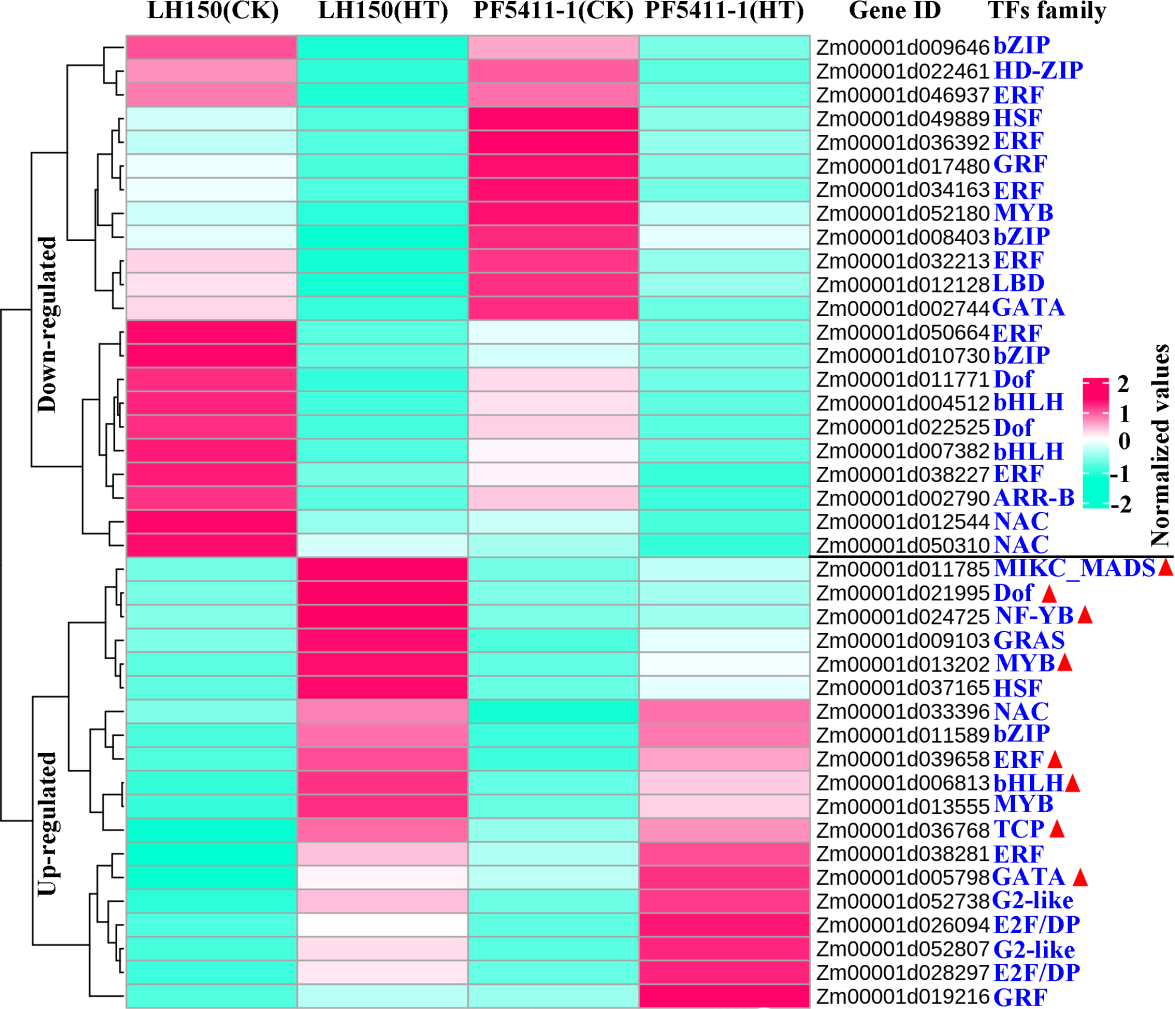
Overall expression levels of the genes encoding TFs predicted from the common up- and down-regulated genes. Rose red and bule-green lines in the heatmap represent the up- and down-regulated genes responded to heat stress, respectively. Red triangles indicate differentially up-regulated TFs of PF5411-1, responding to heat stress.

TFs are important proteins that can bind the element of genes. They ensure the target gene is expressed at a specific intensity in a specific time and space. TFs play important roles in the plants, which can regulate the plants to adapt to environmental changes. In our study, 41 putative TFs were found to respond to heat stress, which belongs to 18 TF families. There are six TF families with three or more than three TF genes in maize kernel, the largest TF family is the ERF family (**Figure 3**). Among the up-regulated TF genes, 8 of them were differentially responded to heat stress between LH150 and PF5411-1 (marked with red triangle in **Figure 3**). The differentially up-regulated TF genes may involve in the regulation of the heat tolerance of PF5411-1.

### 3.3 Gene Ontology and KEGG analysis of the heat stress response genes

**Figure 4:**
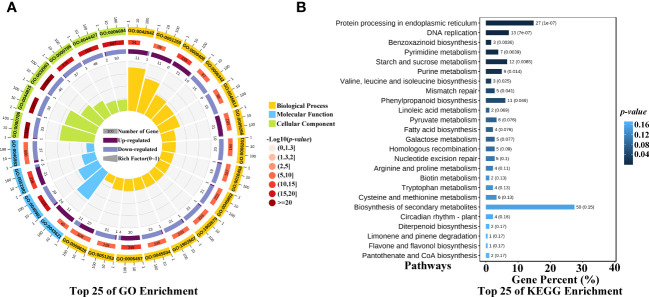
GO and KEGG enrichment of the heat stress response genes. **(A)** GO enrichment of the common up- and down-regulated genes, the function of heat stress response genes mainly involved in stress response, such as heat, temperature, hydrogen peroxide, reactive oxygen, oxygen-containing compound, oxidative, and they also related with protein folding, binding, or protein complex. **(B)** KEGG enrichment of the common up- and down-regulated genes.

To identify the significantly enriched GO terms, the 770 common up- and down-regulated genes were classified into three main GO categories, and the enriched top 25 of GO terms were showed in the circular graph of **Figure 4A**. The function of these genes responded to heat stress mainly related with the function of stress response, such as heat, temperature, hydrogen peroxide, reactive oxygen, oxygen-containing compound, oxidative, and they also related with protein folding, binding, or protein complex. To further explore the pathways associated with heat stress in maize kernels, the common up- and down-regulated genes were subjected to an online tool, and the KEGG pathways were estimated. The 770 genes were assigned to 90 KEGG pathways (**Supplementary Table 3**), and the enriched top 25 of KEGG were showed in the barplot of **Figure 4B**. The KEGG pathways in maize kernels related with the heat stress mainly involved in ko04141: Protein processing in endoplasmic reticulum, ko04016: MAPK signaling pathway-plant, ko00941: Flavonoid biosynthesis, ko00052: Galactose metabolism, and so on. The Protein processing in endoplasmic reticulum pathway may be a vital pathway involved in the heat tolerance of maize, 27 genes were assigned into this pathway (**Figure 4B**), and most of them encode small heat shock proteins (sHSPs), including the heat shock protein26, 17.4 kDa class I heat shock protein, 17.5 kDa class II heat shock protein, 22.0 kDa class IV heat shock protein, 23.6 kDa heat shock protein mitochondrial, and Class I heat shock protein 3. The results indicated that sHSPs may play an important role in heat tolerance of maize kernel.

### 3.4 Identification of the heat tolerance related genes, proteins, and metabolites in maize kernels

The 252 common up- or down-regulated genes, which overlapped with the differentially regulated genes between LH150 and PF5411-1 (**Figure 2D**), may account for the heat tolerance of maize kernels. Proteins and metabolites corresponding to the differentially regulated genes were identified and quantified by TMT and UHPLC-MS/MS, respectively. The identified protein sequences were aligned with the MaizeGDB database to obtain the corresponding gene ID (Maize B73 RefGen_v4), and the results showed that 82 out of 252 differentially regulated genes supported by the identified protein data. The protein contents of 45 differentially regulated genes were up-regulated in the heat tolerance line PF5411-1 (**Supplementary Table 4**). We speculate that these 45 differentially regulated genes are highly correlated with heat tolerance of maize kernels. In order to identify the key metabolic pathways correlated with heat tolerance of maize kernels, the 45 differentially regulated genes were assigned to the 29 KEGG pathways (**Supplementary Table 3**). Fifteen metabolites, correlated with seven differentially regulated genes, were identified from the enriched 29 KEGG pathways (**Tables 2**, **3**).

**Table 2:** Heat tolerance related key genes and their expression alterations after heat stress.

Gene ID Maize B73 RefGen_v4	Average FPKM value of LH150 (CK)	Average FPKM value of LH150 (HT)	Fold Change of LH150	Average FPKM value of PF5411-1 (CK)	Average FPKM value of PF5411-1 (HT)	Fold Change of PF5411-1
*Zm00001d035285*	4.50 ± 0.22	0.90 ± 0.36	0.20 down	2.88 ± 0.34	1.30 ± 0.10	0.45 down
*Zm00001d044728*	35.14 ± 7.47	512.67 ± 178.95	14.59 up	92.78 ± 51.87	239.65 ± 32.30	2.58 up
*Zm00001d047841*	62.56 ± 19.76	1532.35 ± 679.34	24.49 up	307.30 ± 103.25	1558.08 ± 308.23	5.07 up
*Zm00001d039566*	817.74 ± 157.42	6712.37 ± 2292.75	8.21 up	2838.58 ± 996.00	7275.93 ± 1141.72	2.56 up
*Zm00001d028408*	202.40 ± 54.71	2047.80 ± 769.94	10.12 up	449.59 ± 279.64	1358.92 ± 170.26	3.02 up
*Zm00001d047424*	8.09 ± 0.84	61.98 ± 6.69	7.66 up	15.48 ± 7.44	40.12 ± 15.57	2.59 up
*Zm00001d042541*	13.75 ± 6.11	309.36 ± 31.74	22.50 up	2.20 ± 0.42	4.64 ± 0.58	2.10 up
*Zm00001d019163*	95.65 ± 11.43	1719.79 ± 114.90	17.98 up	202.19 ± 8.24	442.30 ± 58.95	2.19 up
*Zm00001d029706*	7.68 ± 1.93	196.59 ± 3.40	25.61 up	17.28 ± 3.47	46.89 ± 13.30	2.71 up
*Zm00001d051161*	6.35 ± 2.31	100.25 ± 17.90	15.80 up	14.19 ± 4.29	39.33 ± 23.08	2.77 up
*Zm00001d003164*	26.99 ± 2.93	1.51 ± 0.21	0.06 down	9.15 ± 1.21	4.40 ± 0.53	0.48 down
*Zm00001d051161*	6.35 ± 2.31	100.25 ± 17.90	15.80 up	14.19 ± 4.29	39.33 ± 23.08	2.77 up

**Table 3:** Heat tolerance related key genes and the expression alterations of their corresponding coding protein after heat stress.

Gene ID Maize B73 RefGen_v4	Protein ID UniProt	Average Protein quantification value of LH150 (CK)	Average Protein quantification value of LH150 (HT)	Fold Change of LH150	Average Protein quantification value of PF5411-1 (CK)	Average Protein quantification value of PF5411-1 (HT)	Fold Change of PF5411-1	Related Metabolites
*Zm00001d035285*	A0A1D6LFA6	281.83 ± 31.02	325.00 ± 56.29	1.15 up	236.83 ± 10.62	276.23 ± 43.72	1.17 up	None
*Zm00001d044728*	B6TQD6	412.53 ± 18.67	922.03 ± 49.18	2.24 up	457.87 ± 20.93	1167.57 ± 51.62	2.55 up	None
*Zm00001d047841*	A0A3L6DEF1	971.70 ± 109.46	3352.43 ± 224.23	3.45 up	1059.83 ± 53.64	3218.53 ± 171.68	3.04 up	None
*Zm00001d039566*	B4F9K4	1251.63 ± 29.98	1949.33 ± 150.74	1.56 up	1620.47 ± 137.10	3489.50 ± 172.04	2.15 up	None
*Zm00001d028408*	B7ZEQ0	7236.53 ± 187.28	24858.17 ± 1534.00	3.44 up	6756.00 ± 576.87	18828.73 ± 1449.86	2.79 up	None
*Zm00001d047424*	A0A3L6DA02	527.17 ± 10.13	493.83 ± 14.52	0.94 down	562.27 ± 26.06	809.70 ± 155.57	1.44 up	Dihydrokaempferol, Dihydromyricetin, Kaempferol, Quercetin, Myricetin
*Zm00001d042541*	A0A3L6FBM0	630.23 ± 14.91	2983.23 ± 320.87	4.73 up	510.00 ± 42.01	527.90 ± 38.40	1.04 up	(±)9-HpODE
Zm00001d019163	B6SRV6	143.10 ± 27.40	196.27 ± 4.75	1.37 up	157.27 ± 7.48	223.33 ± 38.59	1.42 up	Galactinol, Raffinose
*Zm00001d029706*	B6SS87	809.07 ± 58.51	1961.17 ± 47.95	2.42 up	871.50 ± 60.74	1277.23 ± 420.77	1.47 up	L-Glutathione
*Zm00001d05116*1	A0A3L6F5U1	240.17 ± 25.48	332.17 ± 26.52	1.38 up	292.10 ± 14.03	374.37 ± 168.47	1.28 up	D-Phenylalanine
*Zm00001d003164*	B6TLH5	103.47 ± 36.13	154.63 ± 26.33	1.49 up	80.30 ± 9.77	94.80 ± 8.79	1.18 up	UDP, dUDP, CDP
*Zm00001d051161*	A0A3L6F5U1	240.17 ± 25.48	332.17 ± 26.52	1.38 up	292.10 ± 14.03	374.37 ± 168.47	1.28 up	p-Coumaric acid, Cinnamic acid

**Figure 5:**
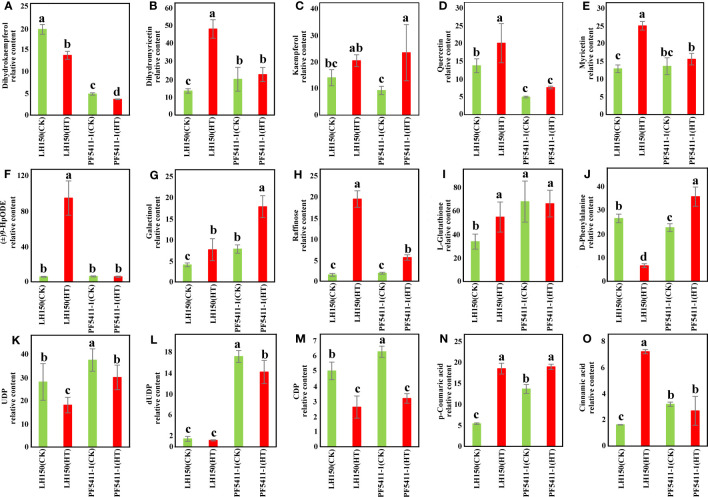
Histogram of the relative contents of metabolites related with heat resistance identified in maize kernels. **(A-O)** Showed the fifteen metabolites (listed in **Table 3**) and their relative content. The error lines are the standard deviation, and the letters above the error lines indicate significant difference (p < 0.05) among different groups, which were calculated using the LSD method in multiple comparisons.

After heat stress, there is a significant difference in the accumulation of metabolites in LH150 and PF5411-1. Dihydrokaempferol, dihydromyricetin, kaempferol, quercetin, and myricetin were the metabolites involved in the flavonoid biosynthesis pathway, these flavonoids are antioxidants in plants, which can eliminate some reactive oxygen species (ROS) produced by stress in plants. The content of dihydrokaempferol was reduced significantly in LH150 and PF5411-1 (**Figure 5A**), and the contents of dihydromyricetin, kaempferol, quercetin, and myricetin were all increased in LH150 and PF5411-1. Compared with PF5411-1, LH150 has a greater degree of variation of these flavonoids (**Figures 5B–E**), which indicated that heat sensitive line needs more antioxidants to eliminate the accumulated ROS, or the clearing pathways of ROS were inhibited. In the galactose metabolism pathway, the galactinol and raffinose contents were increase significantly under heat stress condition (**Figures 5G, H**). Metabolite content of (±)9-HpODE was significantly increased in LH150 after heat stress, while the heat tolerance line PF5411-1 did not have any changes (**Figure 5F**), which means that the metabolites of linoleic acid may participate in the response of heat stress. The contents of amino acids metabolites, L-Glutathione and D-Phenylalanine, were significantly increased or reduced in LH150 and PF5411-1 (**Figures 5I, J**), which means that amino acids metabolism is also affected by heat stress. The metabolites contents of pyrimidine metabolism were all reduced by heat stress, such as UDP, dUDP, and CDP (**Figures 5K–M**). The content of p-coumaric acid was significantly increased in LH150 and PF5411-1 (**Figure 5N**), and the cinnamic acid content was only significantly increased in LH150 (**Figure 5O**), p-coumaric acid and cinnamic acid were involved in the phenylpropanoid biosynthesis pathway. Taken together, our results indicated that the myricetin and raffinose are important metabolites for heat tolerance in maize kernels.

### 3.5 Key pathways highly correlated with the heat tolerance of maize kernels

**Figure 6:**
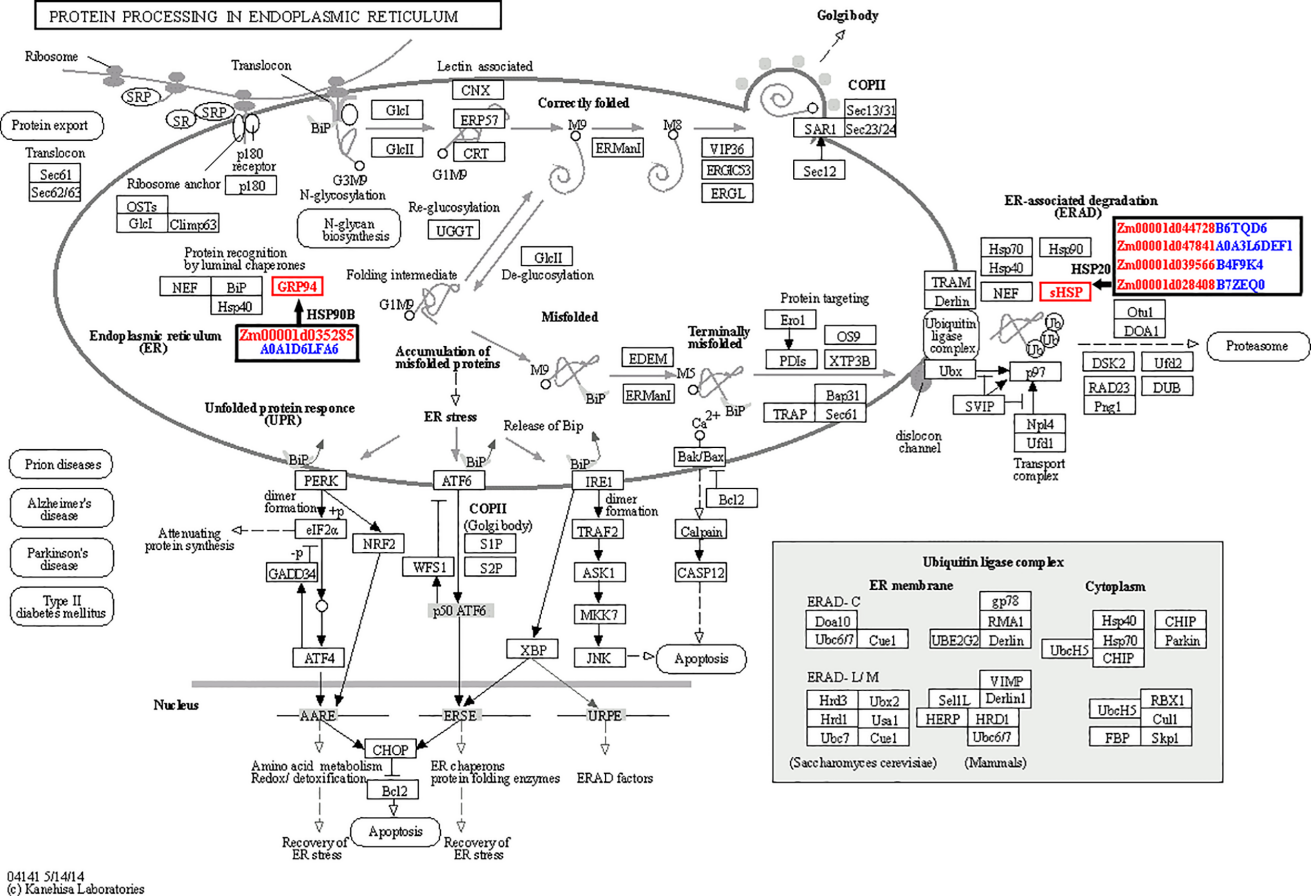
Sketch map of the protein processing in endoplasmic reticulum pathway. The figure was drawn by an online tool, OmicShare. The ID of differentially regulated genes were marked with red letters in the black boxes, and the corresponding protein IDs were marked with blue letters.

According to the knowledge of previous studies related with heat stress in plants and our results, we inferred that several pathways were highly correlated with heat tolerance of maize kernels. The first one is the protein processing in endoplasmic reticulum pathway. In plants, HSPs will express in large amounts after being stimulated by stress such as heat stress. They are important components of plant adaptation to heat stress, and play a significant role in alleviating the damage caused by heat stress. Here we identified five HSPs, highly correlated with heat tolerance of maize kernels. Among the five differentially regulated HSP genes, one was down-regulated in LH150 and PF5411-1 (HSP90B gene), and the remaining four were significantly up-regulated in both lines (sHSP genes). Interestingly, PF5411-1 has much higher sHSP gene expression levels than that of LH150 under normal conditions. After heat stress, the gene expression alteration range of LH150 is much higher than that of PF5411-1 (**Table 2**). Protein identification results showed that HSP90B and four sHSPs were all up-regulated after heat stress, the trend of protein expression alteration is consistent with the trend of gene expression alteration, except for the HSP90B protein (**Table 2**). The expression analysis of these sHSP genes indicated that they played an important role in the heat tolerance of maize kernels. KEGG enrichment results showed that the four sHSP genes participate in the ER-associated degradation (ERAD) process, and the HSP90B gene participates in the protein recognition process (**Figure 6**). Taken together, these results indicated that the sHSP genes (*Zm00001d044728*, *Zm00001d047841*, *Zm00001d039566*, and *Zm00001d028408*) were highly associated with the heat tolerance of maize kernels.

**Figure 7:**
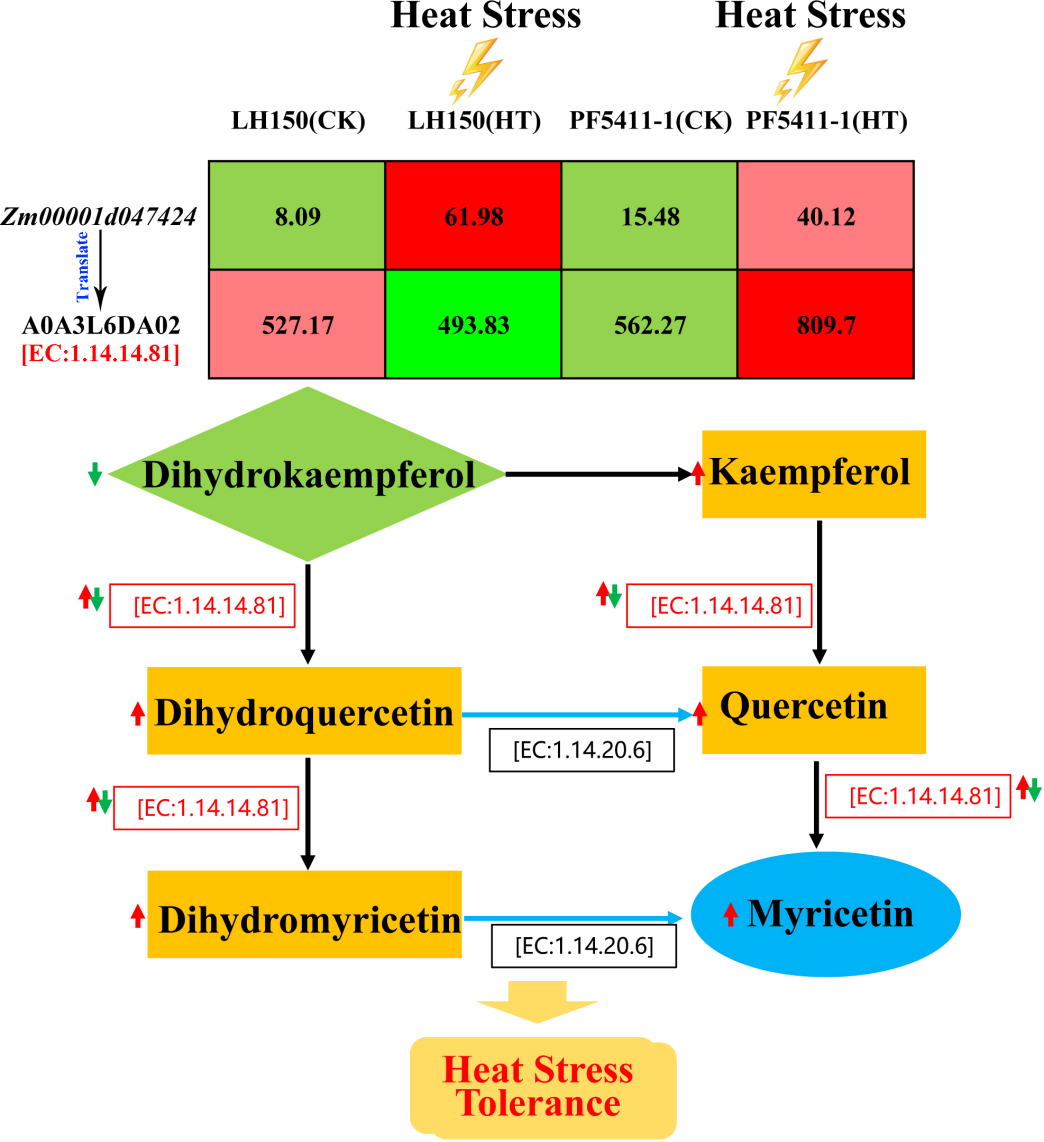
Sketch map of the synthesis of myricetin. Red arrows indicate up-regulated protein and metabolites after heat stress, and green ones indicate the down-regulated protein. Values in the table represent the expression levels of *Zm00001d047424* gene and its protein.

The second one is the flavone and flavonol biosynthesis pathway. A differentially regulated gene, *Zm00001d047424* (**Table 2**), is participated in this metabolic pathway. Curiously, the gene expression of LH150 was up-regulated 7.66-fold after heat stress, while protein expression actually decreased. Compared with LH150, the gene and protein expression levels of PF5411-1 were all up-regulated significantly (**Table 3**). *Zm00001d047424* encodes flavonoid 3’,5’-hydroxylase [EC:1.14.14.81], it participates in several steps of the biosynthesis of myricetin (**Figure 7**). Dihydrokaempferol is the substrate of dihydromyricetin, kaempferol, quercetin, and myricetin, its relative content significantly reduced by heat stress in LH150 and PF5411-1, which indicated that it was extensively decomposed to synthesize downstream products. Dihydromyricetin, kaempferol, and quercetin were finally decomposed to synthesize myricetin. Myricetin is an antioxidant, which can eliminate ROS, high level of myricetin indicated that LH150 need more myricetin to eliminate ROS (**Figure 5E**). Taken together, these results indicated that the flavonoid 3’,5’-hydroxylase gene (*Zm00001d047424*) is highly correlated with the heat tolerance of maize kernels.

**Figure 8:**
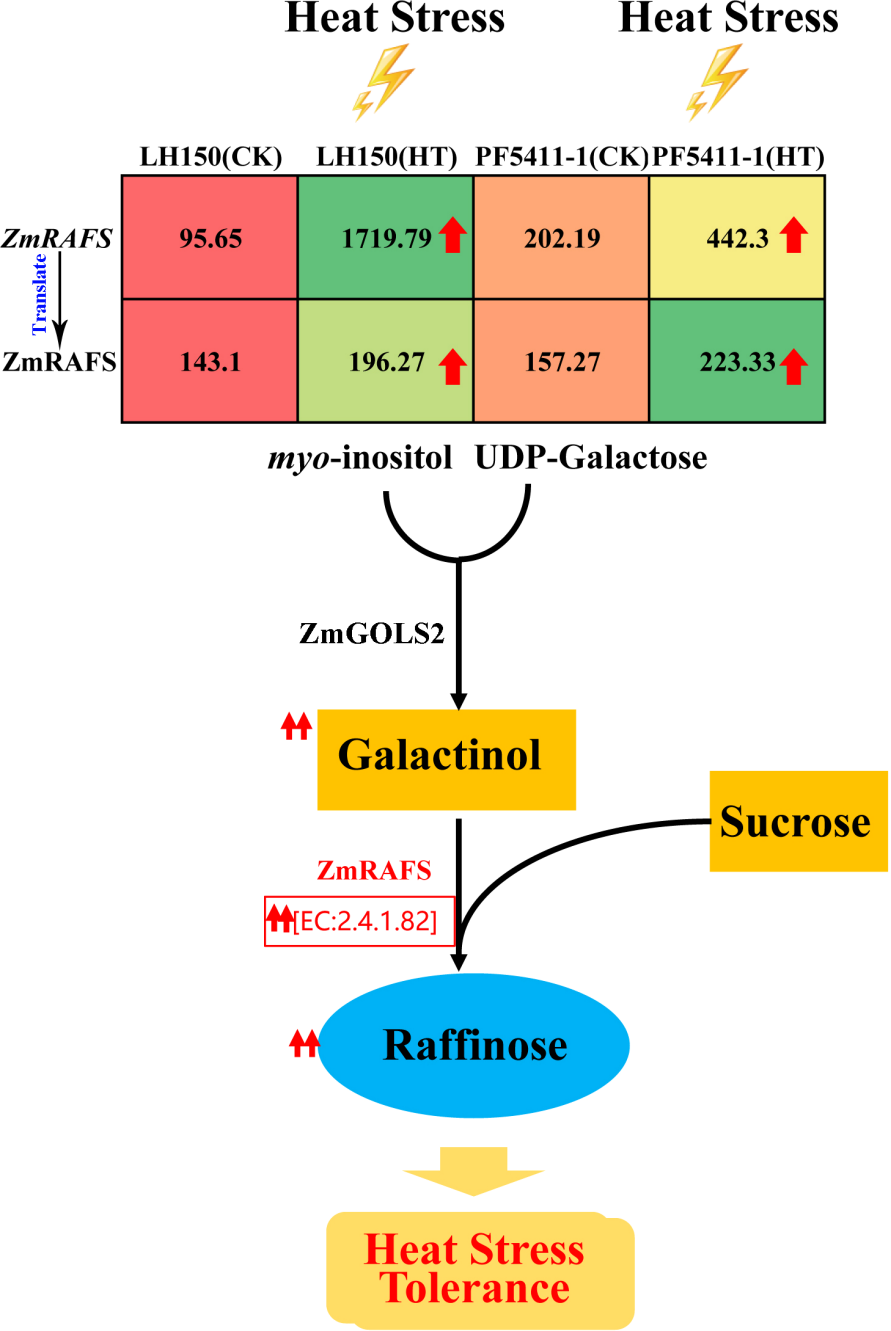
Sketch map of the synthesis of raffinose. Red arrows indicate up-regulated protein and metabolites after heat stress. Values in the table represent the expression levels of *ZmRAFS* gene and its protein.

The third one is the galactose metabolism pathway. Raffinose synthase [EC:2.4.1.82] was encoded by a differentially up-regulated gene, *Zm00001d019163* gene. Under normal conditions, the expression level of raffinose synthase gene in PF5411-1 is twice that of LH150 (**Table 2**), and the content of raffinose synthase is higher in PF5411-1 (**Table 3**). After heat stress, the expression level of raffinose synthase gene increased by more than 17 times in LH150, which means that the heat sensitive line needs more raffinose to cope with heat stress damages. And the metabolites quantification results confirmed our speculation (**Figure 5H**). Previous studies thought that raffinose plays an important role in plant tolerance of abiotic stress, and the synthesis of raffinose modulates the heats tolerance in Arabidopsis (Gu et al., 2019). Heat stress can cause an increase in the expression of genes involved in raffinose biosynthesis, and finally increase the content of raffinose (**Figure 8**). Our results indicated that raffinose plays an important role in the heat tolerance of maize kernels. The function of the raffinose synthase gene (*Zm00001d019163*) need to do further research to confirm its role in the heat tolerance of maize kernels.

In order to validate the gene expression data obtained by the transcriptome sequencing analysis, the seven key genes highly related with heat tolerance of maize kernels were selected for qRT-PCR. Relative expression levels of each gene were calculated with the 2^-ΔΔCt^ methods, the expression levels of LH150(CK) were set as 1.00 in each histogram and the results were consistent with that of transcriptome sequencing analysis (**Supplementary Figure 2**), showed similar patterns. Thus, the comparisons of data from qRT-PCR and transcriptome sequencing analysis fully validated the results from our transcriptome study.

## 4 Discussion

Climate change is becoming more and more remarkable which has an obvious impact on crop yields all over the world, the global maize yield declines with a warming climate, particularly with extreme heat events. In order to adapt the extreme heat events, researcher suggested that different optimal solution should be considered to maintain maize production and reduce the risk of heat stress under the changing climate (Deihimfard et al., 2022). There are many ways to deal with the threat of high temperature during the maize production. The first way is optimizing the cultivation methods, adjusting the sowing date to avoid the high temperature during the maize key development stages, and reasonable irrigation can help maize cool down when high temperature happened. The second way is to select heat resistant germplasm and use traditional breeding methods, such as backcross, to improve heat tolerance maize. The third way is mapping or cloning the high temperature tolerance genes and loci, and introgressing these gene in heat sensitive lines to improve the heat tolerance of maize. The best way is the second one or the third one, we can select the heat resistant germplasm and discover the heat tolerance genes, and then breeders can use them in heat tolerance maize breeding. In this study, we selected a pair of maize inbred lines form a natural population (**Figure 1**). The kernel weight of these lines decreased significantly after heat treatment, but PF5411-1 was more resistant than LH150. This indicated that PF5411-1 possessed some unique genes or regulation mechanisms to cope with heat stress.

Different from animals, plants cannot move to avoid all kinds of biological and abiotic stresses. However, plants can develop a remarkable adaptability to environments, which relied on a large repertoire of regulatory mechanisms that orchestrates transcriptional reprogramming and adaptation (Nakashima et al., 2009). When heat stress occurred, plants will change the expression level of some particular genes and proteins, finding out these key genes responded to heat stress is useful for us to create heat tolerant plants. Until now, researchers have confirmed that many genes can improve the heat tolerance of plants, such as, *AtGRXS1* (Sprague et al., 2022), *ZmHUG1* (Xie et al., 2022), *WPGD1*, *WPGD2* (Ribeiro et al., 2020), *Cat1*, *Cat2*, *Cat3* (Scandalios et al., 2000), *ZmDHAR*, and *ZmADCS* (Xiang et al., 2020). In addition, GWAS and QTL analysis were also used to find out some useful loci, coping with heat stress (Ottaviano et al., 1991; Frova and Sari-Gorla, 1994; Gao et al., 2019; Zeng et al., 2020; Seetharam et al., 2021). TFs can ensure the expression of target gene at a specific intensity in a specific time and space. Some maize TFs were proved to be correlated with heat tolerance, such as *ZmHsf04* gene (Jiang et al., 2017), *ZmHsf05* gene (Si et al., 2021), *ZmWRKY106* gene (Wang et al., 2018), and *ZmNAC074* (Xi et al., 2022). We predicted 41 putative TFs form the common up- and down-regulated genes, they belong to 18 TF families, including the HSF and NAC TF family, the largest one is the ERF family. Among the common up- and down-regulated TFs, the differentially up-regulated TFs may account for the heat tolerance of PF5411-1, they were marked with red triangles in **Figure 3**. The differentially up-regulated TFs can be further analyzed, they may useful in improving the ability of maize to withstand high temperatures.

HSPs include HSP100, HSP90, HSP70, HSP60, and HSP20 (sHSPs), HSP20 includes the 12-25 kDa molecular weight polypeptides (Waters, 2013; Waters and Vierling, 2020; Qi et al., 2022). HSPs can enhance the heat tolerance of maize, such as the HSP101 protein (Li et al., 2022b). In our study, many HSP genes were identified in the common up- and down-regulated genes, and most of them were sHSPs. All the sHSPs were enriched in the protein processing in endoplasmic reticulum pathway (**Figure 4**), the sHSPs were mainly involved in the ERAD process. Our study showed that the heat tolerance line has much higher sHSPs levels than that of heat sensitive line under normal conditions. After heat stress, the heat sensitive line needs more sHSPs to cope with heat stress damages, and the sHSP genes were up-regulated significantly, about 8 to 24 times of fold changes (**Table 2**). Hsp101 plays key role in the regulation of heat stress tolerance in rice (Kumar et al., 2023b). Unlike high molecular weight Hsp101, our results indicated that sHSPs are key proteins in dealing with heat stress in maize kernels. Research in rice showed that most of the sHSP genes are highly upregulated in response to high temperature, they may be involved in cellular functions under non-stress and stress conditions as well as during developmental processes (Sarkar et al., 2009). The increasement of sHSPs was also identified in maize seedling under heat stress condition by other researchers (Liu et al., 2022; Gao et al., 2023). The generation of reactive oxygen species (ROS) can induce sHSP and HSP101 synthesis (Kotak et al., 2007), sHSP and HSP101 play important role in heat tolerance in plants and bacterial cells (Kumar et al., 2015; Tiwari et al., 2021; Kumar et al., 2023a), high levels expression of sHSPs indicated that more ROS were generated in the heat sensitive line, stimulating the expression of sHSP genes and sHSPs (**Figure 6**). H_2_O_2_ is a kind of ROS, we determined the H_2_O_2_ content of two lines under normal condition and heat stress condition. The results showed that heat sensitive line LH150 has much lower H_2_O_2_ content than that of heat tolerance line PF5411-1, and heat stress increased the H_2_O_2_ content of LH150 and PF5411-1, while LH150 has much higher H_2_O_2_ content than that of PH5411-1 (**Supplementary Figure 3**). Compared with the expression data of sHSPs in **Table 2**, we inferred that ROS can stimulate the expression of sHSPs. However, more molecular level evidence is needed to prove it. The four differentially up-regulated sHSP genes (*Zm00001d028408*, *Zm00001d047841*, *Zm00001d039566*, and *Zm00001d044728*) identified in our research may the key genes account for the heat tolerance of maize kernels. Overexpression of the sHSP genes in maize may increase the heat tolerance of maize kernels, and further analysis needed to confirm their role in heat tolerance of maize.

Heat stress will cause a sharp increase in ROS contents, this may lead serious damage to the cell structure, especially the membrane structure (Belhadj Slimen et al., 2014). In order to adapt to heat stress, plants activate a series of mechanisms to form antioxidants and eliminate excessive ROS. Heat stress triggers the ethylene signaling pathway, the ethylene will stimulate the synthesis of antioxidants, protecting plants from oxidative damage (Kotak et al., 2007). In our study, a differentially up-regulated gene in MAPK signaling pathway-plant was identified (*Zm00001d027524*, CHIB), it involved in the ethylene defense responses process, and it may relate with the increasement of antioxidants in maize kernels (dihydromyricetin and myricetin). The dihydromyricetin and myricetin contents of heat tolerance line were much higher than that of heat sensitive line under normal condition, after heat stress the dihydromyricetin and myricetin content was increased significantly, especially the heat sensitive line (**Figures 5B, E**). The increasement of dihydromyricetin and myricetin in maize kernels under heat stress conditions indicated that the two metabolites can mediate the heat stress tolerance.

Raffinose can enhance plants adaptation to abiotic stress, and it has been proven that raffinose can enhance the tolerance of plants to heat stress (Gu et al., 2019; Li et al., 2020). In our study, we found that the gene, encoding raffinose synthase, was differentially up-regulated (**Table 2**), and the content of raffinose and its synthetic substrate galactinol were significantly increased in maize kernels after heat stress (**Figures 5G, H**). Therefore, we inferred that raffinose and galactinol can improve the heat tolerance of maize kernels, and the genes encoding the enzymes involved in the synthesis of raffinose and galactinol maybe useful in heat tolerance of maize kernels (**Figure 8**). Therefore, the genes involved in the raffinose synthesis pathway can be used to improve the heat tolerance of maize in breeding.

## 5 Conclusion

In conclusion, heat stress will induce the damage of maize kernels. In order to adapt heat stress, the expression levels of thousands of genes were changed to rescue the damages. Our research indicated that sHSPs genes (*Zm00001d028408*, *Zm00001d047841*, *Zm00001d039566*, and *Zm00001d044728*) play important role in heat tolerance of maize kernels. In addition, the genes involved in myricetin biosynthesis (*Zm00001d047424*) and raffinose biosynthesis (*Zm00001d019163*) are highly correlated with heat tolerance of maize kernels. Furthermore, a differentially up-regulated gene (*Zm00001d027524*) in the ethylene signaling pathway may relate with heat tolerance of maize kernels.

## Data availability statement

The datasets presented in this study can be found in online repositories. The names of the repository/repositories and accession number(s) can be found below: https://www.ncbi.nlm.nih.gov/, https://www.ncbi.nlm.nih.gov/sra/PRJNA957564.

## Author contributions

YC performed the experiments, prepared figures, and prepared the manuscript. TD analyzed the transcriptomic data, prepared the draft of the manuscript. JZ analyzed the proteins and metabolites, checked the manuscript. SC analyzed the transcriptomic data, checked the manuscript. JF Guided data analysis. HL Guided data analysis, prepared the draft of the manuscript, and checked the manuscript. QY performed the experiments, provided maize inbred lines, and guided data analysis. All authors contributed to the article and approved the submitted version.
