# Supplementary material for: Genes and pathways correlated with heat stress responses and heat tolerance in maize kernels
Source: Front Plant Sci. 2023 Aug 17;14:1228213. doi: 10.3389/fpls.2023.1228213 (PMC10470023; doi:10.3389/fpls.2023.1228213)
Supplement: Supplementary file 1 [file DataSheet_1.docx]

**Genes and pathways correlated with heat stress responses and heat tolerance in maize kernels**

**Supplementary materials**


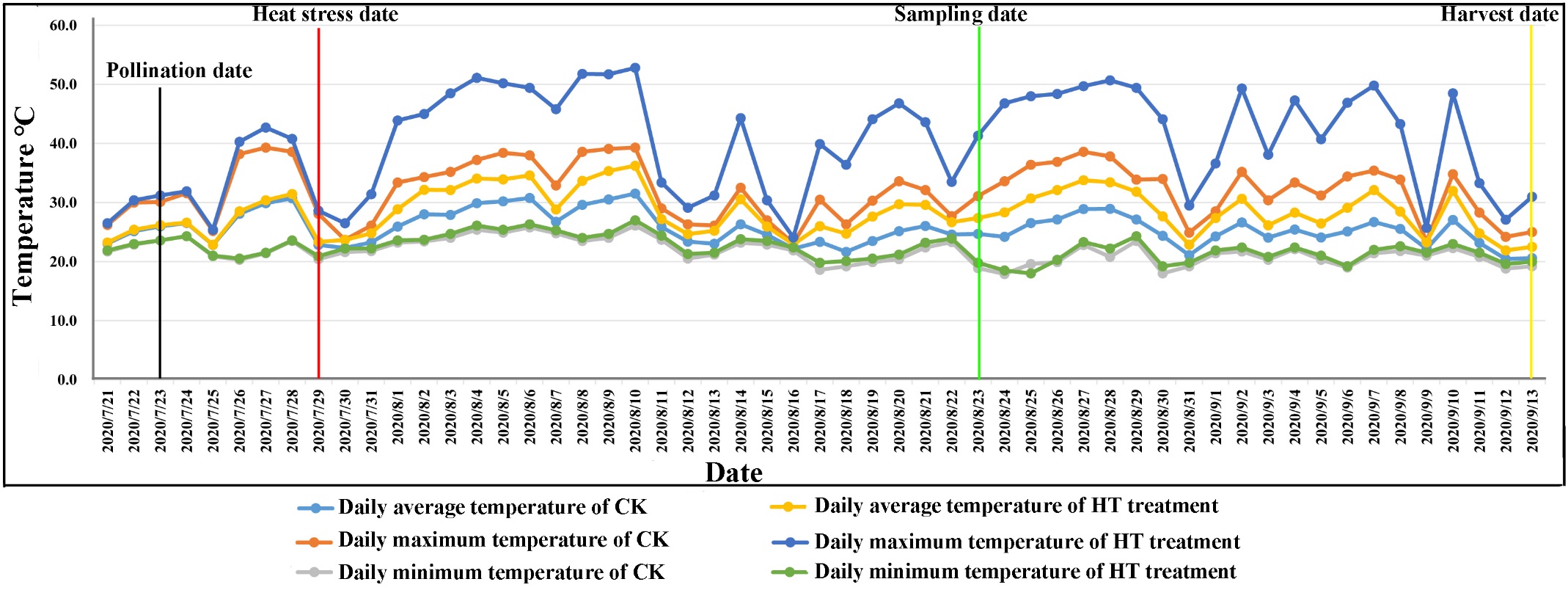


**Supplementary Figure 1** Line chart of temperature change between control group CK and HT heat stress treatment.


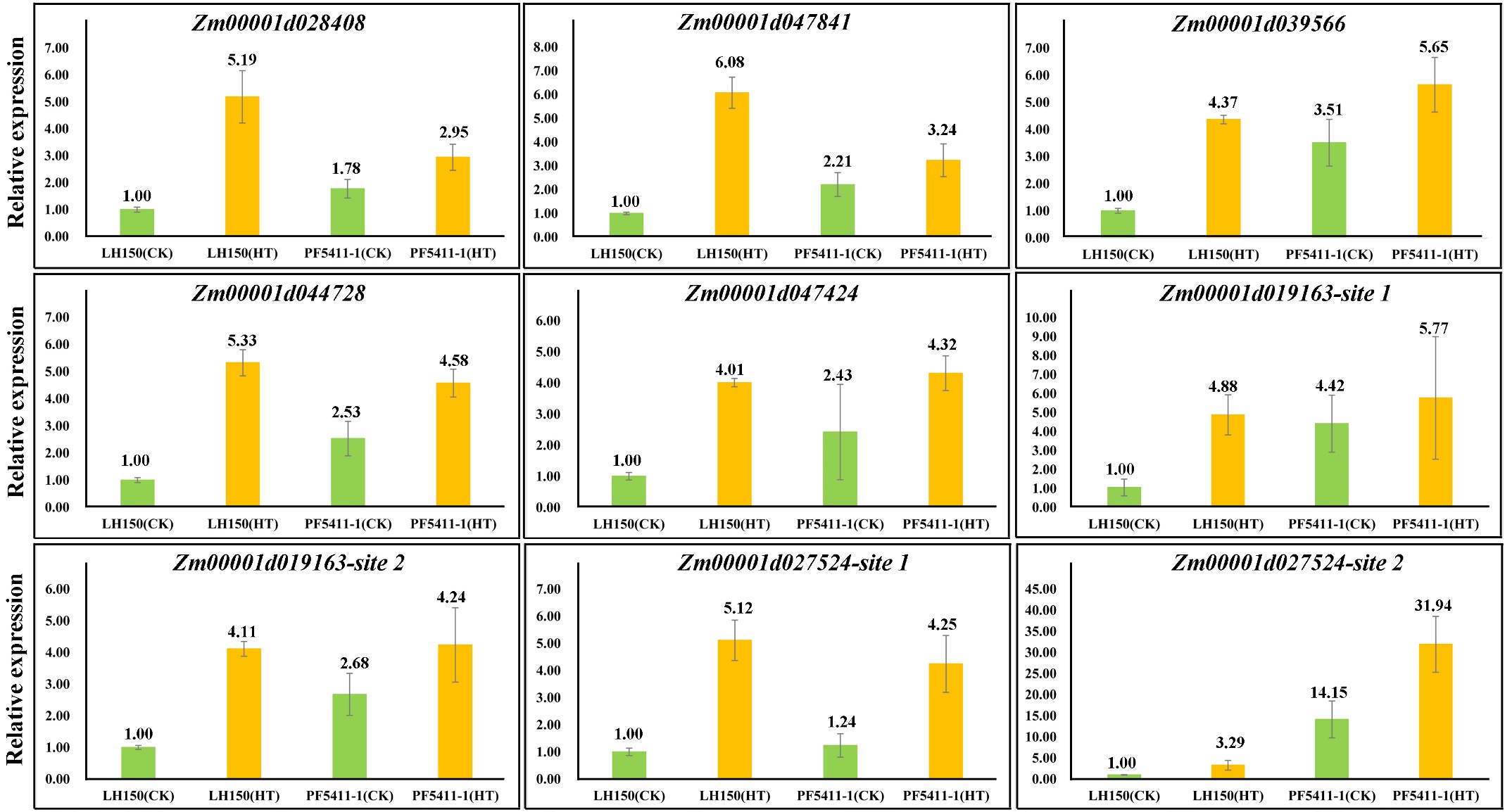


**Supplementary Figure 2** Confirmation of the expression levels of seven heat tolerance related genes by qRT-PCR. Bars represent the standard error of three replications.

**
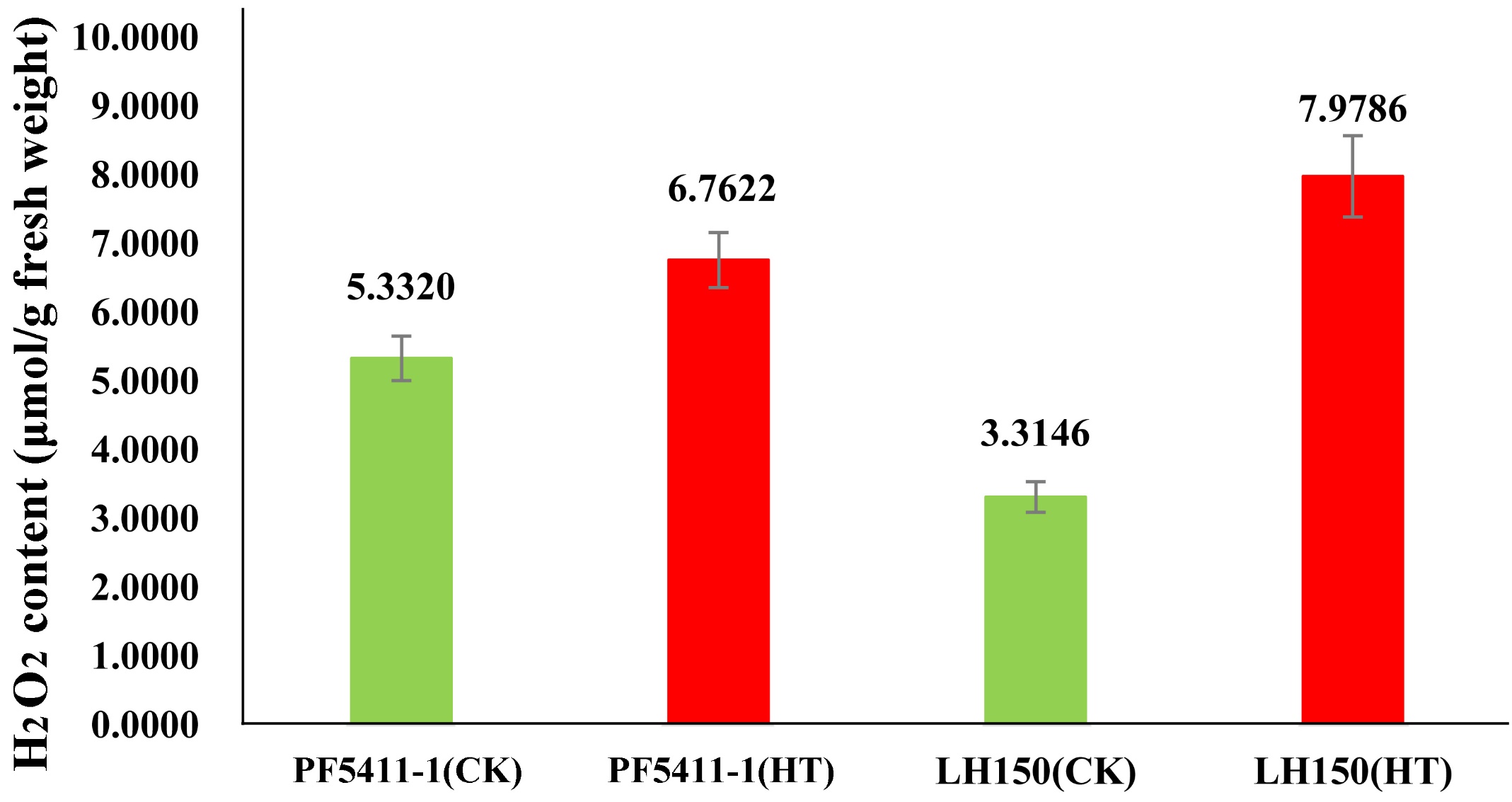
**

**Supplementary Figure 3** H_2_O_2_ content in seedling of LH150 and PF5411-1 after heat treatment.

**Supplementary Table 1** General combining ability of the five heat resistant lines and heat sensitive lines.

| Maize inbred lines | GCA of normal condition | GCA of heat stress | Note |
| --- | --- | --- | --- |
| ML1121 | 3.98 | 19.94 | Heat resistant line |
| Y1127 | 7.84 | 13.75 | Heat resistant line |
| CR14 | -22.41 | -34.81 | Heat resistant line |
| CLWN240 | -5.79 | -1.00 | Heat resistant line |
| PF5411-1 | 16.38 | 2.12 | Heat resistant line |
| CLWN247 | 4.01 | 3.92 | Heat sensitive line |
| CLWN248 | 4.06 | 27.02 | Heat sensitive line |
| LH150 | 2.65 | -6.32 | Heat sensitive line |
| CLWN256 | 3.42 | -12.11 | Heat sensitive line |
| Lp215D | -14.14 | -12.50 | Heat sensitive line |

**Supplementary Table 2** Detail information of the 770 common up- and down-regulated genes between LH150 and PF5411-1.

| GeneID_V4 | LH150(CK)_FPKM | LH150(HT)_FPKM | PF5411-1(CK)_FPKM | PF5411-1(HT)_FPKM | up- or down-regulated | gene_description |
| --- | --- | --- | --- | --- | --- | --- |
| Zm00001d048592 | 0.00 | 5.28 | 0.00 | 0.48 | UP | RUBISCO activase1 |
| Zm00001d000556 | 0.11 | 78.96 | 0.21 | 3.91 | UP | - |
| Zm00001d031711 | 0.01 | 1.58 | 0.00 | 0.21 | UP | Disease resistance gene analog PIC15 |
| Zm00001d007271 | 3.34 | 483.77 | 4.60 | 80.20 | UP | 22.0 kDa class IV heat shock protein |
| Zm00001d035214 | 0.38 | 53.58 | 0.27 | 2.30 | UP | Ureide permease 5 |
| Zm00001d052190 | 0.58 | 79.68 | 0.79 | 3.70 | UP | - |
| Zm00001d047541 | 0.05 | 5.71 | 2.45 | 18.03 | UP | - |
| Zm00001d047664 | 0.72 | 74.22 | 1.95 | 11.40 | UP | Multidrug resistance protein ABC transporter family protein |
| Zm00001d009568 | 0.98 | 75.95 | 3.37 | 10.99 | UP | Glutaredoxin-C9 |
| Zm00001d023994 | 0.24 | 16.72 | 0.47 | 6.40 | UP | Beta-glucosidase%2C chloroplastic |
| Zm00001d030712 | 0.13 | 8.65 | 1.76 | 13.90 | UP | Pectinesterase |
| Zm00001d039094 | 0.72 | 46.02 | 0.41 | 1.58 | UP | 3-ketoacyl-CoA synthase |
| Zm00001d013406 | 0.30 | 19.16 | 2.46 | 6.33 | UP | alpha/beta-Hydrolases superfamily protein |
| Zm00001d004243 | 13.89 | 858.94 | 31.17 | 323.20 | UP | Peptidyl-prolyl cis-trans isomerase FKBP65 |
| Zm00001d037023 | 0.16 | 8.80 | 0.93 | 8.59 | UP | Putative RING zinc finger domain superfamily protein |
| Zm00001d020631 | 0.80 | 38.35 | 1.18 | 4.33 | UP | root-specific kinase 1 |
| Zm00001d015700 | 0.22 | 10.25 | 0.19 | 1.32 | UP | Putative chloride channel-like protein CLC-g |
| Zm00001d013264 | 0.34 | 14.47 | 0.65 | 3.50 | UP | - |
| Zm00001d021995 | 8.86 | 375.15 | 17.68 | 50.08 | UP | LOB domain-containing protein 38 |
| Zm00001d029814 | 0.47 | 17.21 | 0.29 | 1.72 | UP | Xyloglucan endotransglucosylase/hydrolase protein 32 |
| Zm00001d024725 | 0.30 | 10.04 | 0.17 | 1.03 | UP | Putative MYB DNA-binding domain superfamily protein |
| Zm00001d050417 | 0.02 | 0.64 | 0.00 | 0.65 | UP | Cinnamoyl-CoA reductase 1 |
| Zm00001d015702 | 0.14 | 4.56 | 0.07 | 0.41 | UP | Putative chloride channel-like protein CLC-g |
| Zm00001d027601 | 0.33 | 9.74 | 0.83 | 3.27 | UP | cytochrome P450 family 96 subfamily A polypeptide 1 |
| Zm00001d039536 | 0.00 | 0.42 | 0.28 | 2.54 | UP | Syntaxin-22 |
| Zm00001d020607 | 2.62 | 74.67 | 4.34 | 43.20 | UP | Dormancy-associated protein homolog 3 |
| Zm00001d006591 | 1.83 | 51.00 | 1.81 | 13.54 | UP | F-box domain containing protein |
| Zm00001d044604 | 0.73 | 20.27 | 0.45 | 1.05 | UP | Putative serine/threonine-protein kinase-like protein CCR3 |
| Zm00001d029706 | 7.68 | 196.59 | 17.28 | 46.89 | UP | glutathione transferase39 |
| Zm00001d025508 | 116.24 | 2965.25 | 71.65 | 649.15 | UP | Class IV heat shock protein |
| Zm00001d006813 | 0.27 | 6.75 | 0.97 | 4.34 | UP | Nuclear transcription factor Y subunit B-2 |
| Zm00001d047841 | 62.56 | 1532.35 | 307.30 | 1558.08 | UP | 17.4 kDa class I heat shock protein |
| Zm00001d034130 | 9.10 | 216.54 | 7.24 | 15.40 | UP | - |
| Zm00001d032669 | 2.11 | 48.46 | 1.12 | 2.63 | UP | glutaredoxin-related |
| Zm00001d053266 | 0.69 | 15.69 | 8.85 | 34.31 | UP | - |
| Zm00001d024598 | 0.11 | 2.54 | 0.31 | 0.83 | UP | Factor of DNA methylation 1 |
| Zm00001d033943 | 5.42 | 122.01 | 1.02 | 3.60 | UP | - |
| Zm00001d042541 | 13.75 | 309.36 | 2.20 | 4.64 | UP | lipoxygenase2 |
| Zm00001d003315 | 0.53 | 11.86 | 0.63 | 2.87 | UP | Protein Brevis radix-like 4 |
| Zm00001d052874 | 0.03 | 0.66 | 3.75 | 13.01 | UP | - |
| Zm00001d051692 | 0.21 | 4.33 | 0.72 | 2.15 | UP | Aspartyl protease AED1 |
| Zm00001d017813 | 15.26 | 311.38 | 20.37 | 292.66 | UP | 17.4 kDa class I heat shock protein 3 |
| Zm00001d012048 | 0.83 | 16.34 | 0.90 | 9.23 | UP | Putative leucine-rich repeat receptor-like protein kinase family protein |
| Zm00001d002979 | 0.02 | 0.49 | 0.00 | 0.16 | UP | Protein kinase superfamily protein |
| Zm00001d037165 | 0.25 | 4.86 | 0.31 | 1.72 | UP | Putative AP2/EREBP transcription factor superfamily protein |
| Zm00001d052744 | 5.00 | 91.71 | 9.42 | 22.47 | UP | Lipopolysaccharide-modifying protein |
| Zm00001d019163 | 95.65 | 1719.79 | 202.19 | 442.30 | UP | Stachyose synthase |
| Zm00001d028561 | 235.22 | 4221.68 | 466.98 | 3176.30 | UP | 17.4 kDa class I heat shock protein |
| Zm00001d006493 | 0.38 | 6.36 | 0.57 | 2.53 | UP | Protein DETOXIFICATION 40 |
| Zm00001d034756 | 17.33 | 286.94 | 42.28 | 143.39 | UP | - |
| Zm00001d017290 | 1.84 | 29.25 | 2.83 | 7.11 | UP | Late embryogenesis abundant (LEA) hydroxyproline-rich glycoprotein family |
| Zm00001d051161 | 6.35 | 100.25 | 14.19 | 39.33 | UP | phenylalanine ammonia lyase3 |
| Zm00001d033188 | 0.23 | 3.56 | 1.29 | 3.09 | UP | UDP-glucose 6-dehydrogenase |
| Zm00001d038061 | 3.74 | 58.60 | 1.13 | 4.54 | UP | Probable calcium-binding protein CML25 |
| Zm00001d011785 | 1.96 | 30.22 | 1.83 | 7.29 | UP | Transcription factor LUX |
| Zm00001d046147 | 0.38 | 5.81 | 0.39 | 7.24 | UP | E3 ubiquitin-protein ligase ATL41 |
| Zm00001d034031 | 5.81 | 85.63 | 4.83 | 10.06 | UP | - |
| Zm00001d044728 | 35.14 | 512.67 | 92.78 | 239.65 | UP | Class I heat shock protein 3 |
| Zm00001d049218 | 2.72 | 39.70 | 1.47 | 4.76 | UP | Two-pore potassium channel 1 |
| Zm00001d004437 | 0.85 | 12.32 | 0.86 | 2.17 | UP | THAUMATIN-LIKE PROTEIN 1 |
| Zm00001d045017 | 7.50 | 106.02 | 16.68 | 33.40 | UP | DUF1677 family protein |
| Zm00001d039140 | 0.39 | 5.53 | 1.03 | 3.68 | UP | Plant basic secretory protein (BSP) family protein |
| Zm00001d044426 | 7.39 | 104.18 | 10.35 | 29.32 | UP | DnaJ-like protein |
| Zm00001d027524 | 8.95 | 122.00 | 12.32 | 35.64 | UP | Basic endochitinase B |
| Zm00001d051792 | 1.31 | 17.75 | 4.14 | 11.59 | UP | ankyrin repeat family protein / regulator of chromosome condensation (RCC1) family protein |
| Zm00001d016655 | 34.88 | 466.89 | 93.80 | 220.27 | UP | - |
| Zm00001d014429 | 1.89 | 24.26 | 1.00 | 5.64 | UP | Retrotransposon-like protein |
| Zm00001d016558 | 0.28 | 3.58 | 0.52 | 1.49 | UP | glycosyl hydrolase family 10 protein / carbohydrate-binding domain-containing protein |
| Zm00001d015434 | 1.79 | 22.38 | 2.03 | 6.38 | UP | B-box zinc finger family protein |
| Zm00001d045311 | 1.52 | 18.82 | 6.38 | 13.82 | UP | Hydrolase-like protein family |
| Zm00001d002783 | 0.11 | 1.29 | 0.35 | 2.82 | UP | Subtilisin-like protease SBT2.4 |
| Zm00001d030856 | 0.83 | 10.09 | 0.04 | 0.44 | UP | Cytochrome P450 CYP81N4 |
| Zm00001d046471 | 85.45 | 1017.93 | 148.73 | 566.69 | UP | Multiprotein-bridging factor 1c |
| Zm00001d032873 | 34.96 | 415.41 | 156.48 | 314.69 | UP | Metal ion binding protein |
| Zm00001d020017 | 0.02 | 0.21 | 1.41 | 3.73 | UP | Subtilisin-like protease SBT1.9 |
| Zm00001d032825 | 185.38 | 2151.20 | 79.45 | 476.87 | UP | - |
| Zm00001d053433 | 0.33 | 3.92 | 5.93 | 13.32 | UP | Putative RING zinc finger domain superfamily protein |
| Zm00001d051938 | 4.32 | 49.24 | 0.76 | 2.80 | UP | Cinnamoyl-CoA reductase 1 |
| Zm00001d009025 | 0.14 | 1.53 | 0.13 | 0.85 | UP | Probable metal-nicotianamine transporter YSL7 |
| Zm00001d017984 | 17.52 | 193.12 | 28.21 | 66.04 | UP | Probable galacturonosyltransferase-like 9 |
| Zm00001d004401 | 200.06 | 2189.05 | 96.13 | 269.43 | UP | Germin-like protein subfamily 1 member 8 |
| Zm00001d042065 | 1.86 | 20.26 | 1.71 | 9.68 | UP | P-loop containing nucleoside triphosphate hydrolases superfamily protein |
| Zm00001d052807 | 0.20 | 1.98 | 0.36 | 3.40 | UP | Putative bZIP transcription factor superfamily protein |
| Zm00001d028408 | 202.40 | 2047.80 | 449.59 | 1358.92 | UP | heat shock protein26 |
| Zm00001d042540 | 11.55 | 116.27 | 1.31 | 10.98 | UP | Linoleate 9S-lipoxygenase 1 |
| Zm00001d034423 | 22.76 | 226.48 | 18.21 | 43.61 | UP | Tetratricopeptide repeat (TPR)-like superfamily protein |
| Zm00001d034897 | 9.94 | 96.25 | 6.73 | 14.68 | UP | Plastid-specific 30S ribosomal protein 1 |
| Zm00001d036950 | 15.30 | 147.68 | 7.28 | 30.38 | UP | - |
| Zm00001d046574 | 15.13 | 144.97 | 13.59 | 31.08 | UP | - |
| Zm00001d028588 | 16.31 | 153.66 | 28.65 | 119.52 | UP | Fes1B |
| Zm00001d010814 | 98.06 | 895.08 | 138.98 | 406.93 | UP | AtS40-3 |
| Zm00001d039942 | 104.91 | 941.49 | 201.27 | 1683.90 | UP | 17.4 kDa class I heat shock protein |
| Zm00001d053091 | 4.96 | 44.41 | 8.31 | 21.32 | UP | Adagio protein 3 |
| Zm00001d044898 | 3.18 | 28.15 | 2.62 | 7.35 | UP | Copper ion binding protein |
| Zm00001d053995 | 2.55 | 22.37 | 1.02 | 3.85 | UP | - |
| Zm00001d007445 | 2.97 | 25.99 | 3.69 | 8.72 | UP | Adagio protein 3 |
| Zm00001d014562 | 1.32 | 11.31 | 3.68 | 8.07 | UP | IAA-amino acid hydrolase ILR1-like 6 |
| Zm00001d000523 | 0.64 | 5.53 | 1.33 | 2.72 | UP | - |
| Zm00001d038164 | 30.16 | 254.14 | 67.00 | 192.69 | UP | methyl binding domain |
| Zm00001d010840 | 26.56 | 223.67 | 0.04 | 3.25 | UP | triacylglycerol lipase-like 1 |
| Zm00001d038915 | 0.04 | 0.33 | 0.03 | 0.78 | UP | Putative WAK receptor-like protein kinase family protein |
| Zm00001d002823 | 57.05 | 473.81 | 105.55 | 326.52 | UP | Hsp70-Hsp90 organizing protein 3 |
| Zm00001d039566 | 817.74 | 6712.37 | 2838.58 | 7275.93 | UP | 17.5 kDa class II heat shock protein |
| Zm00001d018746 | 1.73 | 14.10 | 2.90 | 8.17 | UP | - |
| Zm00001d003276 | 1.38 | 10.85 | 1.52 | 4.27 | UP | S-adenosyl-L-methionine-dependent methyltransferases superfamily protein |
| Zm00001d009626 | 16.69 | 131.21 | 20.09 | 44.01 | UP | protein phosphatase homolog9 |
| Zm00001d022597 | 2.82 | 21.81 | 25.03 | 51.42 | UP | NEP1-interacting protein-like 1 |
| Zm00001d047424 | 8.09 | 61.98 | 15.48 | 40.12 | UP | Flavonoid 3'-monooxygenase |
| Zm00001d043586 | 0.20 | 1.64 | 4.00 | 15.38 | UP | - |
| Zm00001d031325 | 41.06 | 306.46 | 39.42 | 491.90 | UP | 25.3 kDa heat shock protein chloroplastic |
| Zm00001d027958 | 2.18 | 16.15 | 0.29 | 2.05 | UP | - |
| Zm00001d037550 | 1.38 | 10.19 | 0.11 | 1.49 | UP | peroxidase 5 |
| Zm00001d045459 | 5.69 | 41.76 | 13.81 | 62.56 | UP | - |
| Zm00001d052757 | 0.53 | 3.79 | 0.47 | 2.32 | UP | Casparian strip membrane protein 4 |
| Zm00001d010529 | 76.69 | 543.32 | 160.27 | 342.50 | UP | Probable mediator of RNA polymerase II transcription subunit 37c |
| Zm00001d045544 | 32.05 | 227.01 | 76.13 | 161.88 | UP | Chaperonin 60 subunit beta 2 chloroplastic |
| Zm00001d014329 | 0.25 | 1.77 | 0.03 | 0.83 | UP | - |
| Zm00001d043362 | 0.47 | 3.27 | 0.73 | 3.94 | UP | - |
| Zm00001d049647 | 14.10 | 98.73 | 10.58 | 33.92 | UP | Ribosome-like protein |
| Zm00001d024903 | 393.38 | 2695.11 | 647.71 | 2302.47 | UP | heat shock protein 90 kDa |
| Zm00001d028820 | 37.33 | 252.80 | 46.08 | 123.62 | UP | Chaperone DnaJ-domain superfamily protein |
| Zm00001d052194 | 388.61 | 2625.02 | 760.63 | 2100.22 | UP | 23.6 kDa heat shock protein mitochondrial |
| Zm00001d036768 | 11.36 | 76.46 | 33.83 | 70.53 | UP | Transcription factor MYB44 |
| Zm00001d044841 | 5.79 | 38.76 | 8.90 | 20.67 | UP | Glutaredoxin family protein |
| Zm00001d026975 | 0.29 | 1.92 | 2.21 | 8.20 | UP | - |
| Zm00001d005798 | 21.09 | 137.31 | 99.07 | 221.13 | UP | Ethylene-responsive transcription factor RAP2-2 |
| Zm00001d020583 | 35.78 | 230.38 | 53.77 | 175.03 | UP | Galactosyltransferase family protein |
| Zm00001d008181 | 16.03 | 102.63 | 4.27 | 11.52 | UP | - |
| Zm00001d009557 | 6.62 | 41.48 | 8.12 | 49.91 | UP | DUF679 domain membrane protein 7 |
| Zm00001d013489 | 23.40 | 141.78 | 33.68 | 167.51 | UP | splicing factor Prp18 family protein |
| Zm00001d038637 | 2.80 | 16.60 | 2.01 | 6.05 | UP | - |
| ZeamMp161 | 3.00 | 17.57 | 1.93 | 4.41 | UP | cox3 |
| Zm00001d031740 | 36.96 | 216.10 | 68.86 | 175.51 | UP | Activator of 90 kDa heat shock protein ATPase |
| Zm00001d004731 | 8.75 | 50.86 | 7.85 | 18.22 | UP | Aldehyde dehydrogenase |
| Zm00001d038281 | 1.27 | 7.39 | 4.61 | 9.50 | UP | Dof zinc finger protein DOF1.2 |
| Zm00001d028266 | 0.82 | 4.68 | 0.97 | 3.25 | UP | Major facilitator superfamily protein |
| Zm00001d002541 | 0.27 | 1.53 | 0.30 | 0.83 | UP | Transducin/WD40 repeat-like superfamily protein |
| Zm00001d018297 | 14.33 | 81.70 | 28.23 | 64.65 | UP | Chaperone DnaJ-domain superfamily protein |
| Zm00001d003924 | 8.70 | 49.47 | 5.15 | 13.70 | UP | Glycerophosphodiester phosphodiesterase GDPD2 |
| Zm00001d050119 | 13.18 | 74.84 | 17.94 | 70.91 | UP | Activator of heat shock protein ATPase |
| Zm00001d033172 | 3.89 | 22.11 | 4.30 | 11.53 | UP | Regulator of chromosome condensation (RCC1) family protein |
| Zm00001d006787 | 1.08 | 6.07 | 1.49 | 4.95 | UP | Agenet domain-containing protein |
| Zm00001d046142 | 0.25 | 1.42 | 0.00 | 0.58 | UP | E3 ubiquitin-protein ligase ATL41 |
| Zm00001d015093 | 1.15 | 6.38 | 2.00 | 5.52 | UP | RNA-binding KH domain-containing protein PEPPER |
| Zm00001d045669 | 15.52 | 85.37 | 23.25 | 48.75 | UP | Acylphosphatase |
| Zm00001d012561 | 19.44 | 106.90 | 96.55 | 262.93 | UP | Cystatin2%3B Cysteine proteinase inhibitor%3B Putative cystatin |
| Zm00001d007764 | 4.17 | 22.76 | 4.09 | 8.31 | UP | Hydroxyproline O-galactosyltransferase GALT4 |
| Zm00001d029320 | 0.75 | 4.14 | 4.81 | 9.91 | UP | Probable polyamine transporter |
| Zm00001d013111 | 49.40 | 267.08 | 155.57 | 468.40 | UP | DnaJ protein |
| Zm00001d012169 | 4.29 | 23.10 | 7.71 | 19.88 | UP | RHOMBOID-like protein 13 |
| Zm00001d045476 | 11.92 | 63.60 | 25.29 | 60.21 | UP | - |
| Zm00001d028557 | 267.20 | 1422.85 | 1370.09 | 3332.28 | UP | 17.4 kDa class I heat shock protein |
| Zm00001d047687 | 11.74 | 62.45 | 23.08 | 53.65 | UP | Chaperone DnaJ-domain superfamily protein |
| Zm00001d006821 | 29.77 | 155.35 | 25.85 | 54.72 | UP | Mitochondrial import inner membrane translocase subunit TIM17-2 |
| Zm00001d013289 | 14.03 | 72.94 | 18.41 | 66.58 | UP | Transmembrane BAX inhibitor motif-containing protein 4 |
| Zm00001d037757 | 24.34 | 126.75 | 41.48 | 99.46 | UP | Grx_C15-glutaredoxin subgroup III |
| Zm00001d033990 | 57.58 | 298.76 | 149.89 | 709.09 | UP | Bax inhibitor-1 family protein |
| Zm00001d040731 | 6.50 | 33.67 | 22.88 | 56.03 | UP | Alpha-L-fucosidase 2 |
| Zm00001d051413 | 0.54 | 2.65 | 4.18 | 57.59 | UP | CGI-like protein |
| Zm00001d051415 | 17.45 | 88.61 | 19.21 | 63.68 | UP | Mechanosensitive ion channel protein 6 |
| Zm00001d018177 | 16.74 | 84.97 | 67.49 | 220.39 | UP | - |
| Zm00001d015308 | 5.57 | 28.12 | 7.47 | 16.30 | UP | - |
| Zm00001d002488 | 1.53 | 7.76 | 1.20 | 8.57 | UP | DUF740 family protein |
| Zm00001d045036 | 58.57 | 295.46 | 69.10 | 283.12 | UP | Chaperone DnaJ-domain superfamily protein |
| Zm00001d014060 | 0.16 | 0.78 | 1.26 | 3.17 | UP | Protein NRT1/ PTR FAMILY 5.2 |
| Zm00001d021639 | 1.30 | 6.53 | 0.25 | 0.78 | UP | Taxane 13-alpha-hydroxylase |
| Zm00001d032481 | 34.46 | 173.27 | 12.12 | 47.34 | UP | - |
| Zm00001d008841 | 828.31 | 4152.88 | 949.12 | 3124.64 | UP | 17.8 kDa class II heat shock protein |
| Zm00001d012710 | 15.84 | 79.00 | 20.19 | 41.97 | UP | SGS domain-containing protein |
| ZeamMp094 | 1.08 | 5.34 | 1.43 | 4.73 | UP | orf140-b |
| Zm00001d018298 | 127.20 | 631.19 | 217.51 | 525.27 | UP | 17.4 kDa class III heat shock protein |
| Zm00001d002447 | 1.13 | 5.60 | 2.17 | 5.39 | UP | Wall-associated receptor kinase 2 |
| Zm00001d019510 | 3.14 | 15.51 | 3.77 | 8.43 | UP | Folate/biopterin transporter family protein |
| Zm00001d015430 | 0.55 | 2.73 | 0.75 | 2.43 | UP | Putative clathrin assembly protein |
| Zm00001d012041 | 7.09 | 34.50 | 7.67 | 20.54 | UP | - |
| Zm00001d027598 | 1.01 | 4.93 | 1.91 | 6.45 | UP | CCT motif family protein |
| Zm00001d012069 | 2.06 | 9.87 | 4.42 | 10.16 | UP | DUF506 family protein |
| Zm00001d039822 | 1.11 | 5.32 | 1.91 | 4.27 | UP | LRR receptor-like serine/threonine-protein kinase EFR |
| Zm00001d005109 | 13.00 | 61.56 | 15.96 | 38.87 | UP | - |
| Zm00001d022130 | 80.46 | 379.73 | 60.03 | 146.88 | UP | DUF1677 family protein |
| Zm00001d020903 | 9.07 | 42.40 | 6.47 | 13.68 | UP | Fasciclin-like arabinogalactan protein 7 |
| Zm00001d033714 | 7.30 | 34.17 | 2.28 | 7.54 | UP | Regulator of Vps4 activity in the MVB pathway protein |
| Zm00001d041955 | 2.37 | 11.03 | 2.21 | 6.10 | UP | plant-specific domain TIGR01615 family protein expressed |
| Zm00001d031569 | 126.79 | 584.85 | 208.70 | 440.14 | UP | Peptidyl-prolyl isomerase |
| Zm00001d012485 | 3.26 | 15.00 | 1.31 | 4.66 | UP | Transmembrane amino acid transporter family protein |
| Zm00001d038959 | 2.18 | 10.02 | 4.19 | 8.52 | UP | O-acyltransferase WSD1 |
| Zm00001d039280 | 8.60 | 39.14 | 11.46 | 23.99 | UP | Probable ubiquitin-conjugating enzyme E2 25 |
| Zm00001d013555 | 2.53 | 11.42 | 3.54 | 7.97 | UP | Growth-regulating factor 2 |
| Zm00001d048667 | 3.53 | 15.88 | 4.30 | 11.62 | UP | Protein kinase domain superfamily protein |
| Zm00001d053229 | 4.62 | 20.60 | 4.28 | 11.38 | UP | - |
| Zm00001d047857 | 26.53 | 118.47 | 41.59 | 113.80 | UP | glycine-rich protein |
| Zm00001d014489 | 1.91 | 8.48 | 2.47 | 5.61 | UP | Putative beta-glucosidase 41 |
| Zm00001d000113 | 6.67 | 29.21 | 6.07 | 15.29 | UP | - |
| Zm00001d009556 | 41.55 | 182.15 | 48.01 | 138.88 | UP | HSP40/DnaJ peptide-binding protein |
| Zm00001d028269 | 2.93 | 12.80 | 1.65 | 4.07 | UP | ATP-dependent DNA helicase [Source:UniProtKB/TrEMBL;Acc:A0A1D6JU37] |
| Zm00001d014486 | 117.80 | 514.45 | 275.63 | 650.52 | UP | Phosphosulfolactate synthase-related protein |
| Zm00001d011589 | 3.97 | 17.30 | 3.66 | 16.98 | UP | NAC domain-containing protein 72 |
| Zm00001d051139 | 22.55 | 98.40 | 17.91 | 41.16 | UP | RNA-binding (RRM/RBD/RNP motifs) family protein |
| Zm00001d042626 | 6.58 | 28.62 | 8.08 | 23.18 | UP | nitroreductase family protein |
| Zm00001d051790 | 8.40 | 36.43 | 5.12 | 10.90 | UP | APO protein 2 chloroplastic |
| Zm00001d045435 | 46.11 | 199.65 | 67.35 | 166.67 | UP | glycine-rich protein |
| Zm00001d016512 | 8.05 | 34.84 | 13.31 | 26.91 | UP | Peptidyl-prolyl cis-trans isomerase FKBP65 |
| Zm00001d012335 | 2.30 | 9.90 | 0.29 | 0.85 | UP | cis-zeatin O-glucosyltransferase2 |
| Zm00001d020280 | 2.83 | 12.20 | 0.12 | 0.42 | UP | Major facilitator superfamily protein |
| Zm00001d052837 | 7.96 | 34.31 | 14.67 | 41.93 | UP | BTB/POZ domain-containing protein |
| Zm00001d032405 | 0.89 | 3.80 | 0.46 | 1.69 | UP | Peroxidase 1 |
| Zm00001d013202 | 15.13 | 64.11 | 16.03 | 33.01 | UP | DNA binding protein |
| Zm00001d013795 | 12.70 | 53.80 | 18.20 | 46.09 | UP | Peroxisomal membrane 22 kDa (Mpv17/PMP22) family protein |
| Zm00001d001951 | 69.88 | 293.93 | 96.26 | 192.12 | UP | PHD finger-like domain-containing protein 5A |
| Zm00001d041725 | 2.88 | 12.05 | 0.30 | 1.54 | UP | Pollen-specific protein SF3 |
| Zm00001d008214 | 12.30 | 51.67 | 12.94 | 69.16 | UP | Rhomboid-like protein 14 mitochondrial |
| Zm00001d048073 | 66.77 | 278.31 | 240.07 | 860.40 | UP | Heat shock 70 kDa protein 8 |
| Zm00001d004472 | 26.42 | 109.97 | 45.22 | 128.56 | UP | Polyketide cyclase/dehydrase and lipid transport superfamily protein |
| Zm00001d031443 | 17.78 | 73.00 | 44.67 | 140.89 | UP | DUF2361 family protein |
| Zm00001d033084 | 12.41 | 51.15 | 20.63 | 48.18 | UP | - |
| Zm00001d047955 | 16.91 | 68.46 | 10.96 | 22.62 | UP | Stress inducible protein coi6.1 [Source:UniProtKB/TrEMBL;Acc:C4J684] |
| Zm00001d031338 | 13.49 | 53.69 | 25.33 | 66.23 | UP | Wall-associated receptor kinase 1 |
| Zm00001d042274 | 8.01 | 31.69 | 1.08 | 4.59 | UP | Sec14p-like phosphatidylinositol transfer family protein |
| Zm00001d018461 | 45.67 | 179.19 | 46.69 | 135.23 | UP | Thioredoxin |
| Zm00001d042039 | 0.65 | 2.55 | 0.24 | 0.63 | UP | empty pericarp5 |
| Zm00001d014972 | 10.43 | 40.45 | 12.27 | 25.01 | UP | CGI-like protein |
| Zm00001d031436 | 5.68 | 21.77 | 13.46 | 28.05 | UP | OTU domain-containing protein |
| Zm00001d009359 | 17.90 | 68.52 | 22.24 | 58.08 | UP | Ubiquitin fusion degradation UFD1 family protein |
| Zm00001d018217 | 4.51 | 17.21 | 4.09 | 9.09 | UP | (Rice Genome Annotation Project) expressed protein |
| Zm00001d033872 | 35.34 | 133.77 | 85.73 | 173.02 | UP | Peptidase%2C M50 family |
| Zm00001d011164 | 2.05 | 7.84 | 1.76 | 5.25 | UP | - |
| Zm00001d026094 | 13.09 | 49.35 | 16.55 | 94.43 | UP | Heat stress transcription factor B-2b |
| Zm00001d012233 | 0.83 | 3.16 | 1.22 | 3.04 | UP | PHD finger protein |
| Zm00001d018916 | 2.59 | 9.67 | 2.43 | 5.98 | UP | PIF / Ping-Pong family of plant transposases |
| Zm00001d038301 | 1.03 | 3.88 | 1.53 | 3.31 | UP | - |
| Zm00001d003277 | 3.63 | 13.40 | 8.60 | 21.00 | UP | Stachyose synthase |
| Zm00001d015374 | 1.46 | 5.40 | 3.08 | 6.15 | UP | plant/F14G9-20 protein |
| Zm00001d045488 | 4.43 | 16.29 | 4.63 | 10.19 | UP | Nudix hydrolase 14 chloroplastic |
| Zm00001d038548 | 0.09 | 0.33 | 0.14 | 0.63 | UP | - |
| Zm00001d013610 | 0.81 | 2.97 | 2.23 | 5.56 | UP | Osmotin-like protein OSM34 |
| Zm00001d036535 | 1.20 | 4.35 | 0.04 | 0.63 | UP | oxygen evolving complex 33kDa subunit |
| Zm00001d022618 | 13.50 | 48.86 | 9.84 | 24.33 | UP | SKP1-like protein 1A |
| Zm00001d009410 | 3.59 | 12.88 | 5.62 | 11.40 | UP | EMB2752 |
| Zm00001d020976 | 37.22 | 133.06 | 43.98 | 177.20 | UP | - |
| Zm00001d053394 | 52.35 | 186.85 | 72.27 | 200.34 | UP | - |
| Zm00001d010902 | 8.72 | 30.94 | 10.30 | 22.56 | UP | Phosphoglycerate mutase-like protein AT74H |
| Zm00001d038815 | 11.05 | 39.14 | 13.41 | 37.76 | UP | CASC3/Barentsz eIF4AIII binding |
| Zm00001d002584 | 16.38 | 57.95 | 35.76 | 100.71 | UP | - |
| Zm00001d049377 | 4.74 | 16.73 | 4.37 | 9.11 | UP | General transcription factor 2-related zinc finger protein |
| Zm00001d004810 | 1.17 | 4.13 | 1.90 | 6.77 | UP | FHA domain-containing protein PS1 |
| Zm00001d039000 | 104.01 | 365.32 | 123.83 | 365.60 | UP | HSP40/DnaJ peptide-binding protein |
| Zm00001d015697 | 7.22 | 24.81 | 10.76 | 31.83 | UP | - |
| Zm00001d039001 | 21.26 | 73.47 | 48.45 | 111.00 | UP | Expressed protein; protein |
| Zm00001d047478 | 0.61 | 2.09 | 36.26 | 143.95 | UP | - |
| Zm00001d044328 | 1.56 | 5.32 | 0.77 | 1.54 | UP | rbcl rna s1-binding domain protein1 |
| Zm00001d014091 | 5.24 | 17.68 | 5.61 | 14.13 | UP | F-box domain containing protein |
| Zm00001d025490 | 1.69 | 5.65 | 3.44 | 11.90 | UP | Zea mays ARGOS1 |
| Zm00001d027597 | 6.95 | 23.08 | 10.88 | 32.41 | UP | - |
| Zm00001d034017 | 31.15 | 103.19 | 37.43 | 99.32 | UP | Exhydrolase II |
| Zm00001d002006 | 27.02 | 88.93 | 29.21 | 68.14 | UP | H(+)-ATPase 5 |
| Zm00001d047045 | 23.25 | 76.46 | 25.50 | 56.40 | UP | Plant intracellular Ras-group-related LRR protein 4 |
| Zm00001d011640 | 1.02 | 3.37 | 3.30 | 11.38 | UP | - |
| Zm00001d037687 | 21.35 | 70.01 | 25.18 | 76.92 | UP | derlin3 |
| Zm00001d026366 | 0.65 | 2.14 | 0.53 | 1.57 | UP | Putative SNF2-domain/RING finger domain/helicase domain protein |
| Zm00001d002387 | 27.51 | 89.57 | 6.39 | 13.51 | UP | - |
| Zm00001d017099 | 23.34 | 75.73 | 36.29 | 72.80 | UP | RNI-like superfamily protein |
| Zm00001d004771 | 0.44 | 1.41 | 0.57 | 1.18 | UP | - |
| Zm00001d042443 | 1.81 | 5.85 | 2.85 | 7.58 | UP | BTB/POZ and TAZ domain-containing protein 2 |
| Zm00001d032013 | 1.37 | 4.38 | 3.72 | 15.54 | UP | - |
| Zm00001d028531 | 16.69 | 52.73 | 17.78 | 47.41 | UP | glycine-rich protein |
| Zm00001d003002 | 4.04 | 12.75 | 5.42 | 12.94 | UP | IAA-amino acid hydrolase ILR1-like 3 |
| Zm00001d034237 | 7.40 | 23.30 | 7.73 | 21.89 | UP | Probable LRR receptor-like serine/threonine-protein kinase |
| Zm00001d005470 | 0.40 | 1.21 | 8.54 | 30.60 | UP | - |
| Zm00001d026463 | 2.70 | 8.48 | 3.57 | 7.40 | UP | Reactive oxygen species modulator 1 |
| Zm00001d035974 | 20.37 | 63.71 | 28.29 | 60.30 | UP | Chaperone protein dnaJ |
| Zm00001d031019 | 10.70 | 33.45 | 20.31 | 45.85 | UP | Homocysteine S-methyltransferase 2 |
| Zm00001d010823 | 4.49 | 14.02 | 7.01 | 20.22 | UP | CASC3/Barentsz eIF4AIII binding |
| Zm00001d004772 | 2.06 | 6.39 | 2.69 | 6.25 | UP | - |
| Zm00001d009103 | 88.21 | 272.19 | 64.82 | 138.45 | UP | Ethylene-responsive factor-like protein |
| Zm00001d039658 | 27.01 | 81.99 | 24.70 | 68.95 | UP | Transcription factor HY5-like |
| Zm00001d037384 | 14.84 | 44.68 | 18.88 | 64.93 | UP | Anthocyanidin 3-O-glucosyltransferase |
| Zm00001d003003 | 6.84 | 20.57 | 9.46 | 19.67 | UP | OSJNBa0019D11.18 protein; protein |
| Zm00001d047302 | 24.10 | 72.39 | 31.68 | 67.21 | UP | Putative chaperone clbp family protein |
| Zm00001d047765 | 34.65 | 103.79 | 7.34 | 31.13 | UP | Glutathione S-transferase L2 chloroplastic |
| Zm00001d042922 | 181.49 | 541.40 | 228.20 | 528.35 | UP | Probable mediator of RNA polymerase II transcription subunit 37c |
| Zm00001d023768 | 6.36 | 18.95 | 2.96 | 13.50 | UP | Delta(12)-fatty-acid desaturase |
| Zm00001d028287 | 1.91 | 5.66 | 0.30 | 3.10 | UP | Calcium uniporter protein 4 mitochondrial |
| ZeamMr004 | 27.32 | 80.53 | 28.06 | 62.69 | UP | rrn1818S ribosomal RNA |
| Zm00001d053626 | 3.62 | 10.56 | 4.24 | 9.78 | UP | SPX domain-containing protein 1 |
| Zm00001d051233 | 3.92 | 11.41 | 3.78 | 9.30 | UP | - |
| Zm00001d018238 | 0.61 | 1.78 | 1.54 | 4.44 | UP | Thioredoxin-like 3-1 chloroplastic |
| Zm00001d052738 | 63.33 | 182.84 | 90.84 | 237.89 | UP | Heat stress transcription factor B-2b |
| Zm00001d032657 | 3.11 | 8.85 | 3.68 | 29.90 | UP | Retrotransposon-like protein |
| Zm00001d005110 | 24.93 | 69.68 | 34.07 | 68.51 | UP | Serine/threonine-protein phosphatase |
| Zm00001d030755 | 0.67 | 1.89 | 0.67 | 2.96 | UP | - |
| Zm00001d045563 | 0.73 | 2.02 | 1.52 | 6.52 | UP | dwarf plant3 |
| Zm00001d034667 | 31.10 | 83.75 | 37.41 | 79.09 | UP | Fes1A |
| Zm00001d009950 | 35.61 | 95.61 | 41.08 | 104.55 | UP | Heat shock 70 kDa protein 14 |
| Zm00001d015227 | 0.36 | 0.97 | 0.28 | 0.94 | UP | chaperone binding;ATPase activators |
| Zm00001d002153 | 8.47 | 22.62 | 9.64 | 19.82 | UP | Heterogeneous nuclear ribonucleoprotein U-like protein 1 |
| Zm00001d033591 | 100.52 | 263.76 | 144.40 | 336.20 | UP | Heat shock 70 kDa protein 6 chloroplastic |
| Zm00001d036970 | 1.83 | 4.79 | 1.36 | 2.95 | UP | SMAD/FHA domain-containing protein |
| Zm00001d010652 | 7.24 | 18.82 | 1.12 | 3.80 | UP | EID1-like F-box protein 3 |
| Zm00001d027449 | 10.53 | 27.19 | 18.48 | 37.30 | UP | - |
| Zm00001d041325 | 1.65 | 4.23 | 2.51 | 5.12 | UP | Tetratricopeptide repeat (TPR)-like superfamily protein |
| Zm00001d028297 | 0.52 | 1.32 | 0.66 | 2.02 | UP | Transcription factor bHLH62 |
| Zm00001d016979 | 13.91 | 34.81 | 19.54 | 40.06 | UP | prohibitin3 |
| Zm00001d050518 | 3.66 | 9.05 | 4.44 | 10.73 | UP | MAC/Perforin domain containing protein |
| Zm00001d039343 | 2.49 | 6.15 | 3.20 | 7.34 | UP | - |
| Zm00001d048836 | 4.00 | 9.87 | 6.62 | 13.67 | UP | - |
| Zm00001d028906 | 14.72 | 36.11 | 19.86 | 44.26 | UP | - |
| Zm00001d038218 | 9.35 | 22.92 | 15.92 | 33.42 | UP | Cytochrome c-2 |
| Zm00001d013010 | 2.17 | 5.29 | 5.03 | 11.22 | UP | Peptidyl-prolyl cis-trans isomerase |
| Zm00001d025057 | 5.66 | 13.76 | 3.98 | 11.93 | UP | NAD(P)-linked oxidoreductase superfamily protein |
| Zm00001d032687 | 20.06 | 48.31 | 18.53 | 40.20 | UP | Protein Transporter Pam16 |
| Zm00001d021943 | 9.17 | 22.08 | 9.39 | 24.78 | UP | - |
| Zm00001d033396 | 1.25 | 2.98 | 0.53 | 3.07 | UP | Growth-regulating factor 2 |
| Zm00001d006593 | 1.79 | 4.26 | 1.48 | 3.04 | UP | Myosin-2 |
| Zm00001d034670 | 3.54 | 8.36 | 5.03 | 10.54 | UP | hAT transposon superfamily |
| Zm00001d036707 | 8.76 | 20.60 | 10.33 | 28.77 | UP | Putative ubiquitin carboxyl-terminal hydrolase superfamily protein |
| Zm00001d022889 | 3.82 | 8.96 | 5.90 | 12.06 | UP | - |
| Zm00001d034665 | 27.67 | 63.46 | 34.01 | 89.52 | UP | NAD-dependent epimerase/dehydratase |
| Zm00001d052651 | 14.98 | 34.20 | 13.92 | 29.67 | UP | Probable xyloglucan endotransglucosylase/hydrolase protein 5 |
| Zm00001d017539 | 24.64 | 56.05 | 27.88 | 119.26 | UP | Mechanosensitive ion channel protein 6 |
| Zm00001d048842 | 1.60 | 3.61 | 1.36 | 3.03 | UP | OSJNBa0089K21.9 protein; Os04g0471100 protein |
| Zm00001d012609 | 1.07 | 2.43 | 0.94 | 2.20 | UP | Serine/threonine-protein kinase |
| Zm00001d005329 | 10.96 | 24.77 | 12.97 | 26.68 | UP | Haloacid dehalogenase-like hydrolase (HAD) superfamily protein |
| Zm00001d039444 | 7.60 | 17.12 | 10.06 | 26.81 | UP | UNE1 |
| Zm00001d017481 | 1.01 | 2.28 | 15.10 | 48.87 | UP | - |
| Zm00001d015698 | 7.65 | 17.10 | 6.29 | 15.03 | UP | F-box/kelch-repeat protein OR23 |
| Zm00001d024645 | 10.73 | 23.98 | 15.34 | 34.52 | UP | alpha/beta-Hydrolases superfamily protein |
| Zm00001d006026 | 8.44 | 18.76 | 12.95 | 29.73 | UP | Lysine-specific demethylase JMJ30 |
| Zm00001d051645 | 1.74 | 3.85 | 16.94 | 84.88 | UP | Tetraspanin-6 |
| Zm00001d029067 | 3.92 | 8.63 | 6.44 | 13.34 | UP | Plant-specific domain TIGR01568 family protein |
| Zm00001d019216 | 31.04 | 67.78 | 56.96 | 185.69 | UP | Ethylene-responsive transcription factor ERF118 |
| Zm00001d021444 | 0.66 | 1.43 | 2.67 | 13.05 | UP | Monooxygenase/ oxidoreductase |
| Zm00001d052200 | 6.74 | 14.38 | 10.06 | 22.67 | UP | F-box protein FBW2 |
| Zm00001d010796 | 7.07 | 14.95 | 11.63 | 23.82 | UP | hexokinase3 |
| Zm00001d045217 | 5.42 | 11.44 | 4.92 | 10.22 | UP | Subtilisin-like protease SBT6.1 |
| Zm00001d028362 | 22.85 | 48.33 | 41.03 | 85.07 | UP | - |
| Zm00001d053102 | 23.87 | 50.01 | 28.13 | 70.93 | UP | Tubulin alpha-6 chain |
| Zm00001d014717 | 2.20 | 4.58 | 2.83 | 6.07 | UP | Glycosyltransferase family 64 protein C4 |
| Zm00001d028641 | 12.29 | 24.64 | 10.65 | 22.26 | UP | - |
| Zm00001d005370 | 1.86 | 0.92 | 1.75 | 0.80 | DOWN | GPI mannosyltransferase 1 |
| Zm00001d002744 | 9.41 | 4.62 | 12.71 | 5.64 | DOWN | Dehydration-responsive element-binding protein 1C |
| Zm00001d001858 | 3.01 | 1.48 | 3.16 | 0.99 | DOWN | BTB/POZ domain-containing protein |
| Zm00001d014813 | 1.60 | 0.78 | 0.93 | 0.34 | DOWN | Serine/threonine-protein kinase ATR |
| Zm00001d017480 | 13.89 | 6.69 | 25.55 | 9.15 | DOWN | Ethylene-responsive transcription factor ERF035 |
| Zm00001d002240 | 3.30 | 1.58 | 1.03 | 0.23 | DOWN | Chloroplastic lipocalin |
| Zm00001d027527 | 16.52 | 7.91 | 22.52 | 10.52 | DOWN | 40S ribosomal protein Sa-1 |
| Zm00001d018981 | 365.24 | 174.19 | 277.04 | 70.17 | DOWN | histone1a |
| Zm00001d032148 | 2.21 | 1.05 | 2.21 | 0.82 | DOWN | hydroxycinnamoyltransferase9 |
| Zm00001d014443 | 3.78 | 1.80 | 5.42 | 1.85 | DOWN | Serine carboxypeptidase-like 19 |
| Zm00001d021599 | 1.98 | 0.94 | 0.81 | 0.24 | DOWN | SKU5 similar 4 |
| Zm00001d034722 | 8.96 | 4.23 | 23.06 | 10.04 | DOWN | - |
| Zm00001d025886 | 12.57 | 5.94 | 34.88 | 17.31 | DOWN | Cytokinin-O-glucosyltransferase 3 |
| Zm00001d043350 | 22.54 | 10.61 | 41.97 | 14.86 | DOWN | Probable indole-3-acetic acid-amido synthetase GH3.1 |
| Zm00001d024753 | 57.87 | 27.09 | 33.30 | 15.24 | DOWN | peroxidase35 |
| Zm00001d051554 | 3.35 | 1.57 | 5.73 | 1.58 | DOWN | abscisic acid 8'-hydroxylase2 |
| Zm00001d050310 | 32.09 | 14.87 | 12.11 | 5.64 | DOWN | Homeodomain leucine zipper protein |
| Zm00001d035722 | 51.02 | 23.53 | 28.90 | 13.69 | DOWN | Actin-7 |
| Zm00001d021452 | 11.86 | 5.47 | 13.26 | 5.28 | DOWN | BZIP transcription factor |
| Zm00001d048138 | 2.23 | 1.02 | 1.30 | 0.43 | DOWN | BTB/POZ domain-containing protein NPY2 |
| Zm00001d040693 | 1.72 | 0.79 | 11.72 | 5.51 | DOWN | Tetratricopeptide repeat (TPR)-like superfamily protein |
| Zm00001d008850 | 16.60 | 7.61 | 15.48 | 6.96 | DOWN | Late embryogenesis abundant (LEA) hydroxyproline-rich glycoprotein family |
| Zm00001d017857 | 68.34 | 31.19 | 92.65 | 43.88 | DOWN | adenine nucleotide translocator1 |
| Zm00001d053801 | 11.24 | 5.02 | 9.35 | 4.09 | DOWN | Phosphatase IMPL1 chloroplastic |
| Zm00001d034776 | 9.68 | 4.33 | 7.84 | 3.62 | DOWN | Elongation factor 2 |
| Zm00001d000021 | 197.34 | 88.21 | 174.35 | 86.57 | DOWN | glucose 6-phosphate/phosphate translocator1 |
| Zm00001d049441 | 19.57 | 8.74 | 26.67 | 12.66 | DOWN | Glucose-6-phosphate/phosphate translocator 2 |
| Zm00001d034479 | 107.41 | 47.55 | 75.79 | 16.34 | DOWN | histone one (H1) |
| Zm00001d012884 | 3.03 | 1.34 | 2.14 | 0.94 | DOWN | FAD/NAD(P)-binding oxidoreductase family protein |
| Zm00001d026415 | 3.07 | 1.35 | 1.68 | 0.72 | DOWN | Beta-D-xylosidase 4 |
| Zm00001d041065 | 23.29 | 10.26 | 16.46 | 7.87 | DOWN | - |
| Zm00001d027555 | 1.47 | 0.64 | 3.05 | 1.41 | DOWN | Pentatricopeptide repeat-containing protein chloroplastic |
| Zm00001d024272 | 1.03 | 0.45 | 1.02 | 0.42 | DOWN | Pentatricopeptide repeat-containing protein mitochondrial |
| Zm00001d018807 | 6.90 | 2.98 | 8.54 | 2.96 | DOWN | Leucine-rich repeat receptor-like serine/threonine-protein kinase |
| Zm00001d023529 | 98.75 | 42.71 | 10.07 | 1.96 | DOWN | abscisic acid stress ripening1 |
| Zm00001d030656 | 4.82 | 2.07 | 9.20 | 4.31 | DOWN | rotten ear1 |
| Zm00001d046749 | 9.73 | 4.18 | 9.57 | 4.03 | DOWN | Calcium-dependent lipid-binding (CaLB domain) plant phosphoribosyltransferase family protein |
| Zm00001d017964 | 0.53 | 0.22 | 1.30 | 0.61 | DOWN | Putative RNA-dependent RNA polymerase |
| Zm00001d047603 | 5.55 | 2.35 | 3.91 | 1.72 | DOWN | L-Aspartase-like family protein |
| Zm00001d053374 | 4.74 | 1.95 | 4.14 | 2.03 | DOWN | N-(5'-phosphoribosyl)anthranilate isomerase |
| Zm00001d019311 | 80.52 | 32.94 | 115.19 | 45.29 | DOWN | IAA-amino acid hydrolase ILR1 |
| Zm00001d038262 | 11.36 | 4.64 | 29.26 | 12.58 | DOWN | Patellin-1 |
| Zm00001d048398 | 2.17 | 0.89 | 3.15 | 1.08 | DOWN | - |
| Zm00001d019145 | 4.24 | 1.71 | 2.41 | 0.73 | DOWN | Leucine-rich repeat receptor-like tyrosine-protein kinase PXC3 |
| Zm00001d011095 | 18.03 | 7.32 | 13.56 | 6.74 | DOWN | Ras-related protein RABA6a |
| Zm00001d025913 | 157.33 | 63.44 | 88.77 | 41.32 | DOWN | Histone H2B |
| Zm00001d054043 | 255.30 | 102.72 | 98.28 | 21.38 | DOWN | Binding protein homolog2 |
| Zm00001d001812 | 2.32 | 0.92 | 3.47 | 1.33 | DOWN | Rubisco Assembly Factor 1 |
| Zm00001d042954 | 18.99 | 7.55 | 16.68 | 6.15 | DOWN | Histone H4 |
| Zm00001d043906 | 6.34 | 2.51 | 4.57 | 2.26 | DOWN | Hydroxyproline-rich glycoprotein family protein |
| Zm00001d010894 | 1.80 | 0.71 | 1.20 | 0.23 | DOWN | Embryonic flower 1-like protein; protein |
| Zm00001d039156 | 15.93 | 6.31 | 15.71 | 7.48 | DOWN | Protein EARLY FLOWERING 3 |
| Zm00001d004448 | 5.12 | 2.03 | 12.70 | 5.60 | DOWN | Probable pectate lyase 8 |
| Zm00001d031316 | 3.63 | 1.43 | 1.71 | 0.71 | DOWN | Phosphatidylinositol/phosphatidylcholine transfer protein SFH13 |
| Zm00001d027511 | 37.89 | 14.94 | 60.35 | 29.01 | DOWN | Catalase isozyme 2 |
| Zm00001d029091 | 54.65 | 21.39 | 92.27 | 22.61 | DOWN | sucrose synthase2 |
| Zm00001d053941 | 4.01 | 1.53 | 2.16 | 0.93 | DOWN | 3-isopropylmalate dehydratase large subunit |
| Zm00001d032718 | 5.27 | 2.03 | 4.94 | 2.33 | DOWN | - |
| Zm00001d032476 | 21.62 | 8.29 | 11.10 | 5.44 | DOWN | Probable methyltransferase PMT18 |
| Zm00001d044895 | 3.66 | 1.41 | 0.94 | 0.15 | DOWN | ATPase%2C coupled to transmembrane movement of substance |
| Zm00001d032728 | 3.16 | 1.21 | 3.87 | 1.44 | DOWN | 3-ketoacyl-CoA synthase |
| Zm00001d004416 | 2.00 | 0.76 | 4.22 | 1.79 | DOWN | Probable RNA-dependent RNA polymerase 5 |
| Zm00001d037932 | 3.21 | 1.21 | 2.02 | 1.00 | DOWN | Transducin/WD40 repeat-like superfamily protein |
| Zm00001d049554 | 20.95 | 7.87 | 10.57 | 2.72 | DOWN | Putative glycerol-3-phosphate transporter 1 |
| Zm00001d002631 | 5.85 | 2.19 | 4.18 | 1.84 | DOWN | Probable methyltransferase PMT13 |
| Zm00001d010314 | 0.67 | 0.24 | 3.60 | 1.64 | DOWN | - |
| Zm00001d007858 | 0.98 | 0.36 | 1.79 | 0.64 | DOWN | Pyridoxal reductase chloroplastic |
| Zm00001d001772 | 3.26 | 1.19 | 23.31 | 3.66 | DOWN | Protein YLS9 |
| Zm00001d002790 | 1.71 | 0.62 | 1.28 | 0.54 | DOWN | GATA transcription factor 26 |
| ENSRNA049479549 | 66.59 | 24.37 | 32.01 | 8.95 | DOWN | 5.8S ribosomal RNA |
| Zm00001d039771 | 23.07 | 8.42 | 44.85 | 18.60 | DOWN | - |
| Zm00001d051014 | 4.37 | 1.59 | 1.82 | 0.89 | DOWN | CMP-sialic acid transporter 3 |
| Zm00001d021422 | 69.93 | 25.39 | 75.74 | 30.88 | DOWN | Histone H4 |
| Zm00001d038227 | 23.26 | 8.39 | 14.72 | 5.90 | DOWN | Putative NAC domain transcription factor superfamily protein |
| Zm00001d051939 | 15.86 | 5.71 | 9.03 | 4.11 | DOWN | Octicosapeptide/Phox/Bem1p family protein |
| Zm00001d032030 | 114.37 | 41.20 | 94.99 | 27.51 | DOWN | Chaperone protein dnaJ 11 chloroplastic |
| Zm00001d031005 | 1.43 | 0.51 | 1.66 | 0.79 | DOWN | Protein NETWORKED 1A |
| Zm00001d018752 | 8.72 | 3.12 | 18.74 | 8.54 | DOWN | Leucine-rich repeat receptor-like serine/threonine-protein kinase BAM1 |
| Zm00001d042706 | 9.27 | 3.31 | 3.43 | 1.37 | DOWN | ARM repeat superfamily protein |
| Zm00001d052955 | 1.04 | 0.37 | 1.11 | 0.26 | DOWN | ARM repeat superfamily protein |
| Zm00001d027422 | 5.04 | 1.79 | 4.03 | 1.94 | DOWN | PsbP-like protein 1 chloroplastic |
| Zm00001d049965 | 4.58 | 1.62 | 3.14 | 0.97 | DOWN | Ribonucleoprotein |
| Zm00001d023718 | 44.00 | 15.55 | 69.36 | 29.29 | DOWN | defective18 |
| Zm00001d026224 | 17.49 | 6.15 | 15.14 | 7.30 | DOWN | - |
| Zm00001d013797 | 4.83 | 1.70 | 5.82 | 2.29 | DOWN | Endoribonuclease Dicer homolog 2 |
| Zm00001d049889 | 29.88 | 10.49 | 75.57 | 19.17 | DOWN | Putative AP2/EREBP transcription factor superfamily protein |
| Zm00001d043511 | 5.92 | 2.07 | 6.74 | 3.16 | DOWN | hexokinase5 |
| Zm00001d043909 | 6.70 | 2.33 | 4.38 | 2.01 | DOWN | Callose synthase 11 |
| Zm00001d018183 | 14.70 | 5.10 | 23.57 | 10.31 | DOWN | mitochondrial phosphate transporter1 |
| Zm00001d028749 | 1.18 | 0.41 | 1.57 | 0.55 | DOWN | Acyl-CoA N-acyltransferase with RING/FYVE/PHD-type zinc finger domain |
| Zm00001d006213 | 205.14 | 70.84 | 152.98 | 48.82 | DOWN | Probable histone H2A.4 |
| Zm00001d010575 | 43.00 | 14.79 | 19.33 | 9.40 | DOWN | Histone H4 |
| Zm00001d041268 | 3.69 | 1.25 | 1.83 | 0.56 | DOWN | - |
| Zm00001d034167 | 2.15 | 0.73 | 1.81 | 0.77 | DOWN | Peptidase/ serine-type peptidase |
| Zm00001d043296 | 11.95 | 4.06 | 8.37 | 2.90 | DOWN | zinc knuckle (CCHC-type) family protein |
| Zm00001d013392 | 54.81 | 18.33 | 41.59 | 15.77 | DOWN | Histone H2A |
| Zm00001d053716 | 8.05 | 2.67 | 9.57 | 4.61 | DOWN | Protein TIC 21 chloroplastic |
| Zm00001d034313 | 9.62 | 3.18 | 3.57 | 1.71 | DOWN | Reticulon-like protein B8 |
| Zm00001d043780 | 13.44 | 4.44 | 16.81 | 7.42 | DOWN | threonine synthase1 |
| Zm00001d047143 | 3.14 | 1.03 | 1.65 | 0.44 | DOWN | Callose synthase 7 |
| Zm00001d012212 | 3.03 | 0.96 | 1.91 | 0.38 | DOWN | gibberellin 20-oxidase5 |
| Zm00001d025407 | 5.93 | 1.85 | 7.24 | 2.77 | DOWN | COP9 signalosome complex subunit 8 |
| Zm00001d047053 | 2.50 | 0.78 | 2.60 | 1.05 | DOWN | BR-signaling kinase 2 |
| Zm00001d015963 | 1.37 | 0.43 | 3.32 | 0.91 | DOWN | Protein DETOXIFICATION 16 |
| Zm00001d017913 | 2.46 | 0.76 | 2.58 | 1.13 | DOWN | Protein NSP-INTERACTING KINASE 1 |
| Zm00001d013063 | 0.90 | 0.28 | 0.40 | 0.02 | DOWN | argonaute5c |
| Zm00001d027868 | 1.76 | 0.54 | 0.42 | 0.18 | DOWN | E3 ubiquitin ligase BIG BROTHER |
| Zm00001d049929 | 17.46 | 5.39 | 15.05 | 6.74 | DOWN | Dihydroxy-acid dehydratase chloroplastic |
| Zm00001d050375 | 58.03 | 17.86 | 35.16 | 17.48 | DOWN | 14-3-3-like protein |
| Zm00001d043422 | 8.95 | 2.72 | 7.88 | 1.37 | DOWN | RING-H2 finger protein ATL2K |
| Zm00001d027450 | 7.79 | 2.37 | 2.80 | 1.34 | DOWN | C-terminal binding protein |
| Zm00001d010044 | 7.64 | 2.31 | 2.67 | 0.94 | DOWN | Triose phosphate/phosphate translocator TPT chloroplastic |
| Zm00001d008679 | 4.30 | 1.30 | 3.69 | 1.67 | DOWN | Nucleotide/sugar transporter family protein |
| Zm00001d026690 | 7.19 | 2.16 | 9.45 | 3.43 | DOWN | Serine/threonine-protein kinase SRK2A |
| Zm00001d039867 | 4.81 | 1.45 | 4.32 | 2.09 | DOWN | Factor of DNA methylation 4 |
| Zm00001d046937 | 26.21 | 7.89 | 26.77 | 11.80 | DOWN | Basic leucine zipper 9 |
| Zm00001d007655 | 1.36 | 0.41 | 0.92 | 0.22 | DOWN | - |
| Zm00001d041214 | 21.05 | 6.33 | 36.13 | 12.21 | DOWN | ATPase 4 plasma membrane-type |
| Zm00001d033497 | 7.37 | 2.21 | 3.04 | 1.31 | DOWN | Plasma-membrane choline transporter family protein |
| Zm00001d009640 | 32.73 | 9.79 | 38.16 | 18.19 | DOWN | Malate dehydrogenase 2 mitochondrial |
| Zm00001d016166 | 34.15 | 10.20 | 32.73 | 13.34 | DOWN | phosphoenolpyruvate carboxylase2 |
| Zm00001d041877 | 2.35 | 0.70 | 4.14 | 1.78 | DOWN | Filament-like plant protein 3 |
| Zm00001d007430 | 2.96 | 0.88 | 10.18 | 3.70 | DOWN | Respiratory burst oxidase homolog protein C |
| Zm00001d036238 | 28.04 | 8.30 | 51.74 | 19.83 | DOWN | GDSL esterase/lipase |
| Zm00001d033088 | 7.13 | 2.11 | 5.77 | 2.30 | DOWN | Whole genome shotgun sequence of line PN40024 scaffold_14.assembly12x (Fragment) |
| Zm00001d052180 | 4.54 | 1.34 | 10.28 | 4.23 | DOWN | tunicate1 |
| Zm00001d016824 | 2.61 | 0.76 | 2.01 | 0.95 | DOWN | methyltransferases;nucleic acid binding |
| Zm00001d039422 | 5.85 | 1.71 | 4.94 | 2.38 | DOWN | phosphoribosylaminoimidazole carboxylase family protein / AIR carboxylase family protein |
| Zm00001d050969 | 10.75 | 3.08 | 7.93 | 3.13 | DOWN | Elongation factor Tu |
| Zm00001d025773 | 145.63 | 41.76 | 176.87 | 38.18 | DOWN | S-adenosylmethionine decarboxylase1 |
| Zm00001d012791 | 2.88 | 0.81 | 3.77 | 1.37 | DOWN | BURP domain protein RD22 |
| Zm00001d053179 | 14.37 | 4.10 | 10.42 | 5.12 | DOWN | Tubby-like F-box protein 10 |
| Zm00001d048703 | 16.08 | 4.49 | 21.88 | 8.60 | DOWN | benzoxazinone synthesis4 |
| Zm00001d045392 | 52.47 | 14.66 | 45.09 | 18.42 | DOWN | Early nodulin 93 |
| Zm00001d048615 | 6.87 | 1.90 | 7.56 | 2.97 | DOWN | Putative membrane lipoprotein |
| Zm00001d011398 | 6.57 | 1.83 | 3.00 | 0.51 | DOWN | - |
| Zm00001d053372 | 7.01 | 1.94 | 4.77 | 2.27 | DOWN | - |
| Zm00001d008588 | 13.01 | 3.60 | 6.42 | 2.64 | DOWN | cytochrome P450 family 77 subfamily A polypeptide 5 pseudogene |
| Zm00001d032213 | 3.42 | 0.95 | 4.76 | 2.06 | DOWN | Transcription factor AIG1 |
| Zm00001d004650 | 4.59 | 1.26 | 3.97 | 1.38 | DOWN | CRP7 - Cysteine-rich family protein precursor expressed |
| Zm00001d038460 | 0.58 | 0.16 | 2.00 | 0.86 | DOWN | Putative aminotransferase class III superfamily protein |
| Zm00001d037631 | 39.41 | 10.79 | 37.70 | 9.11 | DOWN | - |
| Zm00001d050106 | 7.83 | 2.11 | 6.12 | 2.70 | DOWN | ferredoxin-related |
| Zm00001d031651 | 6.94 | 1.87 | 8.98 | 4.29 | DOWN | Mitochondrial import inner membrane translocase subunit Tim8 |
| Zm00001d003659 | 3.98 | 1.06 | 4.11 | 1.85 | DOWN | Serine/threonine-protein kinase SRK2A |
| Zm00001d015231 | 17.41 | 4.66 | 24.94 | 8.45 | DOWN | Glucan endo-1%2C3-beta-glucosidase 6 |
| Zm00001d017606 | 13.68 | 3.64 | 30.91 | 11.27 | DOWN | Transcription repressor OFP6 |
| Zm00001d022525 | 13.54 | 3.59 | 9.51 | 4.11 | DOWN | Dof-type zinc finger DNA-binding family protein |
| Zm00001d043762 | 5.12 | 1.35 | 4.58 | 1.41 | DOWN | ABC transporter B family member 9 |
| Zm00001d046352 | 53.84 | 14.13 | 46.69 | 19.45 | DOWN | Elongation factor 1-gamma 3 |
| Zm00001d015213 | 193.20 | 50.52 | 43.97 | 13.56 | DOWN | leucine-rich repeat/extensin 2 |
| Zm00001d021338 | 6.51 | 1.71 | 7.17 | 2.80 | DOWN | Folate synthesis bifunctional protein |
| Zm00001d035934 | 5.56 | 1.44 | 7.43 | 2.22 | DOWN | - |
| Zm00001d029300 | 6.27 | 1.63 | 0.43 | 0.00 | DOWN | Low temperature viability protein |
| Zm00001d019565 | 42.02 | 10.87 | 17.09 | 4.94 | DOWN | Aquaporin PIP2-6 |
| Zm00001d034892 | 3.50 | 0.89 | 3.54 | 1.65 | DOWN | pentatricopeptide repeat53 |
| Zm00001d011236 | 92.01 | 23.25 | 61.75 | 25.97 | DOWN | Histone H2B |
| Zm00001d026012 | 7.81 | 1.96 | 5.71 | 1.67 | DOWN | OSJNBa0088A01.13 protein; protein |
| Zm00001d049649 | 14.48 | 3.59 | 10.98 | 4.15 | DOWN | succinate dehydrogenase2 |
| Zm00001d026015 | 65.39 | 16.09 | 34.25 | 8.28 | DOWN | Histone H4 |
| Zm00001d050935 | 15.62 | 3.82 | 13.00 | 5.85 | DOWN | Hypoxia induced protein conserved region containing protein |
| Zm00001d002423 | 10.19 | 2.48 | 7.97 | 3.84 | DOWN | Maltose excess protein 1-like |
| Zm00001d038006 | 14.21 | 3.44 | 11.99 | 5.41 | DOWN | Tetratricopeptide repeat (TPR)-like superfamily protein |
| Zm00001d009690 | 12.85 | 3.10 | 10.92 | 5.00 | DOWN | RNA cytidine acetyltransferase 1 |
| Zm00001d037982 | 1.38 | 0.33 | 1.83 | 0.48 | DOWN | AMP deaminase |
| Zm00001d048449 | 67.63 | 16.02 | 38.10 | 10.79 | DOWN | Senescence-associated protein |
| Zm00001d005071 | 0.75 | 0.18 | 3.77 | 1.17 | DOWN | Probable protein phosphatase 2C 65 |
| Zm00001d049400 | 17.91 | 4.21 | 17.81 | 8.71 | DOWN | Transcription factor GTE7 |
| Zm00001d017982 | 0.87 | 0.20 | 0.75 | 0.34 | DOWN | DExH-box ATP-dependent RNA helicase DExH15 chloroplastic |
| Zm00001d039989 | 138.77 | 32.32 | 100.85 | 38.24 | DOWN | - |
| Zm00001d002260 | 9.00 | 2.04 | 4.95 | 2.27 | DOWN | Peroxisomal (S)-2-hydroxy-acid oxidase GLO1 |
| Zm00001d002483 | 3.60 | 0.81 | 2.91 | 0.77 | DOWN | Transducin family protein / WD-40 repeat family protein |
| Zm00001d042016 | 2.33 | 0.52 | 2.81 | 0.64 | DOWN | - |
| Zm00001d050815 | 1.37 | 0.31 | 1.81 | 0.34 | DOWN | F-box/kelch-repeat protein SKIP20 |
| Zm00001d009583 | 4.87 | 1.07 | 7.83 | 2.85 | DOWN | - |
| Zm00001d038682 | 14.60 | 3.25 | 13.11 | 5.62 | DOWN | PLASMODESMATA CALLOSE-BINDING PROTEIN 2 |
| Zm00001d014971 | 0.41 | 0.09 | 0.71 | 0.17 | DOWN | Leucine-rich repeat receptor-like protein kinase PXL1 |
| Zm00001d032322 | 18.71 | 4.14 | 21.53 | 9.91 | DOWN | Nucleobase-ascorbate transporter 6 |
| Zm00001d040466 | 2.57 | 0.57 | 2.51 | 0.89 | DOWN | CO(2)-response secreted protease |
| Zm00001d044290 | 12.65 | 2.79 | 7.37 | 3.24 | DOWN | Beta-galactosidase 1 |
| Zm00001d044202 | 1.11 | 0.24 | 2.42 | 0.79 | DOWN | alpha/beta-Hydrolases superfamily protein |
| Zm00001d052662 | 11.51 | 2.51 | 9.29 | 4.30 | DOWN | C2 domain protein |
| Zm00001d011877 | 4.18 | 0.91 | 3.21 | 1.56 | DOWN | Protein OSB2 chloroplastic |
| Zm00001d011242 | 9.90 | 2.14 | 7.79 | 2.97 | DOWN | porin1 |
| Zm00001d017175 | 6.50 | 1.39 | 6.44 | 2.79 | DOWN | La protein 1 |
| Zm00001d020752 | 41.18 | 8.82 | 23.30 | 10.46 | DOWN | Serine carboxypeptidase-like 40 |
| Zm00001d042044 | 1.69 | 0.36 | 1.51 | 0.62 | DOWN | Cell division protein FtsY homolog chloroplastic |
| Zm00001d041957 | 1.53 | 0.32 | 1.37 | 0.40 | DOWN | Fizzy-related protein |
| Zm00001d012544 | 5.15 | 1.09 | 1.68 | 0.23 | DOWN | myb domain protein 81 |
| Zm00001d037818 | 1.43 | 0.30 | 0.87 | 0.34 | DOWN | phosphoribosylaminoimidazole carboxylase family protein / AIR carboxylase family protein |
| Zm00001d011357 | 6.52 | 1.35 | 6.64 | 2.90 | DOWN | CTP synthase family protein |
| Zm00001d021706 | 40.91 | 8.49 | 5.94 | 1.23 | DOWN | Histone H2A |
| Zm00001d020585 | 78.17 | 16.25 | 46.23 | 10.75 | DOWN | Histone H4 |
| Zm00001d032570 | 1.60 | 0.33 | 3.29 | 1.11 | DOWN | soluble epoxide hydrolase |
| Zm00001d033278 | 7.94 | 1.64 | 5.54 | 1.89 | DOWN | Protein NETWORKED 1A |
| Zm00001d012166 | 2.14 | 0.44 | 1.31 | 0.54 | DOWN | Faciata 1-like protein |
| Zm00001d013168 | 8.19 | 1.66 | 11.35 | 4.36 | DOWN | Probable xyloglucan glycosyltransferase 12 |
| Zm00001d042131 | 2.73 | 0.55 | 1.83 | 0.87 | DOWN | Serine/threonine-protein kinase TIO |
| Zm00001d020544 | 3.05 | 0.61 | 2.15 | 0.39 | DOWN | S-adenosylmethionine decarboxylase proenzyme |
| Zm00001d035285 | 4.50 | 0.90 | 2.88 | 1.30 | DOWN | Heat shock protein 90-6 mitochondrial |
| Zm00001d052051 | 1.61 | 0.32 | 1.64 | 0.56 | DOWN | Protein kinase superfamily protein |
| Zm00001d011771 | 3.86 | 0.77 | 2.60 | 1.22 | DOWN | GATA zinc finger family protein |
| Zm00001d024314 | 4.31 | 0.86 | 0.94 | 0.07 | DOWN | Putative cinnamyl-alcohol dehydrogenase family protein |
| Zm00001d035201 | 149.28 | 29.61 | 97.91 | 41.34 | DOWN | 60S acidic ribosomal protein P0 |
| Zm00001d049179 | 13.04 | 2.57 | 33.27 | 10.31 | DOWN | O-methyltransferase ZRP4 |
| Zm00001d038163 | 283.10 | 55.58 | 215.59 | 102.54 | DOWN | pyruvate orthophosphate dikinase1 |
| Zm00001d003781 | 8.79 | 1.69 | 1.62 | 0.39 | DOWN | Alpha carbonic anhydrase 1 chloroplastic |
| Zm00001d048628 | 26.51 | 4.98 | 15.44 | 7.13 | DOWN | ARM repeat superfamily protein |
| Zm00001d004392 | 9.51 | 1.76 | 7.11 | 3.00 | DOWN | Thylakoid lumenal 15.0 kDa protein 2 chloroplastic |
| Zm00001d033827 | 8.10 | 1.50 | 4.36 | 2.05 | DOWN | chromatin complex subunit A 106 |
| Zm00001d033421 | 4.77 | 0.88 | 2.73 | 1.16 | DOWN | Deoxyuridine 5'-triphosphate nucleotidohydrolase |
| Zm00001d012895 | 1.75 | 0.32 | 3.05 | 1.24 | DOWN | DUF4378 domain protein |
| Zm00001d023309 | 27.22 | 4.98 | 18.39 | 8.48 | DOWN | Sterol 14-demethylase |
| Zm00001d022144 | 3.95 | 0.72 | 2.35 | 0.93 | DOWN | 3-oxoacyl-[acyl-carrier-protein] synthase II chloroplastic |
| Zm00001d020867 | 23.04 | 4.14 | 29.39 | 10.40 | DOWN | Subtilisin-like protease SBT5.3 |
| Zm00001d036351 | 5.76 | 1.03 | 5.61 | 1.46 | DOWN | Putative glycosyl transferase family protein |
| Zm00001d019459 | 8.33 | 1.47 | 3.66 | 1.31 | DOWN | - |
| Zm00001d031929 | 10.38 | 1.84 | 10.93 | 5.19 | DOWN | Dihydroxy-acid dehydratase chloroplastic |
| Zm00001d004512 | 4.21 | 0.74 | 2.65 | 1.00 | DOWN | Transcription factor E2FC |
| Zm00001d014292 | 6.25 | 1.09 | 0.10 | 0.00 | DOWN | Inositol-3-phosphate synthase isozyme 1 |
| Zm00001d027581 | 6.00 | 1.06 | 3.35 | 1.51 | DOWN | GATA zinc finger domain-containing protein 10 |
| Zm00001d012452 | 5.71 | 0.99 | 2.68 | 0.96 | DOWN | Protein kinase superfamily protein |
| Zm00001d047253 | 59.86 | 10.38 | 53.60 | 23.49 | DOWN | sucrose synthase1 |
| Zm00001d020395 | 3.16 | 0.55 | 15.79 | 2.25 | DOWN | Protein CCC1 |
| Zm00001d017331 | 3.84 | 0.66 | 2.97 | 1.20 | DOWN | - |
| Zm00001d034734 | 1.26 | 0.21 | 0.64 | 0.26 | DOWN | Nucleic acid-binding proteins superfamily |
| Zm00001d045554 | 0.42 | 0.07 | 0.30 | 0.06 | DOWN | kinesin-related protein2 |
| Zm00001d024813 | 3.08 | 0.52 | 4.91 | 1.48 | DOWN | Embryonic flower 1-like protein; protein |
| Zm00001d051542 | 1.36 | 0.23 | 0.85 | 0.23 | DOWN | DNA topoisomerase 2 |
| Zm00001d041345 | 0.63 | 0.11 | 1.16 | 0.45 | DOWN | Cyclin-D5-1 |
| Zm00001d003040 | 4.75 | 0.77 | 1.68 | 0.81 | DOWN | ATBRCA1 |
| Zm00001d004709 | 6.96 | 1.14 | 2.31 | 1.12 | DOWN | transcription coactivators |
| Zm00001d053447 | 1.78 | 0.29 | 1.48 | 0.39 | DOWN | CONTAINS InterPro DOMAIN/s |
| Zm00001d002325 | 89.79 | 14.55 | 121.72 | 53.41 | DOWN | - |
| Zm00001d032632 | 7.06 | 1.13 | 5.65 | 2.51 | DOWN | DNA ligase |
| Zm00001d010016 | 51.78 | 8.31 | 33.91 | 15.52 | DOWN | 60S ribosomal protein L11-1 |
| Zm00001d048422 | 4.25 | 0.68 | 5.85 | 1.41 | DOWN | Photosynthetic NDH subunit of subcomplex B 2 chloroplastic |
| Zm00001d040660 | 2.57 | 0.40 | 6.52 | 1.60 | DOWN | Late embryogenesis abundant protein |
| Zm00001d049490 | 3.82 | 0.60 | 3.92 | 1.72 | DOWN | Protein CHLORORESPIRATORY REDUCTION 6 chloroplastic |
| Zm00001d016715 | 3.81 | 0.60 | 5.46 | 2.45 | DOWN | - |
| Zm00001d001857 | 3.40 | 0.52 | 5.34 | 2.01 | DOWN | light harvesting complex photosystem II subunit 6 |
| Zm00001d042090 | 6.69 | 1.02 | 4.24 | 1.84 | DOWN | UMP synthase |
| Zm00001d014852 | 25.08 | 3.87 | 21.45 | 7.13 | DOWN | Ser/Thr-rich protein T10 in DGCR region |
| Zm00001d028669 | 0.48 | 0.07 | 0.36 | 0.08 | DOWN | Kinesin-like protein KIN-5B |
| Zm00001d050753 | 7.27 | 1.11 | 4.61 | 1.82 | DOWN | DDT domain-containing protein |
| Zm00001d044246 | 60.00 | 9.16 | 31.37 | 9.60 | DOWN | Histone H2A |
| Zm00001d000301 | 4.37 | 0.66 | 4.18 | 1.99 | DOWN | exocyst subunit exo70 family protein G1 |
| Zm00001d053143 | 3.49 | 0.52 | 11.26 | 2.93 | DOWN | Amino acid permease 2 |
| Zm00001d041672 | 29.44 | 4.41 | 7.75 | 0.72 | DOWN | Histone H3.2 |
| Zm00001d016564 | 7.37 | 1.09 | 4.46 | 0.95 | DOWN | uncharacterized protein [Source:UniProtKB/TrEMBL;Acc:B6U0E1] |
| Zm00001d014844 | 72.18 | 10.75 | 69.02 | 34.36 | DOWN | 14-alpha-glucan-branching enzyme 2-2 chloroplastic/amyloplastic |
| Zm00001d028512 | 1.50 | 0.22 | 0.47 | 0.12 | DOWN | Phosphatidylinositol 4-phosphate 5-kinase 2 |
| Zm00001d007820 | 2.44 | 0.36 | 2.12 | 0.91 | DOWN | Plant-specific domain TIGR01615 family protein |
| Zm00001d042288 | 127.80 | 18.95 | 79.71 | 35.42 | DOWN | NAC domain-containing protein 68 |
| Zm00001d010180 | 3.37 | 0.50 | 2.53 | 0.66 | DOWN | Thioesterase superfamily protein |
| Zm00001d038453 | 5.74 | 0.83 | 25.59 | 8.94 | DOWN | Putative aminotransferase class III superfamily protein |
| Zm00001d050100 | 99.82 | 14.44 | 26.01 | 12.42 | DOWN | Histone H2B.2 |
| Zm00001d025674 | 7.47 | 1.06 | 7.91 | 3.56 | DOWN | Probably inactive leucine-rich repeat receptor-like protein kinase |
| Zm00001d025567 | 15.03 | 2.16 | 5.17 | 0.44 | DOWN | - |
| Zm00001d052335 | 0.56 | 0.07 | 11.00 | 5.33 | DOWN | Peroxidase 67 |
| Zm00001d039391 | 0.53 | 0.08 | 0.82 | 0.10 | DOWN | - |
| Zm00001d022006 | 10.13 | 1.44 | 8.78 | 3.20 | DOWN | Lipase-like protein |
| Zm00001d053070 | 6.43 | 0.90 | 2.91 | 1.07 | DOWN | DNA polymerase delta catalytic subunit |
| Zm00001d028702 | 4.14 | 0.57 | 3.13 | 1.17 | DOWN | 50S ribosomal protein L10 chloroplastic |
| Zm00001d039299 | 37.20 | 5.16 | 18.94 | 8.23 | DOWN | 40S ribosomal protein S15a-1 |
| Zm00001d053727 | 10.79 | 1.48 | 4.63 | 1.89 | DOWN | Pyrophosphate-energized vacuolar membrane proton pump 1 |
| Zm00001d008996 | 7.86 | 1.08 | 5.12 | 1.67 | DOWN | RNA polymerase I-associated factor PAF67 |
| Zm00001d044211 | 12.92 | 1.77 | 7.95 | 1.60 | DOWN | Phosphoglucomutase chloroplastic |
| Zm00001d029201 | 1.88 | 0.26 | 2.91 | 0.82 | DOWN | 50S ribosomal protein L6 |
| Zm00001d042528 | 2.83 | 0.38 | 1.32 | 0.53 | DOWN | Chromatin assembly factor 1 subunit FAS1 |
| Zm00001d038923 | 173.54 | 23.36 | 115.46 | 44.54 | DOWN | Guanine nucleotide-binding protein beta subunit-like protein |
| Zm00001d053876 | 0.54 | 0.07 | 1.56 | 0.64 | DOWN | Protein kinase protein with adenine nucleotide alpha hydrolases-like domain |
| Zm00001d038054 | 1.72 | 0.23 | 1.00 | 0.45 | DOWN | N-acetyltransferases;N-acetyltransferases |
| Zm00001d037498 | 967.49 | 128.06 | 1070.73 | 430.60 | DOWN | L-tryptophan--pyruvate aminotransferase 1 |
| Zm00001d053127 | 4.02 | 0.52 | 5.99 | 2.66 | DOWN | Long chain acyl-CoA synthetase 2 |
| Zm00001d022461 | 77.15 | 10.03 | 88.01 | 19.30 | DOWN | ERF-like protein |
| Zm00001d046538 | 1.41 | 0.18 | 0.72 | 0.23 | DOWN | Sec14p-like phosphatidylinositol transfer family protein |
| Zm00001d010416 | 2.87 | 0.36 | 3.20 | 0.72 | DOWN | Transcription factor bHLH133 |
| Zm00001d011661 | 36.06 | 4.58 | 30.76 | 8.85 | DOWN | GDSL esterase/lipase APG |
| Zm00001d039316 | 43.81 | 5.57 | 32.62 | 16.07 | DOWN | Reticulon-like protein B4 |
| Zm00001d037493 | 10.97 | 1.37 | 3.18 | 1.34 | DOWN | Heparanase-like protein 3 |
| Zm00001d021469 | 0.43 | 0.05 | 0.48 | 0.16 | DOWN | Glutathione S-transferase F9 |
| Zm00001d007382 | 10.08 | 1.23 | 5.37 | 1.48 | DOWN | Transcription factor ICE1 |
| Zm00001d016895 | 79.03 | 9.49 | 46.61 | 22.01 | DOWN | Gibberellin receptor GID1L2 |
| Zm00001d053659 | 3.25 | 0.39 | 2.68 | 1.11 | DOWN | Calcium ion binding protein |
| Zm00001d014180 | 0.59 | 0.07 | 0.66 | 0.23 | DOWN | Flowering locus K homology domain |
| Zm00001d021300 | 55.37 | 6.53 | 7.01 | 1.92 | DOWN | Histone H2A |
| Zm00001d037890 | 1.39 | 0.17 | 0.60 | 0.10 | DOWN | Subtilisin-like protease SBT1.9 |
| Zm00001d050664 | 5.19 | 0.60 | 2.10 | 0.88 | DOWN | Transcription factor E2FB |
| Zm00001d000709 | 10.90 | 1.25 | 5.19 | 1.43 | DOWN | - |
| Zm00001d007657 | 23.27 | 2.64 | 32.76 | 9.87 | DOWN | sugary-enhancer1 |
| Zm00001d019045 | 81.39 | 9.13 | 52.25 | 12.42 | DOWN | Histone H2A |
| Zm00001d022396 | 1.90 | 0.21 | 0.80 | 0.36 | DOWN | binding |
| Zm00001d036392 | 1.58 | 0.18 | 5.91 | 1.05 | DOWN | BZIP transcription factor TRAB1 |
| Zm00001d016256 | 1.42 | 0.16 | 2.71 | 0.48 | DOWN | Meiosis 5 |
| Zm00001d009415 | 30.34 | 3.36 | 60.14 | 22.81 | DOWN | Arabinogalactan peptide 3 |
| Zm00001d039062 | 6.24 | 0.69 | 1.97 | 0.62 | DOWN | Putative timeless C-term domain and Homeodomain protein |
| Zm00001d039293 | 1.32 | 0.14 | 17.48 | 7.70 | DOWN | tonoplast intrinsic protein4 |
| Zm00001d038358 | 9.25 | 1.02 | 4.86 | 1.64 | DOWN | Delta-1-pyrroline-5-carboxylate synthase B |
| Zm00001d021216 | 5.85 | 0.64 | 5.26 | 0.88 | DOWN | Protein DETOXIFICATION 50 |
| Zm00001d013746 | 36.78 | 3.94 | 46.37 | 10.34 | DOWN | - |
| Zm00001d046941 | 0.79 | 0.08 | 1.19 | 0.25 | DOWN | Receptor-like cytosolic serine/threonine-protein kinase RBK1 |
| Zm00001d039331 | 2.22 | 0.23 | 1.91 | 0.66 | DOWN | V-type proton ATPase subunit E |
| Zm00001d050697 | 37.55 | 3.90 | 20.87 | 5.28 | DOWN | Histone H3.2 |
| Zm00001d016723 | 63.04 | 6.49 | 38.91 | 5.36 | DOWN | Histone H3 |
| Zm00001d041215 | 2.14 | 0.22 | 1.93 | 0.64 | DOWN | ATP binding protein |
| Zm00001d037307 | 31.54 | 3.23 | 31.92 | 12.92 | DOWN | NHL domain-containing protein |
| Zm00001d047787 | 77.29 | 7.79 | 90.98 | 13.40 | DOWN | Histone H2A |
| Zm00001d013066 | 23.03 | 2.31 | 53.07 | 3.05 | DOWN | Histone H1 |
| Zm00001d029078 | 3.27 | 0.32 | 3.09 | 0.14 | DOWN | DNA binding protein [Source:UniProtKB/TrEMBL;Acc:B4FLH1] |
| Zm00001d043574 | 74.08 | 7.33 | 73.07 | 20.92 | DOWN | - |
| Zm00001d008982 | 27.50 | 2.72 | 14.91 | 6.45 | DOWN | Reticulon-like protein B4 |
| Zm00001d017514 | 1.68 | 0.17 | 1.10 | 0.47 | DOWN | BEACH domain-containing protein C2 |
| Zm00001d009888 | 17.00 | 1.61 | 3.72 | 1.69 | DOWN | DNA primase |
| Zm00001d014498 | 1.31 | 0.12 | 2.74 | 0.34 | DOWN | stearoyl-acyl-carrier-protein desaturase7 |
| Zm00001d022028 | 4.29 | 0.40 | 1.75 | 0.78 | DOWN | MutS homolog1 |
| Zm00001d016505 | 149.03 | 13.88 | 112.86 | 52.76 | DOWN | F-box protein |
| Zm00001d038583 | 4.98 | 0.46 | 3.32 | 1.52 | DOWN | Structural maintenance of chromosomes protein |
| Zm00001d048813 | 432.72 | 39.59 | 339.24 | 85.31 | DOWN | Zein-alpha ZA1/M1 |
| Zm00001d013067 | 56.65 | 5.18 | 105.61 | 10.47 | DOWN | Histone H1 |
| Zm00001d021583 | 0.51 | 0.05 | 0.26 | 0.05 | DOWN | Protein TPX2 |
| Zm00001d025670 | 0.29 | 0.03 | 0.78 | 0.12 | DOWN | Glycerophosphodiester phosphodiesterase GDPDL3 |
| Zm00001d048818 | 6437.59 | 574.27 | 185.33 | 36.39 | DOWN | - |
| Zm00001d025889 | 0.22 | 0.02 | 1.69 | 0.23 | DOWN | Probable metal-nicotianamine transporter YSL7 |
| Zm00001d044418 | 2.02 | 0.17 | 0.91 | 0.28 | DOWN | Ubiquitin-conjugating enzyme family protein |
| Zm00001d012128 | 1.96 | 0.17 | 3.12 | 1.03 | DOWN | Putative two-component response regulator family protein |
| Zm00001d045268 | 50.57 | 4.33 | 37.46 | 13.04 | DOWN | Histone H3.2 |
| Zm00001d020008 | 0.86 | 0.07 | 0.35 | 0.01 | DOWN | Indole-3-glycerol phosphate synthase chloroplastic |
| Zm00001d049856 | 3.12 | 0.26 | 0.77 | 0.18 | DOWN | Serine/threonine-protein kinase CDG1 |
| Zm00001d013873 | 91.89 | 7.49 | 19.10 | 5.45 | DOWN | Actin-2 |
| Zm00001d045940 | 3.47 | 0.28 | 1.71 | 0.53 | DOWN | - |
| Zm00001d036322 | 2.70 | 0.22 | 2.01 | 0.28 | DOWN | Ribonucleoside-diphosphate reductase |
| Zm00001d018087 | 22.33 | 1.80 | 15.97 | 5.71 | DOWN | - |
| Zm00001d011759 | 7.15 | 0.56 | 2.70 | 0.82 | DOWN | Probable alphaalpha-trehalose-phosphate synthase [UDP-forming] 7 |
| Zm00001d003718 | 0.53 | 0.04 | 2.95 | 0.96 | DOWN | - |
| Zm00001d043164 | 0.28 | 0.02 | 0.42 | 0.03 | DOWN | Cyclin-B1-4 |
| Zm00001d027295 | 19.91 | 1.54 | 16.18 | 7.14 | DOWN | beta tubulin1 |
| Zm00001d043361 | 3.55 | 0.28 | 2.84 | 0.61 | DOWN | PLASMODESMATA CALLOSE-BINDING PROTEIN 2 |
| Zm00001d047274 | 0.18 | 0.01 | 5.03 | 0.85 | DOWN | Expressed protein; protein |
| Zm00001d046891 | 83.75 | 6.39 | 26.83 | 12.06 | DOWN | Methionyl-tRNA synthetase |
| Zm00001d009088 | 6.36 | 0.48 | 5.28 | 1.44 | DOWN | myb transcription factor95 |
| Zm00001d009210 | 4.75 | 0.36 | 4.71 | 2.14 | DOWN | DnaJ protein ERDJ3B |
| Zm00001d048634 | 26.44 | 1.95 | 12.28 | 1.95 | DOWN | DIBOA-glucoside dioxygenase BX6 |
| Zm00001d002874 | 5.81 | 0.42 | 9.67 | 3.20 | DOWN | OSJNBa0038O10.24 protein; protein |
| Zm00001d044293 | 3.46 | 0.25 | 3.28 | 0.83 | DOWN | Protein kinase protein with adenine nucleotide alpha hydrolases-like domain |
| Zm00001d034062 | 64.52 | 4.61 | 42.48 | 16.23 | DOWN | PLASMODESMATA CALLOSE-BINDING PROTEIN 2 |
| Zm00001d045844 | 0.24 | 0.02 | 7.20 | 2.86 | DOWN | - |
| Zm00001d025406 | 40.17 | 2.76 | 15.78 | 3.47 | DOWN | Histone H3.2 |
| Zm00001d048491 | 84.06 | 5.80 | 35.95 | 11.13 | DOWN | Histone H2B |
| Zm00001d045649 | 60.94 | 4.16 | 14.54 | 6.86 | DOWN | Ribonucleoside-diphosphate reductase small chain |
| Zm00001d009646 | 75.54 | 5.08 | 60.62 | 21.24 | DOWN | scarecrow-like1 |
| Zm00001d041414 | 3.75 | 0.24 | 2.22 | 0.44 | DOWN | F-box/LRR-repeat protein 17 |
| Zm00001d034163 | 5.34 | 0.34 | 13.83 | 1.42 | DOWN | Dof zinc finger protein DOF5.7 |
| Zm00001d021477 | 27.92 | 1.75 | 15.72 | 2.63 | DOWN | Histone H4 |
| Zm00001d041596 | 9.42 | 0.59 | 7.76 | 2.16 | DOWN | Probable glycerol-3-phosphate acyltransferase 2 |
| Zm00001d008403 | 442.25 | 27.34 | 926.62 | 449.96 | DOWN | Arabidopsis NAC domain containing protein 87 |
| Zm00001d020984 | 122.83 | 7.42 | 126.97 | 29.66 | DOWN | Probable sarcosine oxidase |
| Zm00001d040702 | 4.89 | 0.30 | 10.02 | 1.60 | DOWN | guaiacol peroxidase1 |
| Zm00001d049508 | 1.16 | 0.07 | 7.56 | 2.22 | DOWN | Mediator of RNA polymerase II transcription subunit 36a |
| Zm00001d013786 | 47.19 | 2.81 | 52.71 | 17.72 | DOWN | transferases;folic acid binding |
| Zm00001d028423 | 1.26 | 0.07 | 3.67 | 0.77 | DOWN | IRK-interacting protein |
| Zm00001d022444 | 7.72 | 0.45 | 12.33 | 4.64 | DOWN | PAP/OAS1 substrate-binding domain superfamily |
| Zm00001d010730 | 24.95 | 1.44 | 8.04 | 2.91 | DOWN | Transcription factor TCP5 |
| Zm00001d039790 | 36.21 | 2.08 | 20.70 | 4.75 | DOWN | Histone H3.2 |
| Zm00001d036250 | 21.30 | 1.25 | 6.43 | 0.97 | DOWN | Histone H3-like 5 |
| Zm00001d051591 | 52.38 | 2.97 | 28.51 | 5.00 | DOWN | histone 2B5 |
| Zm00001d003164 | 26.99 | 1.51 | 9.15 | 4.40 | DOWN | Ribonucleoside-diphosphate reductase small chain C |
| Zm00001d042730 | 84.74 | 4.70 | 42.41 | 8.69 | DOWN | Histone H3 |
| Zm00001d038467 | 18.68 | 1.01 | 6.45 | 2.73 | DOWN | DNA replication licensing factor MCM3 homolog 2 |
| Zm00001d003730 | 36.85 | 1.98 | 13.85 | 4.39 | DOWN | Histone H3.2 |
| Zm00001d006738 | 3.29 | 0.18 | 1.19 | 0.48 | DOWN | Cytoplasmic protein of eukaryotic origin (38.3 kD) |
| Zm00001d047489 | 8.98 | 0.49 | 13.17 | 4.28 | DOWN | - |
| Zm00001d043959 | 2.93 | 0.15 | 5.34 | 1.62 | DOWN | Protein kinase superfamily protein |
| Zm00001d028210 | 5.48 | 0.29 | 2.42 | 0.93 | DOWN | Replication protein A 70 kDa DNA-binding subunit B |
| Zm00001d018839 | 6.72 | 0.36 | 4.28 | 0.74 | DOWN | cytochrome P-450 19 |
| Zm00001d010918 | 9.32 | 0.48 | 18.39 | 8.10 | DOWN | Plant IF-like protein |
| Zm00001d023801 | 2.71 | 0.14 | 0.82 | 0.36 | DOWN | DNA polymerase alpha 2 |
| Zm00001d037436 | 51.68 | 2.64 | 1886.55 | 356.34 | DOWN | 18kD delta zein [Source:UniProtKB/TrEMBL;Acc:Q946V9] |
| Zm00001d023453 | 8.92 | 0.44 | 18.91 | 5.17 | DOWN | - |
| Zm00001d021433 | 7.82 | 0.38 | 3.31 | 1.30 | DOWN | High mobility group B protein 2 |
| Zm00001d046489 | 0.90 | 0.05 | 3.09 | 0.90 | DOWN | - |
| Zm00001d024094 | 2.19 | 0.10 | 0.74 | 0.26 | DOWN | Sister chromatid cohesion 1 protein 3 |
| Zm00001d048808 | 626.78 | 28.81 | 400.80 | 115.33 | DOWN | Kafirin PSKR2 Precursor |
| Zm00001d012837 | 69.01 | 2.97 | 24.51 | 3.09 | DOWN | Histone H2A |
| Zm00001d002789 | 1.38 | 0.06 | 0.70 | 0.20 | DOWN | Pentatricopeptide repeat-containing protein |
| Zm00001d002546 | 26.52 | 1.11 | 8.96 | 2.07 | DOWN | Histone H4 |
| Zm00001d031751 | 1.89 | 0.08 | 0.87 | 0.29 | DOWN | DNA polymerase epsilon subunit B |
| Zm00001d049619 | 53.08 | 2.20 | 31.39 | 13.34 | DOWN | 60S acidic ribosomal protein P0-2 |
| Zm00001d035023 | 21.72 | 0.89 | 10.37 | 4.32 | DOWN | Chromatin assembly factor 1 subunit FAS2 |
| Zm00001d032398 | 0.31 | 0.01 | 1.34 | 0.31 | DOWN | Plant mobile domain protein family |
| Zm00001d035760 | 14632.82 | 589.69 | 39700.49 | 16409.36 | DOWN | zein protein 15kDa15 |
| Zm00001d047392 | 5.50 | 0.22 | 3.52 | 1.70 | DOWN | Transducin/WD40 repeat-like superfamily protein |
| Zm00001d028452 | 57.86 | 2.25 | 75.82 | 21.24 | DOWN | Glyoxal oxidase |
| Zm00001d035764 | 8.59 | 0.33 | 4.28 | 1.45 | DOWN | Putative RING finger U-box domain family protein |
| Zm00001d033822 | 14.15 | 0.55 | 3.87 | 0.86 | DOWN | Probable histone H2AXa |
| Zm00001d017286 | 32.83 | 1.26 | 7.35 | 2.15 | DOWN | - |
| Zm00001d012671 | 5.44 | 0.21 | 1.66 | 0.58 | DOWN | Cytidine/deoxycytidylate deaminase family protein |
| Zm00001d010321 | 660.25 | 24.66 | 641.88 | 91.63 | DOWN | pyruvate orthophosphate dikinase2 |
| Zm00001d048232 | 1.08 | 0.04 | 0.61 | 0.05 | DOWN | Purine permease 3 |
| Zm00001d042875 | 28.29 | 0.97 | 21.62 | 5.11 | DOWN | - |
| Zm00001d028409 | 2.00 | 0.07 | 0.73 | 0.08 | DOWN | Chaperonin 60 subunit beta 4 chloroplastic |
| Zm00001d014084 | 8.36 | 0.28 | 3.09 | 1.13 | DOWN | Zinc finger (C3HC4-type RING finger) family protein |
| Zm00001d023692 | 7.86 | 0.25 | 2.35 | 0.75 | DOWN | selenium binding |
| Zm00001d010877 | 1.35 | 0.04 | 0.57 | 0.11 | DOWN | BRCT domain-containing DNA repair protein |
| Zm00001d048810 | 335.90 | 10.60 | 170.40 | 21.92 | DOWN | - |
| Zm00001d052749 | 23.73 | 0.73 | 7.80 | 3.46 | DOWN | nucleosome/chromatin assembly factor D |
| Zm00001d008393 | 1.05 | 0.03 | 0.73 | 0.21 | DOWN | Putative aldehyde dehydrogenase family protein |
| Zm00001d032504 | 4.55 | 0.11 | 2.40 | 0.26 | DOWN | Monocopper oxidase-like protein SKU5 |
| Zm00001d022226 | 11.37 | 0.29 | 4.57 | 1.64 | DOWN | Origin recognition complex subunit 6 |
| Zm00001d045919 | 33.63 | 0.85 | 4.96 | 1.68 | DOWN | Pyrophosphate--fructose 6-phosphate 1-phosphotransferase subunit alpha 2 |
| Zm00001d023462 | 7.12 | 0.18 | 1.58 | 0.45 | DOWN | Probable DNA primase large subunit |
| Zm00001d048807 | 31.37 | 0.75 | 34.44 | 12.43 | DOWN | - |
| Zm00001d010406 | 6.66 | 0.14 | 2.22 | 0.55 | DOWN | Minichromosome maintenance protein |
| Zm00001d048366 | 0.68 | 0.01 | 1.20 | 0.30 | DOWN | Expressed protein; protein |
| Zm00001d014141 | 8.40 | 0.18 | 2.66 | 0.83 | DOWN | F-box protein |
| Zm00001d030614 | 16.35 | 0.33 | 3.37 | 1.40 | DOWN | DNA replication licensing factor MCM7 |
| Zm00001d052021 | 1.48 | 0.03 | 1.36 | 0.28 | DOWN | DNA replication licensing factor MCM5 |
| Zm00001d039718 | 26.29 | 0.52 | 8.45 | 3.45 | DOWN | Mini-chromosome maintenance complex-binding protein |
| Zm00001d018531 | 20.31 | 0.33 | 2.51 | 0.90 | DOWN | Replication protein A 32 kDa subunit A |
| Zm00001d035963 | 27.20 | 0.43 | 6.42 | 1.43 | DOWN | - |
| Zm00001d046995 | 57.77 | 0.93 | 74.34 | 10.81 | DOWN | Patellin-5 |
| Zm00001d029647 | 0.82 | 0.01 | 0.44 | 0.07 | DOWN | - |
| Zm00001d033365 | 18.27 | 0.27 | 8.35 | 2.49 | DOWN | Putative cyclin-A3-1 |
| Zm00001d044540 | 28.55 | 0.39 | 9.63 | 2.02 | DOWN | DNA replication licensing factor MCM4 |
| Zm00001d049190 | 17.52 | 0.22 | 3.90 | 1.03 | DOWN | DNA replication licensing factor MCM2 |
| Zm00001d009292 | 1.83 | 0.00 | 78.21 | 2.64 | DOWN | PLATZ transcription factor family protein |
| Zm00001d045937 | 621.37 | 3.90 | 11354.53 | 992.41 | DOWN | 10 kD zein protein [Source:UniProtKB/TrEMBL;Acc:Q41881] |
| Zm00001d018624 | 0.61 | 0.00 | 0.51 | 0.14 | DOWN | kinesin-related protein11 |
| Zm00001d019852 | 3.45 | 0.02 | 0.52 | 0.13 | DOWN | transducin family protein / WD-40 repeat family protein |
| Zm00001d022443 | 8.46 | 0.03 | 8.51 | 1.23 | DOWN | F-box domain containing protein-like |
| Zm00001d045354 | 12.32 | 0.04 | 3.21 | 0.56 | DOWN | CDT1-like protein a chloroplastic |
| Zm00001d053824 | 26.92 | 0.06 | 2.01 | 0.08 | DOWN | - |

**Supplementary Table 3-1** Detail information of the 90 and 29 KEGG pathways enriched by the 770 common up- and down-regulated genes

| Pathway ID | Pathway | Heat stress response genes finded out in the pathway (182) | All genes in the Pathway (7940) | *P-value* | *Q-value* |
| --- | --- | --- | --- | --- | --- |
| ko04141 | Protein processing in endoplasmic reticulum | 27 | 376 | 1.01E-07 | 9.09E-06 |
| ko03030 | DNA replication | 13 | 105 | 6.96E-07 | 3.13E-05 |
| ko00402 | Benzoxazinoid biosynthesis | 3 | 14 | 0.003579 | 8.87E-02 |
| ko00240 | Pyrimidine metabolism | 7 | 88 | 0.003942 | 8.87E-02 |
| ko00500 | Starch and sucrose metabolism | 12 | 238 | 0.008465 | 1.52E-01 |
| ko00230 | Purine metabolism | 9 | 166 | 0.0139419 | 2.09E-01 |
| ko00290 | Valine, leucine and isoleucine biosynthesis | 3 | 28 | 0.0254849 | 3.28E-01 |
| ko03430 | Mismatch repair | 5 | 83 | 0.0414287 | 4.59E-01 |
| ko00940 | Phenylpropanoid biosynthesis | 11 | 270 | 0.0458786 | 4.59E-01 |
| ko00591 | Linoleic acid metabolism | 2 | 19 | 0.0692438 | 5.31E-01 |
| ko00620 | Pyruvate metabolism | 6 | 129 | 0.0756771 | 5.31E-01 |
| ko00061 | Fatty acid biosynthesis | 4 | 70 | 0.0763291 | 5.31E-01 |
| ko00052 | Galactose metabolism | 5 | 99 | 0.0766565 | 5.31E-01 |
| ko03440 | Homologous recombination | 5 | 104 | 0.0901913 | 5.80E-01 |
| ko03420 | Nucleotide excision repair | 5 | 109 | 0.1048809 | 5.97E-01 |
| ko00330 | Arginine and proline metabolism | 4 | 79 | 0.1072277 | 5.97E-01 |
| ko00780 | Biotin metabolism | 2 | 27 | 0.1264248 | 5.97E-01 |
| ko00380 | Tryptophan metabolism | 4 | 84 | 0.1264847 | 5.97E-01 |
| ko00270 | Cysteine and methionine metabolism | 6 | 151 | 0.133015 | 5.97E-01 |
| ko01110 | Biosynthesis of secondary metabolites | 50 | 1901 | 0.1490613 | 5.97E-01 |
| ko04712 | Circadian rhythm - plant | 4 | 91 | 0.1556662 | 5.97E-01 |
| ko00904 | Diterpenoid biosynthesis | 2 | 32 | 0.1662055 | 5.97E-01 |
| ko00903 | Limonene and pinene degradation | 1 | 8 | 0.1693879 | 5.97E-01 |
| ko00944 | Flavone and flavonol biosynthesis | 1 | 8 | 0.1693879 | 5.97E-01 |
| ko00770 | Pantothenate and CoA biosynthesis | 2 | 33 | 0.1744076 | 5.97E-01 |
| ko00460 | Cyanoamino acid metabolism | 3 | 63 | 0.1750707 | 5.97E-01 |
| ko03410 | Base excision repair | 3 | 65 | 0.1865717 | 5.97E-01 |
| ko04626 | Plant-pathogen interaction | 10 | 316 | 0.1888288 | 5.97E-01 |
| ko00280 | Valine, leucine and isoleucine degradation | 3 | 66 | 0.1923954 | 5.97E-01 |
| ko00051 | Fructose and mannose metabolism | 4 | 103 | 0.2106983 | 6.14E-01 |
| ko01100 | Metabolic pathways | 87 | 3543 | 0.2122956 | 6.14E-01 |
| ko00660 | C5-Branched dibasic acid metabolism | 1 | 11 | 0.2252682 | 6.14E-01 |
| ko00966 | Glucosinolate biosynthesis | 1 | 11 | 0.2252682 | 6.14E-01 |
| ko00710 | Carbon fixation in photosynthetic organisms | 4 | 111 | 0.2500455 | 6.33E-01 |
| ko01212 | Fatty acid metabolism | 4 | 111 | 0.2500455 | 6.33E-01 |
| ko04146 | Peroxisome | 5 | 149 | 0.2563053 | 6.33E-01 |
| ko00260 | Glycine, serine and threonine metabolism | 4 | 113 | 0.2601268 | 6.33E-01 |
| ko00062 | Fatty acid elongation | 2 | 45 | 0.2759495 | 6.54E-01 |
| ko03060 | Protein export | 3 | 83 | 0.2964644 | 6.84E-01 |
| ko00480 | Glutathione metabolism | 5 | 164 | 0.3231314 | 7.16E-01 |
| ko00750 | Vitamin B6 metabolism | 1 | 17 | 0.3260574 | 7.16E-01 |
| ko00520 | Amino sugar and nucleotide sugar metabolism | 6 | 218 | 0.3834194 | 8.22E-01 |
| ko01210 | 2-Oxocarboxylic acid metabolism | 3 | 99 | 0.397036 | 8.28E-01 |
| ko00250 | Alanine, aspartate and glutamate metabolism | 3 | 101 | 0.4094265 | 8.28E-01 |
| ko00511 | Other glycan degradation | 1 | 23 | 0.413797 | 8.28E-01 |
| ko00340 | Histidine metabolism | 1 | 25 | 0.4404408 | 8.35E-01 |
| ko00071 | Fatty acid degradation | 2 | 66 | 0.4491354 | 8.35E-01 |
| ko00531 | Glycosaminoglycan degradation | 1 | 26 | 0.4533074 | 8.35E-01 |
| ko00400 | Phenylalanine, tyrosine and tryptophan biosynthesis | 2 | 69 | 0.4721049 | 8.35E-01 |
| ko00010 | Glycolysis / Gluconeogenesis | 5 | 199 | 0.4820493 | 8.35E-01 |
| ko00196 | Photosynthesis - antenna proteins | 1 | 29 | 0.4901685 | 8.35E-01 |
| ko00630 | Glyoxylate and dicarboxylate metabolism | 3 | 115 | 0.4935377 | 8.35E-01 |
| ko00310 | Lysine degradation | 2 | 73 | 0.5018234 | 8.35E-01 |
| ko00040 | Pentose and glucuronate interconversions | 3 | 118 | 0.5108116 | 8.35E-01 |
| ko00941 | Flavonoid biosynthesis | 2 | 76 | 0.5233994 | 8.35E-01 |
| ko00740 | Riboflavin metabolism | 1 | 32 | 0.5245569 | 8.35E-01 |
| ko00563 | Glycosylphosphatidylinositol (GPI)-anchor biosynthesis | 1 | 34 | 0.5461907 | 8.35E-01 |
| ko01200 | Carbon metabolism | 9 | 392 | 0.5471952 | 8.35E-01 |
| ko00562 | Inositol phosphate metabolism | 3 | 125 | 0.5498808 | 8.35E-01 |
| ko00030 | Pentose phosphate pathway | 2 | 82 | 0.5646432 | 8.35E-01 |
| ko00053 | Ascorbate and aldarate metabolism | 2 | 83 | 0.5712649 | 8.35E-01 |
| ko00195 | Photosynthesis | 2 | 85 | 0.5842897 | 8.35E-01 |
| ko00945 | Stilbenoid, diarylheptanoid and gingerol biosynthesis | 1 | 38 | 0.5865648 | 8.35E-01 |
| ko00790 | Folate biosynthesis | 1 | 40 | 0.6053913 | 8.35E-01 |
| ko01040 | Biosynthesis of unsaturated fatty acids | 1 | 40 | 0.6053913 | 8.35E-01 |
| ko00561 | Glycerolipid metabolism | 3 | 137 | 0.6124189 | 8.35E-01 |
| ko00360 | Phenylalanine metabolism | 1 | 44 | 0.6405242 | 8.60E-01 |
| ko04016 | MAPK signaling pathway - plant | 6 | 290 | 0.6589855 | 8.72E-01 |
| ko00100 | Steroid biosynthesis | 1 | 48 | 0.6725449 | 8.77E-01 |
| ko00908 | Zeatin biosynthesis | 1 | 51 | 0.6946838 | 8.93E-01 |
| ko02010 | ABC transporters | 1 | 53 | 0.7086095 | 8.98E-01 |
| ko00020 | Citrate cycle (TCA cycle) | 2 | 113 | 0.7364828 | 9.08E-01 |
| ko00410 | beta-Alanine metabolism | 1 | 61 | 0.7582729 | 9.08E-01 |
| ko01230 | Biosynthesis of amino acids | 7 | 377 | 0.7682591 | 9.08E-01 |
| ko00910 | Nitrogen metabolism | 1 | 65 | 0.7798498 | 9.08E-01 |
| ko00130 | Ubiquinone and other terpenoid-quinone biosynthesis | 1 | 67 | 0.7899087 | 9.08E-01 |
| ko04130 | SNARE interactions in vesicular transport | 1 | 67 | 0.7899087 | 9.08E-01 |
| ko04070 | Phosphatidylinositol signaling system | 2 | 132 | 0.8105573 | 9.08E-01 |
| ko03008 | Ribosome biogenesis in eukaryotes | 2 | 140 | 0.8357975 | 9.08E-01 |
| ko04145 | Phagosome | 2 | 141 | 0.8387307 | 9.08E-01 |
| ko03010 | Ribosome | 9 | 512 | 0.8387733 | 9.08E-01 |
| ko00970 | Aminoacyl-tRNA biosynthesis | 1 | 80 | 0.8450258 | 9.08E-01 |
| ko03040 | Spliceosome | 5 | 311 | 0.8468694 | 9.08E-01 |
| ko00190 | Oxidative phosphorylation | 3 | 203 | 0.8495863 | 9.08E-01 |
| ko04144 | Endocytosis | 5 | 319 | 0.8619779 | 9.08E-01 |
| ko04120 | Ubiquitin mediated proteolysis | 3 | 212 | 0.8695048 | 9.08E-01 |
| ko00600 | Sphingolipid metabolism | 1 | 90 | 0.8774097 | 9.08E-01 |
| ko00564 | Glycerophospholipid metabolism | 2 | 174 | 0.9125 | 9.33E-01 |
| ko04075 | Plant hormone signal transduction | 6 | 424 | 0.9292871 | 9.40E-01 |
| ko03018 | RNA degradation | 2 | 228 | 0.9695495 | 9.70E-01 |

**Supplementary Table 3-2** Detail information of the 45 differentially regulated genes, respectively.

| Pathway ID | Pathway | Heat stress response genes finded out in the pathway (182) | All genes in the Pathway (7940) | *P-value* | *Q-value* |
| --- | --- | --- | --- | --- | --- |
| ko03030 | DNA replication | 8 | 105 | 9.165E-10 | 2.658E-08 |
| ko04141 | Protein processing in endoplasmic reticulum | 5 | 376 | 0.006692 | 0.0970336 |
| ko03410 | Base excision repair | 2 | 65 | 0.0189001 | 0.1730232 |
| ko00944 | Flavone and flavonol biosynthesis | 1 | 8 | 0.0259095 | 0.1730232 |
| ko03430 | Mismatch repair | 2 | 83 | 0.0298316 | 0.1730232 |
| ko03420 | Nucleotide excision repair | 2 | 109 | 0.0490014 | 0.2368401 |
| ko00591 | Linoleic acid metabolism | 1 | 19 | 0.0604832 | 0.2505733 |
| ko00531 | Glycosaminoglycan degradation | 1 | 26 | 0.081867 | 0.2895355 |
| ko00904 | Diterpenoid biosynthesis | 1 | 32 | 0.0998228 | 0.2895355 |
| ko00480 | Glutathione metabolism | 2 | 164 | 0.0998398 | 0.2895355 |
| ko00360 | Phenylalanine metabolism | 1 | 44 | 0.1347267 | 0.353001 |
| ko00100 | Steroid biosynthesis | 1 | 48 | 0.1460694 | 0.353001 |
| ko00280 | Valine, leucine and isoleucine degradation | 1 | 66 | 0.1953662 | 0.4358169 |
| ko00941 | Flavonoid biosynthesis | 1 | 76 | 0.2215587 | 0.458943 |
| ko00240 | Pyrimidine metabolism | 1 | 88 | 0.2519083 | 0.4681859 |
| ko00052 | Galactose metabolism | 1 | 99 | 0.2787268 | 0.4681859 |
| ko00250 | Alanine, aspartate and glutamate metabolism | 1 | 101 | 0.2835025 | 0.4681859 |
| ko03440 | Homologous recombination | 1 | 104 | 0.2906091 | 0.4681859 |
| ko00260 | Glycine, serine and threonine metabolism | 1 | 113 | 0.3115246 | 0.4681859 |
| ko00040 | Pentose and glucuronate interconversions | 1 | 118 | 0.3228868 | 0.4681859 |
| ko00270 | Cysteine and methionine metabolism | 1 | 151 | 0.393479 | 0.5433758 |
| ko00230 | Purine metabolism | 1 | 166 | 0.4231745 | 0.5578209 |
| ko00520 | Amino sugar and nucleotide sugar metabolism | 1 | 218 | 0.515673 | 0.6425247 |
| ko03018 | RNA degradation | 1 | 228 | 0.5317446 | 0.6425247 |
| ko00940 | Phenylpropanoid biosynthesis | 1 | 270 | 0.5938193 | 0.6888304 |
| ko04016 | MAPK signaling pathway - plant | 1 | 290 | 0.62052 | 0.6921185 |
| ko04626 | Plant-pathogen interaction | 1 | 316 | 0.6527169 | 0.7010663 |
| ko01100 | Metabolic pathways | 9 | 3543 | 0.8908952 | 0.900867 |
| ko01110 | Biosynthesis of secondary metabolites | 4 | 1901 | 0.900867 | 0.900867 |

**Supplementary Table 4** The differentially up-regulated genes and the corresponding protein quantification value in PF5411-1.

| Gene ID Maize B73 RefGen_v4 | Protein ID UniProt | Average Protein contents of LH150 (CK) | Average Protein contents of LH150 (HT) | Fold Change of LH150 | Average Protein contents of PF5411-1 (CK) | Average Protein contents of PF5411-1 (HT) | Fold Change of PF5411-1 | Description |
| --- | --- | --- | --- | --- | --- | --- | --- | --- |
| Zm00001d000301 | A0A3L6F4U8 | 156.63 | 172.57 | 1.10 | 152.60 | 159.97 | 1.05 | exocyst subunit exo70 family protein G1 |
| Zm00001d002823 | A0A3L6FTX8 | 4269.33 | 5904.77 | 1.38 | 4021.93 | 7356.03 | 1.83 | Hsp70-Hsp90 organizing protein 3 |
| Zm00001d003164 | B6TLH5 | 103.47 | 154.63 | 1.49 | 80.30 | 94.80 | 1.18 | Ribonucleoside-diphosphate reductase small chain C |
| Zm00001d004243 | A0A3L6FW04 | 1279.23 | 4288.17 | 3.35 | 1380.43 | 3978.13 | 2.88 | Peptidyl-prolyl cis-trans isomerase FKBP65 |
| Zm00001d006821 | B4FS82 | 367.43 | 380.70 | 1.04 | 325.07 | 360.53 | 1.11 | Mitochondrial import inner membrane translocase subunit TIM17-2 |
| Zm00001d008403 | D0QCY9 | 984.83 | 1521.83 | 1.55 | 1146.23 | 1284.23 | 1.12 | Arabidopsis NAC domain containing protein 87 |
| Zm00001d010406 | A0A3L6DKK6 | 2452.00 | 3915.67 | 1.60 | 2071.67 | 2546.07 | 1.23 | Minichromosome maintenance protein |
| Zm00001d012710 | B4FGY0 | 1697.10 | 2461.17 | 1.45 | 1642.80 | 2039.63 | 1.24 | SGS domain-containing protein |
| Zm00001d012837 | B6T2D5 | 421.43 | 425.90 | 1.01 | 302.67 | 405.93 | 1.34 | Histone H2A |
| Zm00001d016512 | C0P9N8 | 1180.27 | 1581.93 | 1.34 | 1085.60 | 1235.63 | 1.14 | Peptidyl-prolyl cis-trans isomerase FKBP65 |
| Zm00001d019163 | B6SRV6 | 143.10 | 196.27 | 1.37 | 157.27 | 223.33 | 1.42 | Stachyose synthase |
| Zm00001d023692 | B6TCP6 | 257.33 | 265.07 | 1.03 | 271.97 | 356.27 | 1.31 | selenium binding |
| Zm00001d023801 | A0A3L6G8W7 | 110.33 | 135.50 | 1.23 | 96.77 | 152.53 | 1.58 | DNA polymerase alpha 2 |
| Zm00001d027524 | B4F9D5 | 790.20 | 1831.37 | 2.32 | 1030.83 | 1208.30 | 1.17 | Basic endochitinase B |
| Zm00001d028408 | B7ZEQ0 | 7236.53 | 24858.17 | 3.44 | 6756.00 | 18828.73 | 2.79 | heat shock protein26 |
| Zm00001d029706 | B6SS87 | 809.07 | 1961.17 | 2.42 | 871.50 | 1277.23 | 1.47 | glutathione transferase39 |
| Zm00001d030614 | C0HFI8 | 864.53 | 1436.13 | 1.66 | 740.57 | 991.13 | 1.34 | DNA replication licensing factor MCM7 |
| Zm00001d030712 | B4FCJ7 | 67.67 | 70.67 | 1.04 | 57.23 | 67.57 | 1.18 | Pectinesterase |
| Zm00001d031569 | B6TI78 | 8363.83 | 11183.97 | 1.34 | 7228.70 | 10791.47 | 1.49 | Peptidyl-prolyl isomerase |
| Zm00001d031740 | A0A317YF45 | 1901.50 | 3372.77 | 1.77 | 1624.20 | 2823.33 | 1.74 | Activator of 90 kDa heat shock protein ATPase |
| Zm00001d032632 | A0A1D6KSD4 | 605.63 | 649.80 | 1.07 | 508.17 | 571.43 | 1.12 | DNA ligase |
| Zm00001d032873 | B6TVS4 | 439.57 | 656.00 | 1.49 | 452.87 | 636.50 | 1.41 | Metal ion binding protein |
| Zm00001d035285 | A0A1D6LFA6 | 281.83 | 325.00 | 1.15 | 236.83 | 276.23 | 1.17 | Heat shock protein 90-6 mitochondrial |
| Zm00001d038583 | A0A1D6M7C8 | 204.50 | 239.97 | 1.17 | 189.23 | 214.50 | 1.13 | Structural maintenance of chromosomes protein |
| Zm00001d038923 | A0A3L6E4Z8 | 2274.63 | 2687.10 | 1.18 | 1717.03 | 1748.20 | 1.02 | Guanine nucleotide-binding protein beta subunit-like protein |
| Zm00001d039566 | B4F9K4 | 1251.63 | 1949.33 | 1.56 | 1620.47 | 3489.50 | 2.15 | 17.5 kDa class II heat shock protein |
| Zm00001d042541 | A0A3L6FBM0 | 630.23 | 2983.23 | 4.73 | 510.00 | 527.90 | 1.04 | lipoxygenase2 |
| Zm00001d044540 | A0A1D6NMY4 | 1289.63 | 2079.10 | 1.61 | 1113.27 | 1319.63 | 1.19 | DNA replication licensing factor MCM4 |
| Zm00001d044728 | B6TQD6 | 412.53 | 922.03 | 2.24 | 457.87 | 1167.57 | 2.55 | Class I heat shock protein 3 |
| Zm00001d045544 | Q6B7Q9 | 885.33 | 1218.10 | 1.38 | 1186.47 | 1689.90 | 1.42 | Chaperonin 60 subunit beta 2 chloroplastic |
| Zm00001d046471 | A0A3L6DDF4 | 815.27 | 1215.90 | 1.49 | 768.70 | 1288.37 | 1.68 | Multiprotein-bridging factor 1c |
| Zm00001d047841 | A0A3L6DEF1 | 971.70 | 3352.43 | 3.45 | 1059.83 | 3218.53 | 3.04 | 17.4 kDa class I heat shock protein |
| Zm00001d048491 | C4J4M8 | 336.10 | 699.80 | 2.08 | 278.63 | 413.83 | 1.49 | Histone H2B |
| Zm00001d049190 | A0A1D6PT08 | 1706.30 | 2467.73 | 1.45 | 1606.10 | 1684.30 | 1.05 | DNA replication licensing factor MCM2 |
| Zm00001d051161 | A0A3L6F5U1 | 240.17 | 332.17 | 1.38 | 292.10 | 374.37 | 1.28 | phenylalanine ammonia lyase3 |
| Zm00001d052021 | A0A1D6QC12 | 1991.67 | 2446.00 | 1.23 | 1832.50 | 2193.00 | 1.20 | DNA replication licensing factor MCM5 |
| Zm00001d053070 | A0A1D6QLT8 | 462.43 | 538.27 | 1.16 | 436.43 | 569.67 | 1.31 | DNA polymerase delta catalytic subunit |
| Zm00001d009888 | B6T4S3 | 622.13 | 542.67 | 0.87 | 437.77 | 511.27 | 1.17 | DNA primase |
| Zm00001d010840 | K7VXF0 | 1646.43 | 1448.83 | 0.88 | 628.07 | 679.57 | 1.08 | triacylglycerol lipase-like 1 |
| Zm00001d017175 | A0A3L6EMZ2 | 671.83 | 650.03 | 0.97 | 635.13 | 697.17 | 1.10 | La protein 1 |
| Zm00001d018839 | A0A1D6HSP4 | 445.50 | 182.97 | 0.41 | 277.60 | 335.90 | 1.21 | cytochrome P-450 19 |
| Zm00001d023309 | B4FAU9 | 310.83 | 263.47 | 0.85 | 244.23 | 256.60 | 1.05 | Sterol 14-demethylase |
| Zm00001d037493 | A0A3L6EEB1 | 851.23 | 704.70 | 0.83 | 807.43 | 937.50 | 1.16 | Heparanase-like protein 3 |
| Zm00001d038453 | A0A3L6EED7 | 370.80 | 323.27 | 0.87 | 923.83 | 1586.33 | 1.72 | Putative aminotransferase class III superfamily protein |
| Zm00001d047424 | A0A3L6DA02 | 527.17 | 493.83 | 0.94 | 562.27 | 809.70 | 1.44 | Flavonoid 3'-monooxygenase |

**Supplementary Table 5** Primers used in the qRT-PCR

| Heat tolerance related genes | Notes | Primer sequence 5'-->3' | |
| --- | --- | --- | --- |
|  |  | Forward primers | Reverse primers |
| *Zm00001d028408* | sHSPs gene | CGCAGGAGAACAGGGACAAC | GGCGACATCGGATCAACTAA |
| *Zm00001d047841* | sHSPs gene | GCAAGGAGCAGGAGGAGAAG | TTAGGCACGGTGACGGTGAG |
| *Zm00001d039566* | sHSPs gene | GACGAGCGGGTGCTGGTGAT | TCTTGTCCATGTCGGCGTTG |
| *Zm00001d044728* | sHSPs gene | AGGACGAGGAGGAGGGGACG | TTGCTGGAGACGGCGATGGA |
| *Zm00001d047424* | Myricetin biosynthesis | GAGTGGGCGATGGCGGAGAT | GGAGAAGTGCGGGAGGCTGA |
| *Zm00001d019163* | Raffinose biosynthesis | AATCAGCGGGAAGGAGGAAG | GAAGGTGAAGACGTGGTGGC |
| *Zm00001d019163* | Raffinose biosynthesis | ACGGGCAACCAGCCGGACAT | GCCCAGCGTCTCGATGATGT |
| *Zm00001d027524* | Ethylene signaling pathway | CAAAGCAAGCCATGTGGAGG | CGATGAAGGCGTCGTAGGTG |
| *Zm00001d027524* | Ethylene signaling pathway | GACCACGAACATCATCAACG | TCATCCTCCGTAAGGCTTCT |
| *ZmGAPDH* | Internal control | CCCTTCATCACCACGGACTAC | AACCTTCTTGGCACCACCCT |
